# Fine-tuned adaptation of embryo–endometrium pairs at implantation revealed by transcriptome analyses in *Bos taurus*

**DOI:** 10.1371/journal.pbio.3000046

**Published:** 2019-04-12

**Authors:** Fernando H. Biase, Isabelle Hue, Sarah E. Dickinson, Florence Jaffrezic, Denis Laloe, Harris A. Lewin, Olivier Sandra

**Affiliations:** 1 Department of Animal Sciences, Auburn University, Auburn, Alabama, United States of America; 2 UMR BDR, INRA, ENVA, Université Paris Saclay, Jouy-en-Josas, France; 3 UMR1313 GABI, INRA, AgroParisTech, Université Paris Saclay, Jouy-en-Josas, France; 4 Department of Evolution and Ecology, University of California, Davis, California, United States of America; Yale University, UNITED STATES

## Abstract

Interactions between embryo and endometrium at implantation are critical for the progression of pregnancy. These reciprocal actions involve exchange of paracrine signals that govern implantation and placentation. However, it remains unknown how these interactions between the conceptus and the endometrium are coordinated at the level of an individual pregnancy. Under the hypothesis that gene expression in endometrium is dependent on gene expression of extraembryonic tissues and genes expressed in extraembryonic tissues are dependent of genes expressed in the endometrium, we performed an integrative analysis of transcriptome profiles of paired extraembryonic tissue and endometria obtained from cattle (*Bos taurus*) pregnancies initiated by artificial insemination. We quantified strong dependence (|r| > 0.95, empirical false discovery rate [eFDR] < 0.01) in transcript abundance of genes expressed in the extraembryonic tissues and genes expressed in the endometrium. The profiles of connectivity revealed distinct coexpression patterns of extraembryonic tissues with caruncular and intercaruncular areas of the endometrium. Notably, a subset of highly coexpressed genes between extraembryonic tissue (*n* = 229) and caruncular areas of the endometrium (*n* = 218, r > 0.9999, eFDR < 0.001) revealed a blueprint of gene expression specific to each pregnancy. Gene ontology analyses of genes coexpressed between extraembryonic tissue and endometrium revealed significantly enriched modules with critical contribution for implantation and placentation, including “in utero embryonic development,” “placenta development,” and “regulation of transcription.” Coexpressing modules were remarkably specific to caruncular or intercaruncular areas of the endometrium. The quantitative association between genes expressed in extraembryonic tissue and endometrium emphasize a coordinated communication between these two entities in mammals. We provide evidence that implantation in mammalian pregnancy relies on the ability of the extraembryonic tissue and the endometrium to develop a fine-tuned adaptive response characteristic of each pregnancy.

## Abstract 2

An integrative analysis of interactions between conceptus and endometrium, in cattle, reveals complex regulatory networks operating at the time of implantation, highlighting the unique transcriptional blueprint of conceptus-maternal communication

## Author summary

Implantation in mammals requires a complex crosstalk between the conceptus (the embryo and associated membranes) and the uterus. An imbalanced regulation of the factors contributing to these interactions has negative impacts on the attachment of the fetus, the progression of the pregnancy, and the progeny. Focusing on paired conceptus–endometrium analyses of individual pregnancies in cows, we have determined that communication at implantation encompasses synchronized genome-wide coregulation of genes. Gene regulatory interactions between one conceptus and the surrounding maternal tissue vary between endometrial regions containing or lacking glands. Our data reveal new insights, to our knowledge, on the coordination of molecular mechanisms that contribute to implantation and pregnancy establishment in mammals. We conclude that the biological response of the endometrium is embryo-specific, a phenomenon that deserves further investigation in the context of assisted reproductive technologies.

## Introduction

In mammals, pregnancy recognition requires a tightly synchronized exchange of signals between the competent embryo and the receptive endometrium. The initiation of this signaling is triggered by key factors produced by the conceptus [1, 2], which are translated by the endometrial cells into actions that will condition the trajectory of embryo development as well as progeny phenotype. In mammalian species, including human, rodents, and ruminants, the delicate balance in embryo–maternal communication is affected by the way the embryos are generated (natural mating, artificial insemination, in vitro fertilization, or somatic cell nuclear transfer) and by the sensor-driver properties of the endometrium defined by intrinsic maternal factors (e.g., maternal metabolism, aging) and environmental perturbations (e.g., pathogens, nutrition) [3–5]. The concept of sensor property applied to the mammalian endometrium was proposed in a former study accompanied by the notion of endometrial plasticity [6]. This property was recently confirmed in vitro with an aberrant responsiveness of human endometrial stromal cultured cells in the context of recurrent pregnancy loss [7]. Nevertheless, it remains unaddressed whether the mammalian endometrium is able to develop an adaptive embryo-tailored response in a normal pregnancy.

In mammalian reproduction, sheep and cattle are research models that have relevantly contributed key insights to the understanding of molecular and physiological pregnancy-associated mechanisms, including the deciphering of embryo–endometrium interactions [8, 9]. In the bovine species, by gestation days 7–8, the blastocyst enters the uterine lumen. After hatching by days 8–9, the outer monolayer of trophectoderm cells establishes direct contact with the luminal epithelium of the endometrium [10]. On gestation days 12–13, the blastocyst is ovoid in shape (approximately 2–5 mm) and transitions into a tubular shape by days 14–15. Next, the conceptus begins to elongate via proliferation of the trophectoderm and parietal endoderm cells [11]. The bovine extraembryonic tissue reaches 30 cm or more in length by days 19–20 [11, 12], and the trophectoderm begins to attach to the luminal epithelium (LE) of the endometrium, which marks the beginning of the attachment and onset of placentation [11].

By approximately day 15, rapidly proliferating trophectoderm cells of the extraembryonic tissues synthesize and release interferon tau (IFNT) [12–16], which is the major pregnancy recognition signal in ruminants [1, 9, 17, 18]. The disrupted release of the oxytocin-dependent pulses of prostaglandin F2 alpha [19] allows maintenance of progesterone production by a functional corpus luteum [19], which is critical for the establishment and progression of pregnancy [1, 4, 9, 12, 15, 16, 20]. IFNT actions include induction of numerous classical and nonclassical IFNT-stimulated genes and stimulation of progesterone-induced genes that encode proteins involved in conceptus elongation and implantation [4]. IFNT-regulated genes have diverse actions in the endometrium that are essential for conceptus survival and pregnancy establishment [12]. Other paracrine signals such as prostaglandins and cortisol have regulatory effects on conceptus elongation and endometrium remodeling [21]. More recently, the identification of potential ligand-receptor interactions between the conceptus and endometrium [22] and the secretion of proteins and RNAs through exosomes [23, 24] have expanded the field of possibilities by which the conceptus and endometrium interact prior to and during implantation.

The crosstalk between the conceptus and the endometrium is associated with the expression and regulation of a wealth of genes in each entity [25, 26]. The nature of the conceptus modifies gene expression of the endometrium in cattle [6, 27, 28] and decidualizing human endometrial stromal cells [29]. Similarly, the endometrium from dams with different fertility potentials [30] or metabolic status [31] influences the gene expression of the conceptus. Despite the growing evidence of the interactions between conceptus and endometrium at the level of gene regulation, the pathways and the functions that result from this interaction have yet to be unveiled. Furthermore, the lack of integrated analysis between paired conceptus and endometrium has made it challenging to advance our understanding of the functional interactions between these two entities in normal pregnancies.

Here, we hypothesized that gene expression of extraembryonic tissue is not independent from gene expression of endometrium. In the present study, we carried out an integrative analysis of transcriptome profiles of paired conceptuses and endometria at the onset of implantation, aiming at the identification of regulatory pathways that have coordinated expression between the conceptus and endometrium in normal pregnancies. Surprisingly, our results show that at gestation day 18 in cattle, several hundred genes have an expression profile in the conceptus and caruncular areas of the endometrium that is unique to each pregnancy. Analyses of genes coexpressed between the conceptus and the paired-associated endometrium revealed significantly enriched gene coexpression modules for specific biological processes with critical contribution for implantation and placentation. Our data provide evidence that successful implantation in mammalian pregnancy relies on the ability of the endometrium to elicit a fine-tuned adaptive response to the conceptus.

## Results

### Data overview

**Fig 1:**
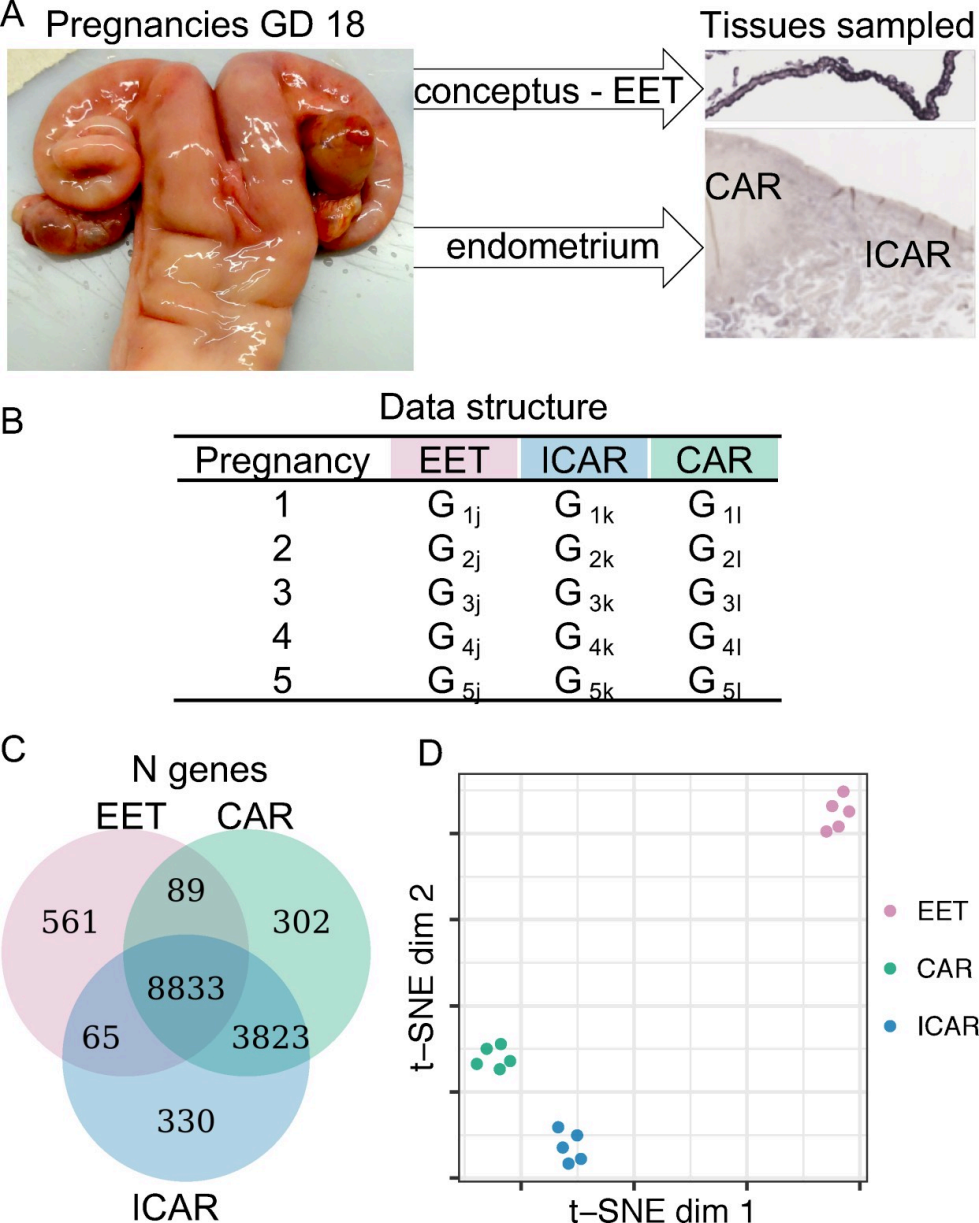
Transcriptome profiling of extraembryonic tissue and endometrium collected from gestation day 18. (A) Representative images of pregnant uterus and micrograph identifying the tissues from which RNA-seq data were used in this study (CAR and ICAR tissues and EET, which was composed of parietal endoderm, mesoderm, and trophectoderm). (B) Data structure used in this study. Data on genome-wide transcript abundance were obtained from EET and endometrium (CAR and ICAR tissues) from five pregnant uteri. (C) Number of genes with transcript abundance quantified in each sample. (D) Dimensionality reduction of the RNA-seq data for special visualization of the sample distribution. The data underlying Fig 1D can be obtained with the scripts presented in S1 Code. CAR, caruncular; dim, dimension; EET, extraembryonic tissue; GD, gestation day; ICAR, intercaruncular; RNA-seq, RNA-sequencing; t-SNE, t-distributed stochastic neighbor embedding.

We analyzed the RNA-sequencing (RNA-seq) data, which consisted of samples collected from five cattle pregnancies terminated at gestation day 18 (Gene Expression Omnibus database GSE74152 [27]). The conceptus was dissected, and transcriptome data were generated for extraembryonic tissue, whereas the endometrium was dissected into caruncular (gland-free) and intercaruncular (containing endometrial glands) areas, and transcriptome data were generated from both regions of the endometrium (Fig 1A). Therefore, the data set analyzed was comprised of three sample types collected from each pregnancy: extraembryonic, caruncular, and intercaruncular tissues (Fig 1B). Alignment of the sequences to the *B*. *taurus* genome (University of Maryland [UMD] assembly 3.1) resulted into an average of 22, 31.4, and 34.6 million uniquely mapped reads for extraembryonic (*n* = 5), caruncular (*n* = 5), and intercaruncular (*n* = 5) tissues, respectively. After filtering for lowly expressed genes, we estimated the transcript abundance of 9,548, 13,047, and 13,051 genes in extraembryonic, caruncular, and intercaruncular tissues, respectively (Fig 1C). Unsupervised clustering of the samples based on their transcriptome data separated the samples obtained from the extraembryonic tissue from the endometrial samples and further distinguished caruncular from intercaruncular endometrial samples (Fig 1D).

### Correlated gene expression between extraembryonic tissue and endometrium

The associated expression between two genes can be assessed by correlative metrics [32] within [33, 34] or between tissues [34, 35]. Thus, we calculated Pearson’s coefficient of correlation (r [36]) to test whether there is association between the transcript abundance of genes expressed in extraembryonic tissue and endometrium (caruncular or intercaruncular tissues). We reasoned that under a null hypothesis, the abundance of a gene expressed in extraembryonic tissue (G_j_) would have no association with the abundance of a gene expressed in endometrium (G_k_, or G_l_), e.g., *H*_0_:*r*_*(Gj*,*Gk)*_ ≈ 0. On the other hand, under the alternative hypothesis (*H*_1_:*r*_*(Gj*,*Gk)*_ ≠ 0), two genes display coexpression [36].

**Fig 2:**
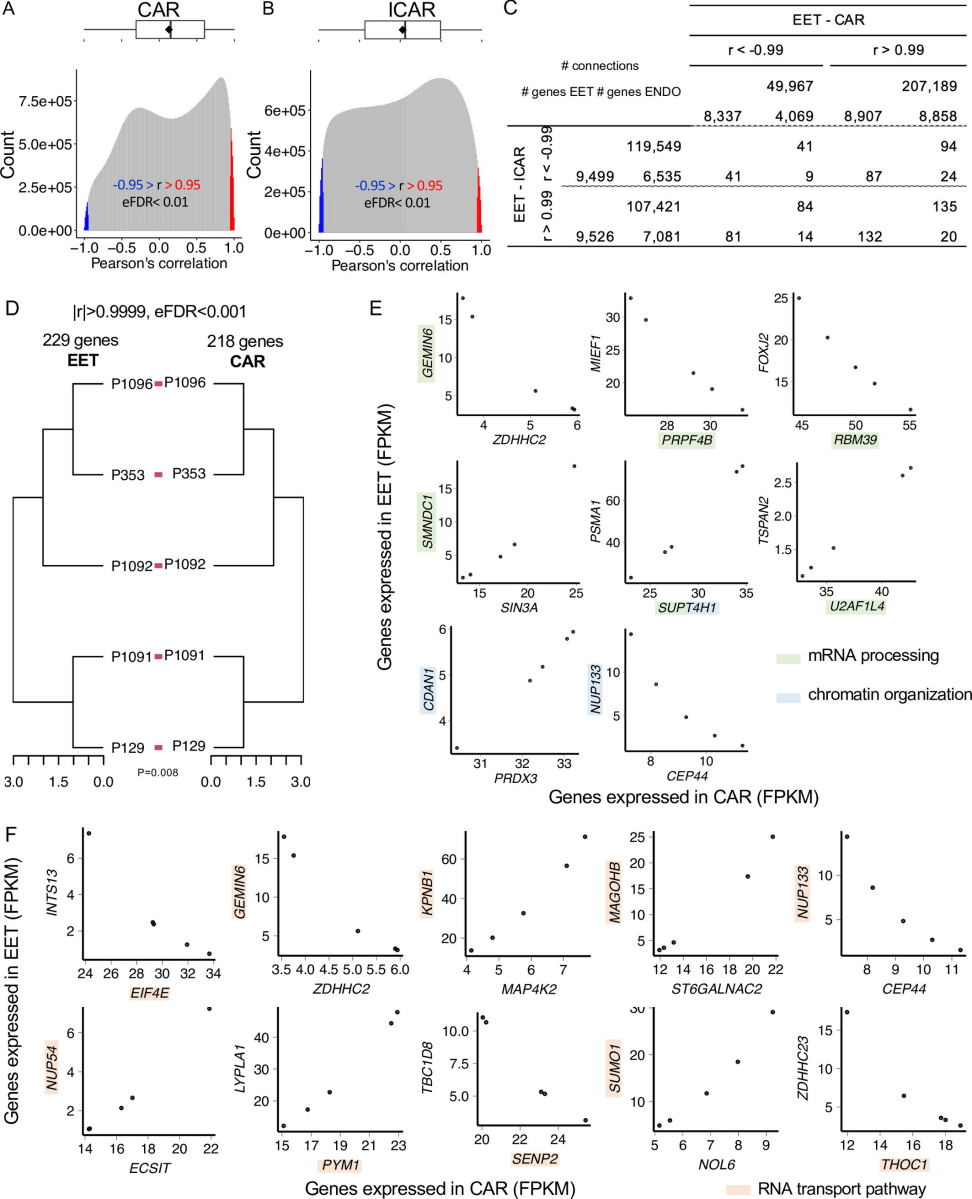
Coexpression analysis between EET and ENDO. Distribution of the correlation coefficients for genes expressed in EET and CAR tissues (A) or ICAR tissues (B). (C) Number of genes expressed in EET, CAR tissues, or ICAR tissues that participate in significant correlation connections involving EET and ENDO. (D) Dendrogram of EET and CAR tissues formed by genes with strong coexpression. (E) Scatterplot of pairs of genes expressed in EET and CAR tissues with at least one gene involved in “mRNA processing” or “chromatin organization.” (F) Scatterplot of pairs of genes expressed in EET and CAR tissues and highly correlated with at least one gene involved in “RNA transport pathway.” The data underlying each panel on Fig 2 can be obtained with the scripts presented on S1 Code. CAR, caruncular; *CDAN1*, codanin 1; *CEP44*, centrosomal protein 44; *ECSIT*, ECSIT signaling integrator; EET, extraembryonic tissue; eFDR, empirical false discovery rate; *EIF4E*, eukaryotic translation initiation factor 4E; ENDO, endometrium; FPKM, fragments per kilobase per million reads; *FOXJ2*, forkhead box J2; *GEMIN6*, gem-nuclear-organelle–associated protein 6; ICAR, intercaruncular; *INTS13*, integrator complex subunit 13; *KPNB1*, karyopherin subunit beta 1; *LYPLA1*, acyl-protein thioesterase 1; *MAGOHB*, protein mago nashi homolog 2; *MAP4K2*, mitogen-activated protein kinase kinase kinase kinase 2; *MIEF1*, mitochondrial elongation factor 1; *NOL6*, nucleolar protein 6; *NUP133*, nucleoporin 133; *PRDX3*, peroxiredoxin 3; *PRPF4B*, pre-mRNA processing factor 4B; *PSMA1*, proteasome subunit alpha type-1; *PYM1*, PYM homolog 1, exon-junction-complex–associated factor; *RBM39*, RNA-binding motif protein 39; *SENP2*, SUMO1/sentrin/SMT3-specific peptidase 2; *SIN3A*, SIN3 transcription regulator family member A; *SMNDC1*, survival motor neuron domain containing 1; *ST6GALNAC2*, ST6 N-acetylgalactosaminide alpha-2,6-sialyltransferase 4; *SUMO1*, small ubiquitin-like modifier 1; *SUPT4H1*, SPT4 homolog, DSIF elongation factor subunit; *TBC1D8*, TBC1 domain family member 8; *THOC1*, THO complex 1; *TSPAN2*, tetraspanin 2; *U2AF1L4*, splicing factor U2AF 26-kDa subunit; *ZDHHC2*, zinc finger DHHC-type containing 2.

The distribution of correlation coefficients for all pairs of genes expressed in extraembryonic and caruncular tissues averaged 0.13 (Fig 2A), and the equivalent distribution obtained for all pairs of genes expressed in extraembryonic and intercaruncular tissues averaged 0.03 (Fig 2B). Both distributions deviated significantly from a distribution obtained from shuffled data that disrupted the pairing of the extraembryonic tissue and endometrium (*P* < 2.2^−16^, S1 Fig). We calculated the empirical false discovery rate (eFDR) and noted that absolute correlation coefficients in both distributions were highly significant when greater than 0.95 (eFDR < 0.007, S2 Fig and S1 Table). The pairs of genes presenting significant correlation on the paired data rarely reoccurred when we scrambled the pregnancy pairs (S1 Table). Of note, S3 and S4 Figs present examples of pairs of genes we identified with the highest positive and negative correlation coefficients, which fit the alternative hypothesis (*H*_1_:*r*_*(Gj*,*Gk)*_ ≠ 0), and examples of pairs of genes that show correlation coefficients close to zero, fitting the null hypothesis (*H*_0_:*r*_*(Gj*,*Gk)*_ ≈ 0).

The distribution of degrees of connectivity for significant correlations (|r| > 0.95, eFDR < 0.01) between extraembryonic and caruncular tissues was not equivalent to the distribution observed between extraembryonic and intercaruncular tissues (*P* < 2.2^−16^). On average, genes expressed in extraembryonic tissue were significantly correlated with 295 genes expressed in caruncular tissues (median = 101). Eleven genes were significantly correlated with over 2,300 genes in caruncular tissues (i.e., amphiregulin [*AREG*], early growth response 1 [*EGR1*], peroxisomal biogenesis factor 3 [*PEX3*], gigaxonin [*GAN*], S5A Fig). On average, genes expressed in extraembryonic tissue were significantly correlated with 266 genes expressed in intercaruncular tissues (median = 252). Eight genes were significantly correlated with over 750 genes in intercaruncular tissues (i.e., wingless/Integrated family member 5B [*WNT5B*], *WNT7B*, receptor-tyrosine-kinase–like orphan receptor 2 [*ROR2*], dipeptidase 1 [*DPEP1*], gap junction protein beta 3 [*GJB3*], S5B Fig). These results strongly suggest different patterns of gene coexpression between extraembryonic and caruncular or intercaruncular tissues.

When considering highly significant correlations (r > 0.99 or r < -0.99, eFDR < 0.001), notably, over 99% of the genes expressed in extraembryonic tissue were positively or negatively correlated with genes expressed in intercaruncular tissues (Fig 2C). Of the genes expressed in extraembryonic tissues, 93% and 87% were positively or negatively correlated with genes expressed in intercaruncular tissues, respectively. Of the genes expressed in caruncular tissues, 31% and 67% negatively or positively correlated with genes expressed in extraembryonic tissues, respectively. Similarly, 50% and 54% of the genes expressed in intercaruncular tissues were negatively and positively correlated with genes expressed in extraembryonic tissues, respectively (Fig 2C). These gene pairs rarely maintained their highly significant correlation when the pregnancy pair was disrupted (S1 Table). Thus, highly significant coexpression between thousands of genes is a consequence of the interaction between the conceptus and the endometrium.

We then examined whether genes coexpressed in extraembryonic tissue and endometrium have expression patterns that are unique to pregnancies. We identified 229 and 218 genes expressed in extraembryonic and caruncular tissues, respectively (|r| > 0.9999, eFDR < 0.0001, S1 Table), whose expression profiles produced equivalent dendrograms for extraembryonic and caruncular tissues independently (*P* = 0.008, Fig 2D). This set of genes consisted of 223 and 212 genes expressed exclusively in extraembryonic and caruncular tissue, respectively, and six genes that were expressed in both compartments. At this level of significance, no gene pairs retained their correlation in the shuffled data (S1 Table).

Gene ontology analysis of these 441 genes identified significant enrichment in the biological processes “mRNA processing” (gem-nuclear-organelle–associated protein 6 [*GEMIN6*]; pre-mRNA processing factor 4B [*PRPF4B*]; RNA-binding motif protein 39 [*RBM39*]; survival motor neuron domain containing 1 [*SMNDC1*]; SPT4 homolog, DSIF elongation factor subunit [*SUPT4H1*]; splicing factor U2AF 26-kDa subunit [*U2AF1L4*]; FDR = 0.13, Fig 2E), “chromatin organization” (codanin 1 [*CDAN1*], nucleoporin 133 [*NUP133*], *SUPT4H1*, FDR = 0.13, Fig 2E), and “protein autoubiquitination” (CCR4-NOT transcription complex subunit 4 [*CNOT4*], membrane-associated ring-CH–type finger 5 [*MARCH5*], ubiquitin-like with PHD and ring finger domains 1 [*UHRF1*]). We also interrogated the Kyoto Encyclopedia of Genes and Genomes (KEGG) pathways database and identified an enrichment for the “RNA transport” pathway (eukaryotic translation initiation factor 4E binding protein 1 [*EIF4E*], *GEMIN6*, karyopherin subunit beta 1 [*KPNB1*], protein mago nashi homolog 2 [*MAGOHB*], *NUP133*, *NUP54*, PYM homolog 1, exon-junction-complex–associated factor [*PYM1*], SUMO1/sentrin/SMT3-specific peptidase 2 [*SENP2*], small ubiquitin-like modifier 1 [*SUMO1*], THO complex 1 [*THOC1*], FDR = 0.06, Fig 2F). A bootstrapping approach (2,000 randomizations of 441 genes) showed a probability <0.001 of these two biological processes being enriched by chance (S6A Fig). Similarly, there was <0.001 probability of the “RNA transport” pathway to have been enriched by chance (S6B Fig). We did not identify groups of genes coexpressed in extraembryonic and intercaruncular tissues capable of producing dendrograms that mirrored each other. These results demonstrate that genes highly coexpressed between extraembryonic and caruncular tissues form a signature that independently distinguishes pregnancies in an equivalent manner.

### Visualization of coexpressed networks in extraembryonic tissue and endometrium

Our analysis was not an exhaustive evaluation of all potential coexpression networks that exist between extraembryonic tissue and the endometrium. Thus, we developed a web interface for dynamic and interactive data visualization based on the coexpression analysis conducted in the present study [37, 38] (https://biaselab.shinyapps.io/eet_endo/). The public access to this web application allows a user to produce networks for genes of their choosing. Furthermore, each network is accompanied by supporting data such as scatter plots and heatmaps of the gene-expression values. The raw data and codes for reproduction of this interface can be downloaded from a GitHub repository (https://github.com/BiaseLab/eet_endo_gene_interaction).

### Gene coexpression networks between extraembryonic and caruncular tissues

We investigated the transcriptome-wide interactions between extraembryonic and caruncular or intercaruncular tissues independently. The clustering of genes based on coexpression is a powerful means to understand coordinated gene functions [39]; thus, we used the matrix with correlation coefficients to cluster extraembryonic, caruncular, and intercaruncular tissues independently.

**Fig 3:**
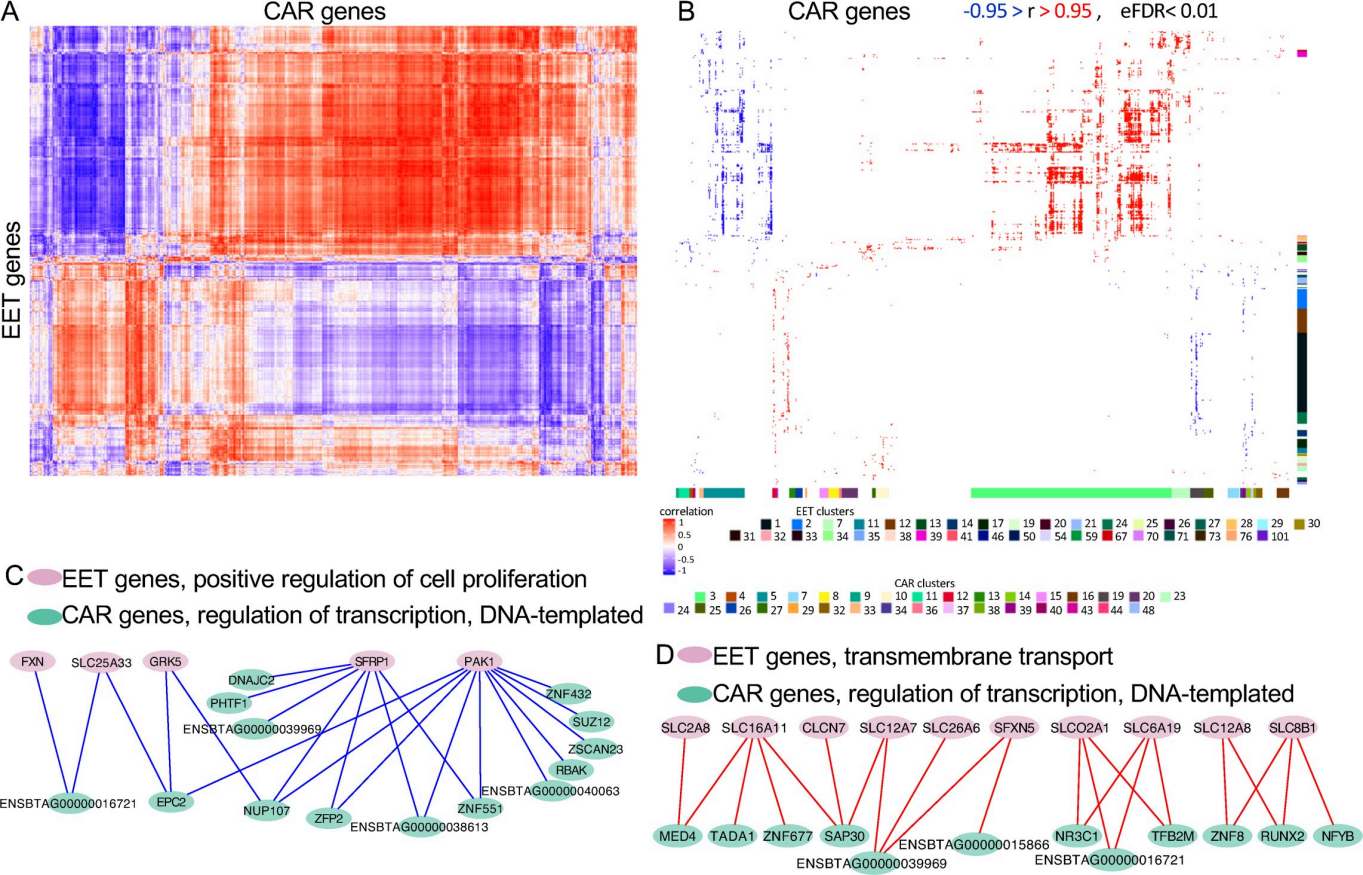
Analysis of coexpressed genes between EET and CAR tissues. (A) Heatmap produced by the correlation coefficients and independent clustering of EET and CAR tissues. (B) Gene ontology analysis of the cluster formed. Only significant coefficients of correlation are shown (|r| > 0.95, eFDR < 0.01). The colored bar on the right of the heatmap indicates clusters of genes expressed in EET for which biological processes were significant. The colored bar on bottom of the heatmap indicates clusters of genes expressed in caruncle for which biological processes were significant. The colored squares at the bottom of the image identify the cluster number with the color observed on the bars. See S1 and S2 Datas for details on the cluster identification, biological processes, and genes. (C, D) Model of gene coexpression networks possibly formed between EET and CAR tissues. The data underlying each panel on Fig 3 can be obtained with the scripts presented in S1 Code. CAR, caruncular; *CLCN*, chloride voltage-gated channels; *DNAJC2*, DnaJ homolog subfamily C member 2; EET, extraembryonic tissue; eFDR, empirical false discovery rate; *EPC2*, enhancer of polycomb homolog 2; *FXN*, frataxin; *GRK5*, G-protein–coupled receptor kinase 5; *MED4*, mediator complex subunit 4; *NFYB*, nuclear transcription factor Y subunit beta; *NR3C1*, nuclear receptor subfamily 3 group C member 1; *NUP107*, nucleoporin 107; *PAK1*, serine/threonine-protein kinase PAK 1; *PHTF1*, putative homeodomain transcription factor 1; *RBAK*, RB-associated KRAB zinc finger: *RUNX2*, runt-related transcription factor 2; *SAP30*, Sin3A-associated protein 30; *SFRP1*, secreted frizzled related protein 1; *SFXN5*, sideroflexin 5; SLC, solute carrier; *SLCO2A1*, solute carrier organic anion transporter family member 2A1; *SUZ12*, SUZ12 polycomb repressive complex 2 subunit; *TADA1*, transcriptional adaptor 1; TFB2M, transcription factor B2, mitochondrial; *ZFP2*, ZFP2 zinc finger protein; ZNF, zinc finger; *ZSCAN23*, zinc finger and SCAN domain containing 23.

The heatmap resulting from clustering the two data sets (extraembryonic and caruncular tissues) showed the formation of an organized coexpression network between the genes expressed in extraembryonic and caruncular tissues (Fig 3A). We identified 36 clusters formed by the genes expressed in extraembryonic tissue that presented enrichment for several biological processes (FDR < 0.2, Fig 3B), in which we identified several genes expressed in extraembryonic tissue significantly coexpressed with genes expressed in caruncular tissues (see S1 Data for a list of genes). For instance, 142 genes associated with regulation of transcription were identified across clusters 1, 12, 30, 38, and 54. Eighty-two genes were associated with signal transduction across clusters 1, 21, 27, and 71. Interestingly, 26 genes associated with “in utero embryonic development” were identified in cluster 1.

The clustering of genes expressed in caruncular tissues according to their coexpression with extraembryonic tissue genes resulted in the identification of 32 clusters presenting enrichment (FDR < 0.2) for several biological processes (Fig 3A and S2 Data). Among the genes forming significant coexpression with extraembryonic tissue, we identified 96 genes in cluster 3 associated with “intracellular protein transport,” as well as 111 and 4 genes associated with regulation of transcription on clusters 4 and 5, respectively. Notably, 10 genes on cluster 15 were associated with “defense response to virus,” and the annotated genes are known to be stimulated by IFNT (interferon-induced protein with tetratricopeptide repeats 1 [*IFIT1*], *IFIT3*, *IFIT5*, *ISG15*, MX-dynamin–like GTPase 1 [*MX1*], *MX2*, 2′-5′-oligoadenylate synthetase 1 [*OAS1Y*], radical S-adenosyl methionine domain containing 2 [*RSAD2*]; S2 Data).

Next, we intersected the results of gene ontology enrichment obtained from clustering extraembryonic and caruncular tissues. We identified several biological processes on both data sets with coexpressing genes expressed in extraembryonic and caruncular tissues (S3 Data). Based on the number of genes and direction of connections, two pairs of biological processes are noteworthy. First, five genes associated with “positive regulation of cell proliferation” in extraembryonic tissue form negative coexpression connections (${\overset{-}{x}}_{r}\;=\;-0.96$, *n* = 22) with 14 genes associated with “regulation of transcription, DNA-templated” expressed in caruncle (Fig 3C). Second, 10 genes associated with “transmembrane transport” in extraembryonic tissue form positive coexpression connections (${\overset{-}{x}}_{r}\;=\;0.97$, *n* = 22) with 12 genes associated with “regulation of transcription, DNA-templated” expressed in caruncle (Fig 3D). These results are coherent with a coexpression between genes expressed in extraembryonic and caruncular tissues, with biological implications to extraembryonic tissue attachment and implantation.

### Gene coexpression networks between extraembryonic and intercaruncular tissues

**Fig 4:**
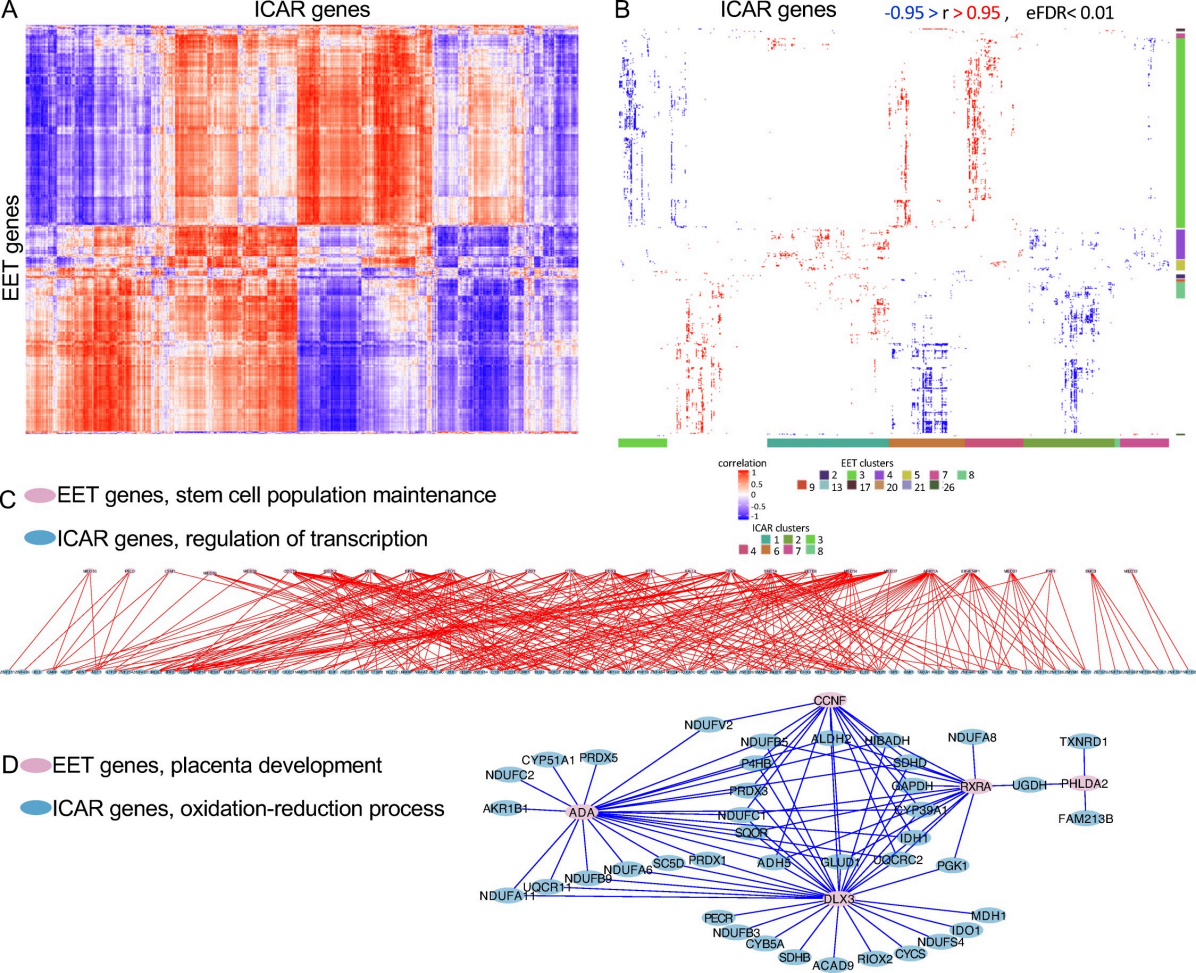
Analysis of coexpressed genes between EET and ICAR tissues. (A) Heatmap produced by the correlation coefficients and independent clustering of EET and ICAR tissues. (b) Gene ontology analysis of the cluster formed. Only significant coefficients of correlation are shown (|r| > 0.95, eFDR < 0.01). The colored bar on the right of the heatmap indicates clusters of genes expressed in EET for which biological processes were significant. The colored bar on bottom of the heatmap indicates clusters of genes expressed in ICAR tissues for which biological processes were significant. The colored squares at the bottom of the image identify the cluster number with the color observed on the bars. See S4 and S5 Datas for details on the cluster identification, biological processes, and genes. (C, D) Model of coexpression networks possibly formed between EET and ICAR tissues. See S7 Fig for an enlarged version of panel C. The data underlying each panel on Fig 4 can be obtained with the scripts presented on S1 Code. *ACAD9*, acyl-CoA dehydrogenase family member 9, mitochondrial; *ADA*, adenosine deaminase; *ADH5*, alcohol dehydrogenase class-3; *ALDH2*, aldehyde dehydrogenase, mitochondrial; *CCNF*, cyclin F; CYCS, cytochrome c; *CYP51A1*, lanosterol 14-alpha demethylase; *DLX3*, distal-less homeobox 3; EET, extraembryonic tissue; eFDR, empirical false discovery rate; *FAM213B*, family with sequence similarity 213 member B; *GAPDH*, glyceraldehyde-3-phosphate dehydrogenase; *GLUD1*, glutamate dehydrogenase 1, mitochondrial precursor; *HIBADH*, 3-hydroxyisobutyrate dehydrogenase; ICAR, intercaruncular; *IDH1*, isocitrate dehydrogenase (NADP(+)) 1, cytosolic; *IDO1*, indoleamine 2,3-dioxygenase 1; *MDH1*, malate dehydrogenase 1; NDUF, nicotinamide adenine dinucleotide + hydrogen:ubiquinone oxidoreductase subunit; *PECR*, peroxisomal trans-2-enoyl-CoA reductase; *PGK1*, phosphoglycerate kinase 1; *PHLDA2*, pleckstrin-homology–like domain family A member 2; *PRDX*, peroxiredoxin; *P4HB*, prolyl 4-hydroxylase subunit beta; *RIOX2*, ribosomal oxygenase 2; RXRA, retinoid X receptor alpha; *SC5D*, sterol-C5-desaturase; *SDHB*, succinate dehydrogenase complex iron sulfur subunit B; *SDHD*, succinate dehydrogenase complex iron sulfur subunit D; *SQOR*, sulfide:quinone oxidoreductase, mitochondrial; *TXNRD*, thioredoxin reductase 1; *UGDH*, UDP-glucose 6-dehydrogenase.

The independent clustering of the correlation coefficients obtained from the genes expressed in extraembryonic and intercaruncular tissues also evidenced an organized coexpression network between the two tissues (Fig 4A). Twelve clusters formed by genes expressed in the extraembryonic tissue presented enrichment for biological processes (FDR < 0.2, Fig 4B; see S4 Data for a list of genes). Interestingly, there were 85 and 27 genes associated with “mRNA processing” and “stem cell population maintenance,” respectively, on cluster 3. On cluster 5, we identified 12 genes associated with “negative regulation of cell proliferation” and seven genes associated with “regulation of receptor activity.” On cluster 8, five genes were associated with “placenta development” (adenosine deaminase [*ADA*], cyclin F [*CCNF*], distal-less homeobox 3 [*DLX3*], pleckstrin-homology–like domain family A member 2 [*PHLDA2*], and retinoid X receptor alpha [*RXRA*]). On cluster 17, eight genes were associated with “regulation of transcription, DNA-templated.”

The clusters formed by intercaruncular genes coexpressed with extraembryonic tissue genes also highlighted significant enrichment of biological processes (FDR < 0.2, Fig 4A; see S5 Data for a list of genes). For instance, clusters 1 and 6 contained 145 and 63 genes associated with regulation of transcription, respectively. Interestingly, on cluster 2, there were 149, 23, 22, and 16 genes associated with “oxidation-reduction process,” “cell redox homeostasis,” “electron transport chain,” and “tricarboxylic acid cycle.” Cluster 4 contained 63 genes associated with “regulation of transcription,” and cluster 7 contained 11 genes associated with “fatty acid beta oxidation.”

The intersection of the genes identified in enriched biological processes in clusters formed by extraembryonic and intercaruncular tissues revealed several coexpression networks between these two tissues (S6 Data) that have critical implications for implantation. Notably, several of the intersecting categories involved processes associated with regulation of transcription or oxidation reduction on the intercaruncular side. For instance, 28 genes associated with “stem cell population maintenance” and expressed in extraembryonic tissue presented positive coexpression (${\overset{-}{x}}_{r}\;=\;0.97$, *n* = 305) with 83 genes associated with “regulation of transcription” and expressed in intercaruncular tissues (Fig 4D). Five genes associated with “placenta development” and expressed in extraembryonic tissue presented negative coexpression (${\overset{-}{x}}_{r}\;=\;-0.97$, *n* = 88) with 41 genes associated with “oxidation-reduction process” and expressed in intercaruncular tissues (Fig 4E).

## Discussion

In mammals and particularly in cattle, a large body of gene-expression data was produced at various steps of early pregnancy derived from in vitro- or in vivo-produced embryos [6, 27, 28, 40], varied physiological status of the dam [41], and fertility-classified heifers [30]. Altogether, results based on group analyses (extraembryonic tissue or endometrium) have demonstrated different degrees of interactions between the extraembryonic tissue and endometrium at the initial phases of implantation. In the present study, our objective was to shed light on the subtle interactions between the extraembryonic tissue of a conceptus and the endometrial tissue of the uterus hosting this conceptus in normal pregnancy using paired coexpression analyses of gene transcript abundances. Our analyses were carried out using biological material collected from the single conceptus and the endometrium from the same pregnancy, a critical aspect to determine the crosstalk during implantation at the level of one individual pregnant female.

It must be noted that our study has some limitations that may reduce the extent of the insights into individual pregnancies. First, we worked with samples collected at one developmental stage (gestation day 18). Gestation is a highly dynamic process; thus, we can anticipate that the gene interactions will also be dynamic. Second, the endometrium is a tissue diverse in cell types (e.g., epithelial lumen, stromal tissue, immune cells, glandular epithelia) that were not dissected at collection. Therefore, we did not dissect the cellular origin of the signals. This work provides a snapshot of the rich and unique interactions between extraembryonic and endometrial tissues at the tissue level that will deserve to be refined at the cell-type level.

Our analyses of transcriptome data from extraembryonic tissue and endometrium pairs identified key signatures of gene expression that are likely to be linked to the success of pregnancy recognition and implantation. A large proportion of all genes quantified in extraembryonic tissue and endometrium have transcript abundances that were not independent. Furthermore, the dependency observed for the abundance of transcripts between extraembryonic tissue and endometrium varied with morphologically and physiologically distinct areas of the endometrium, namely caruncular and intercaruncular tissues. For instance, there were twice as many highly positive (r > 0.95) and approximately half the number of highly negative (r < -0.95) coexpressing connections between extraembryonic and caruncular tissues compared to extraembryonic and intercaruncular tissues. These results greatly expand previous findings that the extraembryonic tissue triggers distinct molecular responses in caruncular and intercaruncular tissues [6, 27, 42, 43].

During the elongation phase, the mural trophoblast proliferates rapidly [12, 26, 44] while maintaining its pluripotency [45]. This period of development is modulated by dynamic regulation of gene expression [44] whereby metabolically active trophoblastic cells [46, 47] rely on the uptake of nutrients from the uterine luminal fluid [48]. Our results show that caruncular and intercaruncular tissues have an active role in the programing of those functions because several genes related to gene regulation, signal transduction, cellular proliferation, maintenance of stem cell population, and transmembrane transport are also coexpressed with genes expressed in the endometrium. The importance of gene coregulation between extraembryonic tissue and endometrium was further supported by the identification of 26 genes associated with “in utero embryonic development” and five genes associated with “placenta development” coregulated with genes expressed in caruncle and intercaruncle, respectively.

Among the genes expressed in caruncular or intercaruncular tissues that were coexpressed with extraembryonic tissues, it was noticeable that several genes were associated with regulation of gene expression. This finding is in line with former publications suggesting that the regulatory network needed for endometrial remodeling [49] during attachment is extraembryonic-tissue dependent [6, 27, 28, 40]. In the caruncular tissue, we specifically identified 15 genes associated with “defense response to virus,” of which eight genes had their expression modulated by IFNT, produced by the trophoblast between gestation days 9 and 25 [50]. This result provides additional knowledge on the biological actions of IFNT and other extraembryonic-tissue–originated signaling on the remodeling of the caruncle [51].

Our findings identified genes with high levels of coexpression (|r| > 0.9999) between extraembryonic tissue (*n* = 229) and endometrial caruncular tissues (*n* = 218) whose transcript profiles independently produced equivalent discrimination of the pregnancies. Gene ontology analysis of these 441 genes revealed that highly coexpressed genes between extraembryonic and caruncular tissues are involved in regulatory functions at the chromatin, mRNA processing, and protein levels, which is a strong indication of a coordinated reprogramming of tissues driven by multiple layers of cell regulation during the conceptus–maternal recognition. These data prompt the need for additional investigation to better define the coordinated interactions between extraembryonic tissues and endometrium at the level of tissue layer including luminal epithelium, stroma, and glandular epithelium.

In the intercaruncular tissues, our analyses identified a list of genes related with “oxidation-reduction process,” a finding consistent with a recent publication reporting that proteins associated with oxidation reduction are enriched in the uterine luminal fluid on gestation day 16 in cattle [52]. Oxidative stress is a consequence of altered oxidation-reduction state [53], and transcriptional regulation of factors involved in the regulation of oxidative stress has been reported in the bovine endometrium during the estrous cycle and early pregnancy [42, 54], Furthermore, a significant increase in oxidation-reduction potential was observed in the endometrium of mice prior to implantation [55]. The results show evidence that the maintenance of oxidation-reduction status permissive to the conceptus health [56] and implantation is strongly linked to genes regulated in intercaruncular tissues of the endometrium in cattle.

**Fig 5:**
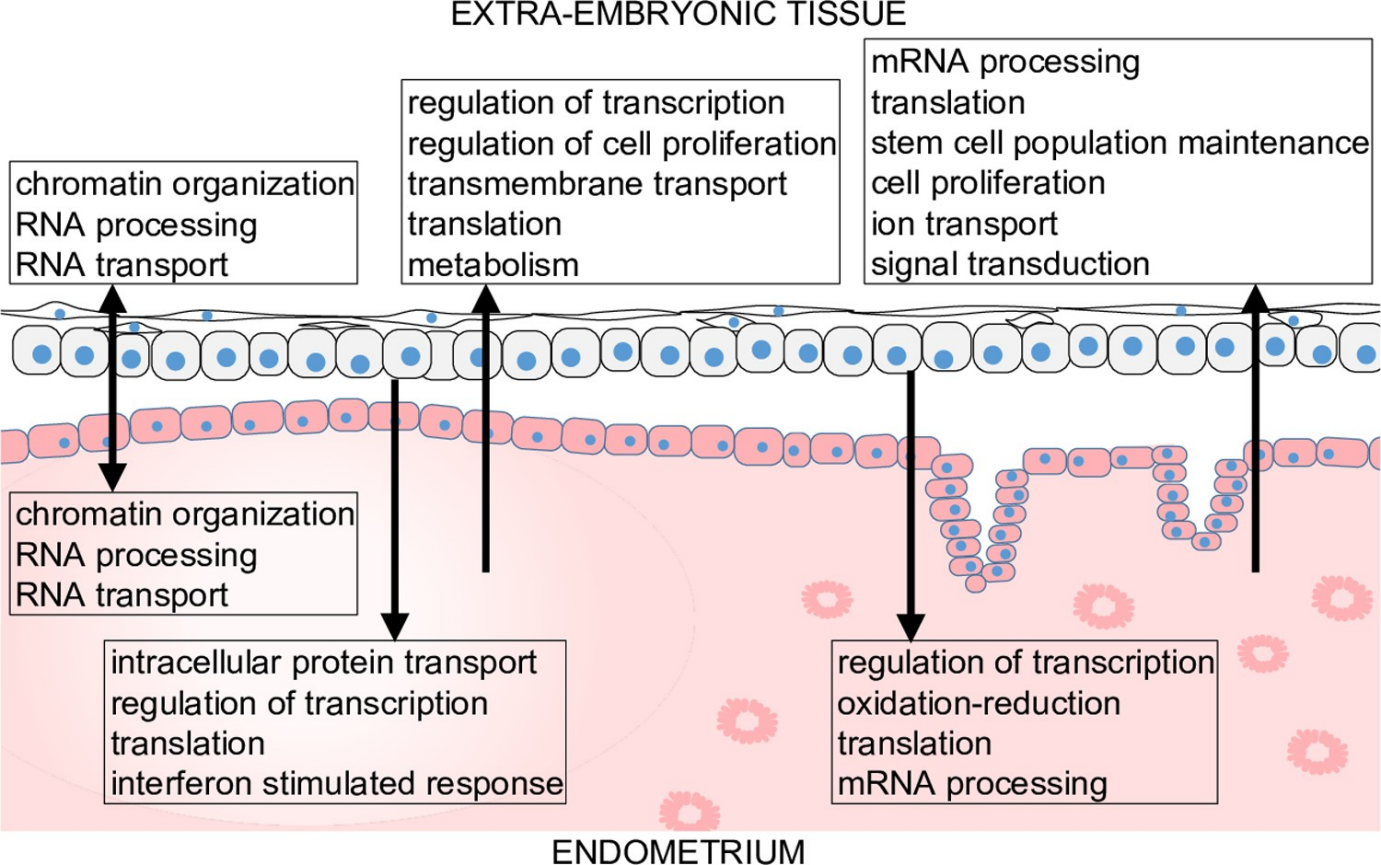
Working model of most prominent biological functions modulated by coexpression between extraembryonic tissue and endometrium. The arrows indicate probable direction of interaction.

The analyses carried out in this study have provided novel, to our knowledge, insights into the molecular interactions between extraembryonic, caruncular, and intercaruncular tissues, summarized in Fig 5. Gene products expressed by the extraembryonic tissue impact the endometrial function by regulating diverse cell functions including oxidative stress, chromatin remodeling, gene transcription, and mRNA processing and translation. The endometrium also exerts key regulatory roles on the extraembryonic tissue cells by modulating chromatin remodeling, gene transcription, cell proliferation, translation, metabolism, and signaling (Fig 5). Collectively, our data have shown that endometrial plasticity, a notion first suggested in cattle [6], allows unique adaptive and coordinated conceptus-matched interactions at implantation in nonpathological pregnancies.

This study presents an analysis of paired extraembryonic tissue and endometrium in a mammalian species, using an integrative systems biology approach. A more comprehensive understanding of the connection between the conceptus and the endometrium at the gene-expression level will open new venues for the development of strategies to improve term pregnancy rates when artificial reproductive technologies are used. Since the endometrial response is embryo-specific, it would be valuable to develop approaches aiming at selection of a competent embryo better suited for the establishment of a successful crosstalk with the recipient uterus of the female considered for transfer.

## Materials and methods

### Ethics statement

This work was performed on publicly available data [27]. The initial work with animals was carried out with approval of the INRA Ethics Committee.

### Data analyzed and estimation of gene-expression levels

All analytical procedures were carried out in R software [37]. The files and codes for full reproducibility of the results are listed in the file S1 Code.

The appropriate approval from institutional committees of ethical oversight for animal use in research was obtained as reported previously [27]. All five cattle (*B*. *taurus*, Holstein breed) gestations were initiated by artificial insemination using semen from a single bull and later terminated on gestation day 18 for sample collection. We analyzed RNA-seq data (100-base long reads, GSE74152 [27]) generated from samples obtained from cattle gestations interrupted at day 18 (*n* = 5). The samples were extraembryonic tissue (*n* = 5), caruncle (*n* = 5), and intercaruncle (*n* = 5) regions from the endometrium.

The reads were aligned to the bovine genome (*B*. *taurus*, UMD 3.1) using STAR aligner [57]. Reads that aligned at one location of the genome with fewer than four mismatches were retained for elimination of duplicates. Nonduplicated reads were used for estimation of fragments per kilobase per million reads (FPKM) using Cufflinks (v. 2.2.1 [58]) and Ensembl gene models [59]. Genes were retained for downstream analyses if FPKM > 1 in ≥4 samples. We employed the t-Distributed Stochastic Neighbor Embedding approach [60] to assess the relatedness of the tissues.

### Calculation of correlation of gene expression between tissues

Three sample types were collected from the same pregnancy (extraembryonic tissue and caruncular and intercaruncular tissues); thus, the data structure (Fig 1B) allowed us to quantify the association between genes expressed in extraembryonic tissue and the endometrium (caruncular and intercaruncular tissues). We utilized Pearson’s coefficient of correlation because of its sensitivity to outliers [61] to calculate $r_{\left(G_{j},G_{k}\right)}$ and $r_{\left(G_{j},G_{l}\right)}$, where *G*_*j*_, *G*_*k*_, and *G*_*l*_ are the transcript abundance of a gene expressed in extraembryonic tissue, caruncle, and intercaruncle, respectively.

To assess the significance of the correlations observed in the real data set, we calculated eFDR by permuting the pregnancy index (*i* = 1,…,5) for the extraembryonic tissue samples, thereby breaking the pairing of extraembryonic tissue and endometrium obtained per pregnancy (B = 100 permutations). The proportion of correlations resulting from the scrambled data that was greater than a specific threshold was calculated as follows: $\overset{B}{\underset{b=1}{\sum }}\frac{\#\left(m:\left|r_{\left(G_{ji},G_{ki}\right)}^{0b}\right|\geq \left|r_{\left(G_{ji},G_{ki}\right)}^{scrambled}\right|,\;m=1,\ldots ,\;\left(k\times j\right)\right)+1}{\left(k\times j\times B\right)+1}$ [34, 62, 63] for extraembryonic and caruncular tissues. Similar calculation was executed for extraembryonic and intercaruncular tissues.

### Testing the resemblance of two distance matrices

We calculated distance matrices for extraembryonic tissue and caruncle based on the Pearson’s coefficient of correlation of the expressed genes within tissues. The correlation matrix was subtracted from one to obtain a distance matrix, which was used as input for clustering using the method “complete.” We used the Mantel statistic test implemented in the “mantel” package to assess the correlation between the two dissimilarity matrices. The significance of the Mantel statistic was assessed by a permutation approach.

### Clustering of samples, heatmaps, and network visualization

We clustered samples using the “flashClust” package [64]; we used the “ComplexHeatmaps” package [65] to draw annotated heatmaps and Cytoscape software [66] to visualize the networks.

### Testing for enrichment of gene ontology terms or KEGG pathways

We tested for enrichment of gene ontology [67] categories and KEGG pathways [68] using the “goseq” package [69]. Subsets of genes were defined according to appropriate thresholds and defined as “test genes”; the genes expressed in the corresponding tissue were then used as background for the calculation of significance values [70]. Significance values were then adjusted for FDR according to the Benjamini and Hochberg method [71]. We further tested the likelihood of significant categories of gene ontology or KEGG pathways to have been identified by chance by a bootstrapping approach. We selected tested genes randomly (2,000 rounds of randomized subsetting) from the genes expressed and carried out the procedure for detection of enrichment described above. Then, we calculated the proportion of FDR values observed from the randomizations that were lower than the result observed from the real data.

## Supporting information

S1 Fig Distribution of Pearson’s coefficient of correlation from shuffled data. The underlying data can be obtained with the scripts presented in S1 Code.
(PDF)

S2 Fig Distribution of eFDR calculated for different levels of correlation. The underlying data can be obtained with the scripts presented in S1 Code. eFDR, empirical false discovery rate
(PDF)

S3 Fig Examples of pairs of genes expressed in EET and CAR tissues fitting the null or alternative hypothesis for coexpression. The underlying data can be obtained with the scripts presented in S1 Code. CAR, caruncular; EET, extraembryonic tissue
(PDF)

S4 Fig Examples of pairs of genes expressed in EET and ICAR tissues fitting the null or alternative hypothesis for coexpression. The underlying data can be obtained with the scripts presented in S1 Code. EET, extraembryonic tissue; ICAR, intercaruncular
(PDF)

S5 Fig Distribution of number of genes presenting a coexpression relationship between EET and the endometrium. (a) EET and CAR tissues. (b) EET and ICAR tissues. The underlying data can be obtained with the scripts presented in S1 Code. CAR, caruncular; EET, extraembryonic tissue; ICAR, intercaruncular
(PDF)

S6 Fig Validation of GO and KEGG enrichment analysis by bootstrapping procedures. (A) Distribution of false discovery values for all categories of biological process tested. The middle and bottom panels show the distribution of values of false discovery rate for the categories “mRNA processing” (middle) and “chromatin organization” (bottom). (B) Distribution of false discovery values for all KEGG terms tested. The bottom panel show the distribution of values of FDR for the pathway “RNA transport.” Red dot represents the FDR obtained from the real data, and black dots represent FDRs obtained from the genes sampled randomly from the data set. The underlying data can be obtained with the scripts presented in S1 Code. FDR, false discovery rate; GO, gene ontology; KEGG, Kyoto Encyclopedia of Genes and Genomes
(PDF)

S7 Fig Enlarged version of Fig 4C. The underlying data can be obtained with the scripts presented in S1 Code.
(PDF)

S1 Table Number of genes involved in highly correlated expression levels between EET and endometrial regions in cattle on gestation day 18. The underlying data can be obtained with the scripts presented on S1 Code. EET, extraembryonic tissue
(PDF)

S1 Code Codes utilized to generate the results obtained from this study. (HTML)

S1 Data Biological processes significantly enriched in clusters composed by genes expressed in EET and coexpressed with genes expressed in CAR tissue. CAR, caruncular; EET, extraembryonic tissue
(XLSX)

S2 Data Biological processes significantly enriched in clusters composed by genes expressed in CAR tissue and coexpressed with genes expressed in EET. CAR, caruncular; EET, extraembryonic
(XLSX)

S3 Data Coexpression-based intersection between biological processes enriched in EET and CAR tissue. CAR, caruncular; EET, extraembryonic tissue
(XLSX)

S4 Data Biological processes significantly enriched in clusters composed by genes expressed in EET and coexpressed with genes expressed in ICAR tissue. EET, extraembryonic tissue; ICAR, intercaruncular
(XLSX)

S5 Data Biological processes significantly enriched in clusters composed by genes expressed in ICAR tissue and coexpressed with genes expressed in EET. EET, extraembryonic tissue; ICAR, intercaruncular
(XLSX)

S6 Data Coexpression-based intersection between biological processes enriched in EET and ICAR tissue. EET, extraembryonic tissue; ICAR, intercaruncular
(XLSX)

## Abbreviations

ADA: adenosine deaminase
AREG: amphiregulin
CCNF: cyclin F
CDAN1: codanin 1
CNOT4: CCR4-NOT transcription complex subunit 4
DLX3: distal-less homeobox 3
DPEP1: dipeptidase 1
eFDR: empirical false discovery rate
EGR1: early growth response 1
EIF4E: eukaryotic translation initiation factor 4E binding protein 1
FPKM: fragments per kilobase per million reads
GD: gestation day
GAN: gigaxonin
GEMIN6: gem-nuclear-organelle–associated protein 6
GJB3: gap junction protein beta 3
IFIT: interferon-induced protein with tetratricopeptide repeats
IFNT: interferon tau
ISG15: ISG15 ubiquitin-like modifier
KEGG: Kyoto Encyclopedia of Genes and Genomes
KPNB1: karyopherin subunit beta 1
LE: luminal epithelium
MAGOHB: protein mago nashi homolog 2
MARCH5: membrane-associated ring-CH–type finger 5
MX: MX-dynamin–like GTPase
NUP133: nucleoporin 133
OAS1Y: 2′-5′-oligoadenylate synthetase 1
PEX3: peroxisomal biogenesis factor 3
PHLDA2: pleckstrin-homology–like domain family A member 2
PRPF4B: pre-mRNA processing factor 4B
PYM1: PYM homolog 1, exon-junction-complex–associated factor
RBM39: RNA-binding motif protein 39
RNA-seq: RNA-sequencing
ROR2: receptor-tyrosine-kinase–like orphan receptor 2
RSAD2: radical S-adenosyl methionine domain containing 2
RXRA: retinoid X receptor alpha
SENP2: SUMO1/sentrin/SMT3-specific peptidase 2
SMNDC1: survival motor neuron domain containing 1
ST6GALNAC2: ST6 N-acetylgalactosaminide alpha-2,6-sialyltransferase 4
SUMO1: small ubiquitin-like modifier 1
SUPT4H1: SPT4 homolog, DSIF elongation factor subunit
t-SNE: t-distributed stochastic neighbor embedding
THOC1: THO complex 1
UHRF1: ubiquitin-like with PHD and ring finger domains 1
UMD: University of Maryland
U2AF1L4: splicing factor U2AF 26-kDa subunit
WNT: wingless/Integrated family member

## References

[1] Raheem KA. An insight into maternal recognition of pregnancy in mammalian species. Journal of the Saudi Society of Agricultural Sciences. 2017;16(1):1–6. 10.1016/j.jssas.2015.01.002

[2] Bazer FW. History of Maternal Recognition of Pregnancy. Adv Anat Embryol Cell Biol. 2015;216:5–25. Epub 2015/10/10. 10.1007/978-3-319-15856-3_2 . 26450492

[3] Macklon NS, Brosens JJ. The human endometrium as a sensor of embryo quality. Biol Reprod. 2014;91(4):98 Epub 2014/09/05. 10.1095/biolreprod.114.122846 . 25187529

[4] Sandra O, Charpigny G, Galio L, Hue I. Preattachment Embryos of Domestic Animals: Insights into Development and Paracrine Secretions. Annu Rev Anim Biosci. 2017;5:205–28. Epub 2016/12/14. 10.1146/annurev-animal-022516-022900 . 27959670

[5] Sandra O, Mansouri-Attia N, Lea RG. Novel aspects of endometrial function: a biological sensor of embryo quality and driver of pregnancy success. Reprod Fertil Dev. 2011;24(1):68–79. Epub 2012/03/08. 10.1071/RD11908 . 22394719

[6] Mansouri-Attia N, Sandra O, Aubert J, Degrelle S, Everts RE, Giraud-Delville C, et al Endometrium as an early sensor of in vitro embryo manipulation technologies. P Natl Acad Sci USA. 2009;106(14):5687–92. Epub 2009/03/20. 10.1073/pnas.0812722106 19297625 PMC2667091

[7] Lucas ES, Dyer NP, Murakami K, Lee YH, Chan YW, Grimaldi G, et al Loss of Endometrial Plasticity in Recurrent Pregnancy Loss. Stem Cells. 2016;34(2):346–56. 10.1002/stem.2222 PubMed PMID: WOS:000370353200009. 26418742

[8] Lee KY, DeMayo FJ. Animal models of implantation. Reproduction. 2004;128(6):679–95. Epub 2004/12/08. 10.1530/rep.1.00340 . 15579585

[9] Bazer FW. Pregnancy recognition signaling mechanisms in ruminants and pigs. J Anim Sci Biotechnol. 2013;4(1):23 Epub 2013/06/27. 10.1186/2049-1891-4-23 23800120 PMC3710217

[10] Carson DD, Bagchi I, Dey SK, Enders AC, Fazleabas AT, Lessey BA, et al Embryo implantation. Dev Biol. 2000;223(2):217–37. Epub 2000/07/07. 10.1006/dbio.2000.9767 . 10882512

[11] Hue I, Degrelle SA, Turenne N. Conceptus elongation in cattle: genes, models and questions. Anim Reprod Sci. 2012;134:19–28. 10.1016/j.anireprosci.2012.08.007 . 22921267

[12] Spencer TE, Hansen TR. Implantation and Establishment of Pregnancy in Ruminants. Adv Anat Embryol Cell Biol. 2015;216:105–35. Epub 2015/10/10. 10.1007/978-3-319-15856-3_7 . 26450497

[13] Robinson RS, Hammond AJ, Wathes DC, Hunter MG, Mann GE. Corpus luteum-endometrium-embryo interactions in the dairy cow: underlying mechanisms and clinical relevance. Reprod Domest Anim. 2008;43 Suppl 2:104–12. Epub 2008/07/25. 10.1111/j.1439-0531.2008.01149.x . 18638111

[14] Roberts RM. Interferon-tau, a Type 1 interferon involved in maternal recognition of pregnancy. Cytokine Growth Factor Rev. 2007;18(5–6):403–8. Epub 2007/07/31. 10.1016/j.cytogfr.2007.06.010 17662642 PMC2000448

[15] Imakawa K, Bai R, Nakamura K, Kusama K. Thirty years of interferon-tau research; Past, present and future perspective. Anim Sci J. 2017;88(7):927–36. Epub 2017/05/16. 10.1111/asj.12807 . 28504476

[16] Martal J, Chene N, Camous S, Huynh L, Lantier F, Hermier P, et al Recent developments and potentialities for reducing embryo mortality in ruminants: the role of IFN-tau and other cytokines in early pregnancy. Reprod Fertil Dev. 1997;9(3):355–80. Epub 1997/01/01. . 9261883 10.1071/r96083

[17] Spencer TE, Burghardt RC, Johnson GA, Bazer FW. Conceptus signals for establishment and maintenance of pregnancy. Anim Reprod Sci. 2004;82–83:537–50. Epub 2004/07/24. 10.1016/j.anireprosci.2004.04.014 . 15271478

[18] Wooding P, Burton G. Comparative Placentation: Structures, Functions and Evolution. Berlin: Springer Verlag; 2008.

[19] Meyer MD, Hansen PJ, Thatcher WW, Drost M, Badinga L, Roberts RM, et al Extension of Corpus Luteum Lifespan and Reduction of Uterine Secretion of Prostaglandin F2α of Cows in Response to Recombinant Interferon-τ. J Dairy Sci. 1995;78(9):1921–31. 10.3168/jds.S0022-0302(95)76817-5 8550901

[20] Roberts RM, Chen Y, Ezashi T, Walker AM. Interferons and the maternal-conceptus dialog in mammals. Semin Cell Dev Biol. 2008;19(2):170–7. Epub 2007/11/23. 10.1016/j.semcdb.2007.10.007 18032074 PMC2278044

[21] Brooks K, Burns G, Spencer TE. Conceptus elongation in ruminants: roles of progesterone, prostaglandin, interferon tau and cortisol. J Anim Sci Biotechnol. 2014;5(1):53 Epub 2014/01/01. 10.1186/2049-1891-5-53 25810904 PMC4373033

[22] Mamo S, Mehta JP, Forde N, McGettigan P, Lonergan P. Conceptus-endometrium crosstalk during maternal recognition of pregnancy in cattle. Biol Reprod. 2012;87(1):6, 1–9. Epub 2012/04/21. 10.1095/biolreprod.112.099945 . 22517619

[23] Burns G, Brooks K, Wildung M, Navakanitworakul R, Christenson LK, Spencer TE. Extracellular vesicles in luminal fluid of the ovine uterus. PLoS ONE. 2014;9(3):e90913 Epub 2014/03/13. 10.1371/journal.pone.0090913 24614226 PMC3948691

[24] Burns GW, Brooks KE, Spencer TE. Extracellular Vesicles Originate from the Conceptus and Uterus During Early Pregnancy in Sheep. Biol Reprod. 2016;94(3):56 Epub 2016/01/29. 10.1095/biolreprod.115.134973 . 26819476

[25] Bazer FW, Wu G, Spencer TE, Johnson GA, Burghardt RC, Bayless K. Novel pathways for implantation and establishment and maintenance of pregnancy in mammals. Mol Hum Reprod. 2010;16(3):135–52. Epub 2009/11/03. 10.1093/molehr/gap095 19880575 PMC2816171

[26] Spencer TE, Forde N, Lonergan P. Insights into conceptus elongation and establishment of pregnancy in ruminants. Reprod Fertil Dev. 2016;29(1):84–100. Epub 2016/01/01. 10.1071/RD16359 . 28278796

[27] Biase FH, Rabel C, Guillomot M, Hue I, Andropolis K, Olmstead CA, et al Massive dysregulation of genes involved in cell signaling and placental development in cloned cattle conceptus and maternal endometrium. P Natl Acad Sci USA. 2016;113(51):14492–501. Epub 2016/12/13. 10.1073/pnas.1520945114 27940919 PMC5187692

[28] Bauersachs S, Ulbrich SE, Zakhartchenko V, Minten M, Reichenbach M, Reichenbach HD, et al The endometrium responds differently to cloned versus fertilized embryos. P Natl Acad Sci USA. 2009;106(14):5681–6. Epub 2009/03/25. 10.1073/pnas.0811841106 19307558 PMC2666995

[29] Brosens JJ, Salker MS, Teklenburg G, Nautiyal J, Salter S, Lucas ES, et al Uterine selection of human embryos at implantation. Sci Rep. 2014;4:3894 Epub 2014/02/08. 10.1038/srep03894 24503642 PMC3915549

[30] Moraes JGN, Behura SK, Geary TW, Hansen PJ, Neibergs HL, Spencer TE. Uterine influences on conceptus development in fertility-classified animals. P Natl Acad Sci USA. 2018;115(8):E1749–E58. Epub 2018/02/13. 10.1073/pnas.1721191115 29432175 PMC5828633

[31] Forde N, Simintiras CA, Sturmey RG, Graf A, Wolf E, Blum H, et al Effect of lactation on conceptus-maternal interactions at the initiation of implantation in cattle: I. Effects on the conceptus transcriptome and amino acid composition of the uterine luminal fluid. Biol Reprod. 2017;97(6):798–809. Epub 2017/11/01. 10.1093/biolre/iox135 . 29088315

[32] de Siqueira Santos S, Takahashi DY, Nakata A, Fujita A. A comparative study of statistical methods used to identify dependencies between gene expression signals. Brief Bioinform. 2014;15(6):906–18. Epub 2013/08/22. 10.1093/bib/bbt051 . 23962479

[33] Obayashi T, Hayashi S, Shibaoka M, Saeki M, Ohta H, Kinoshita K. COXPRESdb: a database of coexpressed gene networks in mammals. Nucleic Acids Res. 2008;36(Database issue):D77–82. Epub 2007/10/13. 10.1093/nar/gkm840 17932064 PMC2238883

[34] Biase FH, Kimble KM. Functional signaling and gene regulatory networks between the oocyte and the surrounding cumulus cells. BMC Genomics. 2018;19(1):351 Epub 2018/05/12. 10.1186/s12864-018-4738-2 29747587 PMC5946446

[35] Kogelman LJA, Fu JY, Franke L, Greve JW, Hofker M, Rensen SS, et al Inter-Tissue Gene Co-Expression Networks between Metabolically Healthy and Unhealthy Obese Individuals. PLoS ONE. 2016;11(12):e0167519. doi: ARTN e0167519 10.1371/journal.pone.0167519 PubMed PMID: WOS:000389482700180. 27907186 PMC5132173

[36] Song L, Langfelder P, Horvath S. Comparison of co-expression measures: mutual information, correlation, and model based indices. BMC Bioinformatics. 2012;13:328 Epub 2012/12/12. 10.1186/1471-2105-13-328 23217028 PMC3586947

[37] Ihaka R, Gentleman. R: A Language and Environment for Statistical Computing. J Comput Graph Stat. 1995;5:299–14. doi: {ISBN} 3-900051-07-0.

[38] Chang W, Cheng J, Allaire J, Xie Y, McPherson J. shiny: Web Application Framework for R. R package version 1.0.5. https://cran.r-project.org/web/packages/shiny/index.html.

[39] Swift S, Tucker A, Vinciotti V, Martin N, Orengo C, Liu X, et al Consensus clustering and functional interpretation of gene-expression data. Genome Biol. 2004;5(11):R94 Epub 2004/11/13. 10.1186/gb-2004-5-11-r94 15535870 PMC545785

[40] Biase FH, Rabel C, Guillomot M, Sandra O, Andropolis K, Olmstead C, et al Changes in WNT signaling-related gene expression associated with development and cloning in bovine extra-embryonic and endometrial tissues during the peri-implantation period. Mol Reprod Dev. 2013;80(12):977–87. Epub 2013/09/17. 10.1002/mrd.22257 . 24038527

[41] Valour D, Degrelle SA, Ponter AA, Giraud-Delville C, Campion E, Guyader-Joly C, et al Energy and lipid metabolism gene expression of D18 embryos in dairy cows is related to dam physiological status. Physiol Genomics. 2014;46(2):39–56. Epub 2013/11/14. 10.1152/physiolgenomics.00091.2013 . 24220328

[42] Mansouri-Attia N, Aubert J, Reinaud P, Giraud-Delville C, Taghouti G, Galio L, et al Gene expression profiles of bovine caruncular and intercaruncular endometrium at implantation. Physiol Genomics. 2009;39(1):14–27. Epub 2009/07/23. 10.1152/physiolgenomics.90404.2008 . 19622795

[43] Walker CG, Meier S, Littlejohn MD, Lehnert K, Roche JR, Mitchell MD. Modulation of the maternal immune system by the pre-implantation embryo. BMC Genomics. 2010;11:474 Epub 2010/08/17. 10.1186/1471-2164-11-474 20707927 PMC3091670

[44] Blomberg L, Hashizume K, Viebahn C. Blastocyst elongation, trophoblastic differentiation, and embryonic pattern formation. Reproduction. 2008;135(2):181–95. Epub 2008/02/02. 10.1530/REP-07-0355 . 18239048

[45] Pfeffer PL, Pearton DJ. Trophoblast development. Reproduction. 2012;143(3):231–46. Epub 2012/01/10. 10.1530/REP-11-0374 . 22223687

[46] Houghton FD. Energy metabolism of the inner cell mass and trophectoderm of the mouse blastocyst. Differentiation. 2006;74(1):11–8. Epub 2006/02/10. 10.1111/j.1432-0436.2006.00052.x . 16466396

[47] Bax BE, Bloxam DL. Energy metabolism and glycolysis in human placental trophoblast cells during differentiation. Biochim Biophys Acta. 1997;1319(2–3):283–92. Epub 1997/04/11. . 9131049 10.1016/s0005-2728(96)00169-7

[48] Bazer FW, Johnson GA, Wu G. Amino acids and conceptus development during the peri-implantation period of pregnancy. Adv Exp Med Biol. 2015;843:23–52. Epub 2015/05/10. 10.1007/978-1-4939-2480-6_2 . 25956294

[49] King GJ, Atkinson BA, Robertson HA. Development of the bovine placentome from days 20 to 29 of gestation. J Reprod Fertil. 1980;59(1):95–100. Epub 1980/05/01. . 7401049 10.1530/jrf.0.0590095

[50] Kimura K, Spate LD, Green MP, Murphy CN, Seidel GE Jr., Roberts RM. Sexual dimorphism in interferon-tau production by in vivo-derived bovine embryos. Mol Reprod Dev. 2004;67(2):193–9. Epub 2003/12/25. 10.1002/mrd.10389 . 14694435

[51] Atkinson BA, King GJ, Amoroso EC. Development of the caruncular and intercaruncular regions in the bovine endometrium. Biol Reprod. 1984;30(3):763–74. Epub 1984/04/01. . 6722243 10.1095/biolreprod30.3.763

[52] Forde N, McGettigan PA, Mehta JP, O'Hara L, Mamo S, Bazer FW, et al Proteomic analysis of uterine fluid during the pre-implantation period of pregnancy in cattle. Reproduction. 2014;147(5):575–87. Epub 2014/01/31. 10.1530/REP-13-0010 . 24478148

[53] Sies H. Oxidative stress: a concept in redox biology and medicine. Redox Biol. 2015;4:180–3. Epub 2015/01/16. 10.1016/j.redox.2015.01.002 25588755 PMC4309861

[54] Lesage-Padilla A, Forde N, Poiree M, Healey GD, Giraud-Delville C, Reinaud P, et al Maternal metabolism affects endometrial expression of oxidative stress and FOXL2 genes in cattle. PLoS ONE. 2017;12(12):e0189942 Epub 2017/12/28. 10.1371/journal.pone.0189942 29281695 PMC5744954

[55] Nakamura H, Hosono T, Kumasawa K, Kimura T. Prospective evaluation of uterine receptivity in mice. Reprod Fertil Dev. 2017;30(4):619–623. Epub 2017/09/25. 10.1071/RD17209 . 28941468

[56] Yoon SB, Choi SA, Sim BW, Kim JS, Mun SE, Jeong PS, et al Developmental Competence of Bovine Early Embryos Depends on the Coupled Response Between Oxidative and Endoplasmic Reticulum Stress. Biol Reprod. 2014;90(5):104. Epub 2014/04/04. doi: ARTN 104 10.1095/biolreprod.113.113480 PubMed PMID: WOS:000336792100001. 24695629

[57] Dobin A, Davis CA, Schlesinger F, Drenkow J, Zaleski C, Jha S, et al STAR: ultrafast universal RNA-seq aligner. Bioinformatics. 2013;29(1):15–21. Epub 2012/10/30. 10.1093/bioinformatics/bts635 23104886 PMC3530905

[58] Trapnell C, Roberts A, Goff L, Pertea G, Kim D, Kelley DR, et al Differential gene and transcript expression analysis of RNA-seq experiments with TopHat and Cufflinks. Nat Protoc. 2012;7(3):562–78. Epub 2012/03/03. 10.1038/nprot.2012.016 22383036 PMC3334321

[59] Flicek P, Amode MR, Barrell D, Beal K, Billis K, Brent S, et al Ensembl 2014. Nucleic Acids Res. 2014;42(Database issue):D749–55. Epub 2013/12/10. 10.1093/nar/gkt1196 24316576 PMC3964975

[60] van der Maaten LJP, Hinton GE. Visualizing High-Dimensional Data Using t-SNE. J Mach Learn Res. 2008;9:2579–605.

[61] Serin EA, Nijveen H, Hilhorst HW, Ligterink W. Learning from Co-expression Networks: Possibilities and Challenges. Front Plant Sci. 2016;7:444 Epub 2016/04/20. 10.3389/fpls.2016.00444 27092161 PMC4825623

[62] Storey JD, Tibshirani R. Statistical significance for genomewide studies. P Natl Acad Sci USA. 2003;100(16):9440–5. Epub 2003/07/29. 10.1073/pnas.1530509100 12883005 PMC170937

[63] Sham PC, Purcell SM. Statistical power and significance testing in large-scale genetic studies. Nat Rev Genet. 2014;15(5):335–46. Epub 2014/04/18. 10.1038/nrg3706 24739678

[64] Langfelder P, Horvath S. Fast R Functions for Robust Correlations and Hierarchical Clustering. J Stat Softw. 2012;46(11). Epub 2012/10/11. 23050260 PMC3465711

[65] Gu Z, Eils R, Schlesner M. Complex heatmaps reveal patterns and correlations in multidimensional genomic data. Bioinformatics. 2016;32(18):2847–9. Epub 2016/05/22. 10.1093/bioinformatics/btw313 . 27207943

[66] Cline MS, Smoot M, Cerami E, Kuchinsky A, Landys N, Workman C, et al Integration of biological networks and gene expression data using Cytoscape. Nat Protoc. 2007;2(10):2366–82. Epub 2007/10/20. 10.1038/nprot.2007.324 17947979 PMC3685583

[67] Ashburner M, Ball CA, Blake JA, Botstein D, Butler H, Cherry JM, et al Gene ontology: tool for the unification of biology. The Gene Ontology Consortium. Nat Genet. 2000;25(1):25–9. Epub 2000/05/10. 10.1038/75556 10802651 PMC3037419

[68] Du J, Yuan Z, Ma Z, Song J, Xie X, Chen Y. KEGG-PATH: Kyoto encyclopedia of genes and genomes-based pathway analysis using a path analysis model. Mol Biosyst. 2014;10(9):2441–7. Epub 2014/07/06. 10.1039/c4mb00287c . 24994036

[69] Young MD, Wakefield MJ, Smyth GK, Oshlack A. Gene ontology analysis for RNA-seq: accounting for selection bias. Genome Biol. 2010;11(2):R14 Epub 2010/02/06. 10.1186/gb-2010-11-2-r14 20132535 PMC2872874

[70] Timmons JA, Szkop KJ, Gallagher IJ. Multiple sources of bias confound functional enrichment analysis of global -omics data. Genome Biol. 2015;16:186 Epub 2015/09/09. 10.1186/s13059-015-0761-7 26346307 PMC4561415

[71] Benjamini Y, Hochberg Y. Controlling the False Discovery Rate—a Practical and Powerful Approach to Multiple Testing. J Roy Stat Soc B Met. 1995;57(1):289–300. PubMed PMID: WOS:A1995QE45300017.

