# Supplementary figures and images for: Fine-tuned adaptation of embryo–endometrium pairs at implantation revealed by transcriptome analyses in Bos taurus
Source: PLoS Biol. 2019 Apr 12;17(4):e3000046. doi: 10.1371/journal.pbio.3000046 (PMC6481875; doi:10.1371/journal.pbio.3000046)

EET and CAR

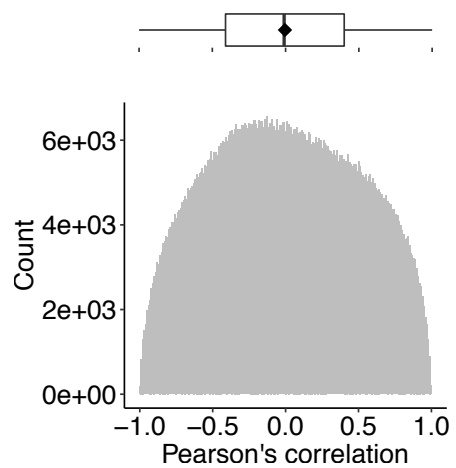

EET and CAR

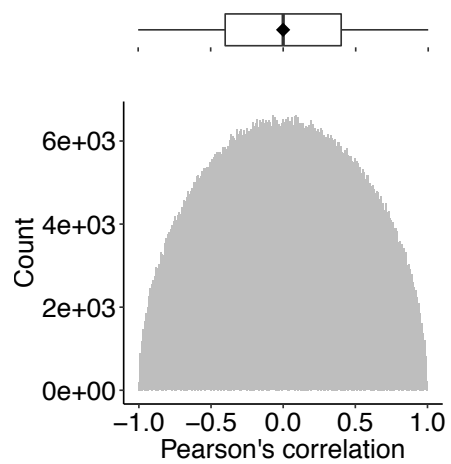

Supplement: S1 Fig — The underlying data can be obtained with the scripts presented in S1 Code. (PDF) [file pbio.3000046.s001.pdf]

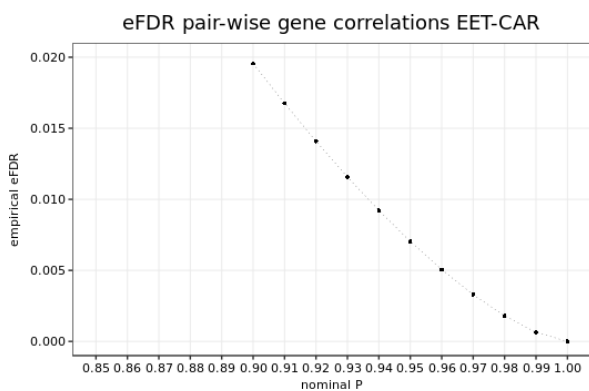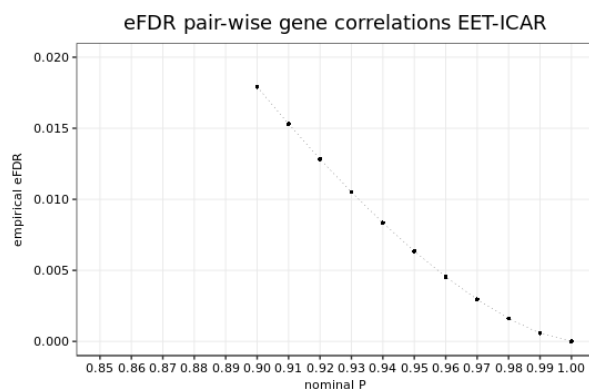

Supplement: S2 Fig — The underlying data can be obtained with the scripts presented in S1 Code. eFDR, empirical false discovery rate (PDF) [file pbio.3000046.s002.pdf]

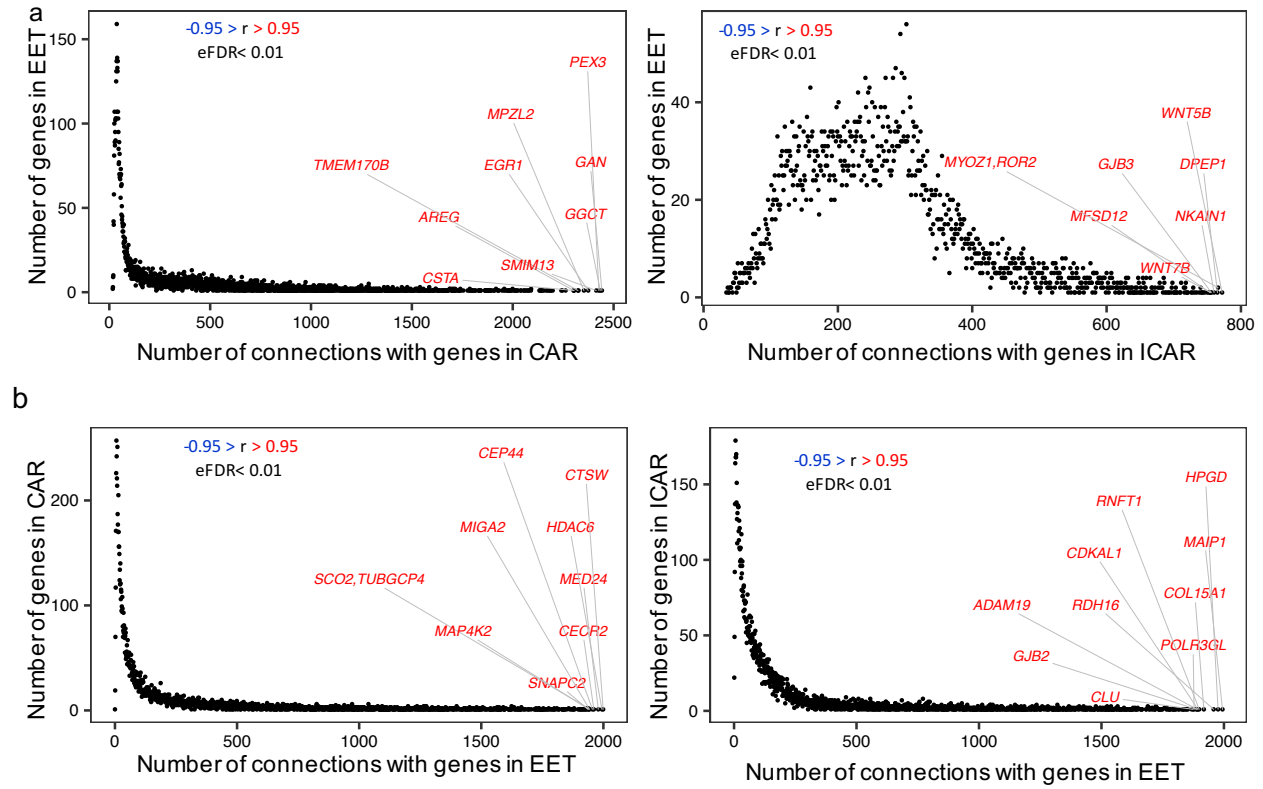

Supplement: S5 Fig — (a) EET and CAR tissues. (b) EET and ICAR tissues. The underlying data can be obtained with the scripts presented in S1 Code. CAR, caruncular; EET, extraembryonic tissue; ICAR, intercaruncular (PDF) [file pbio.3000046.s005.pdf]

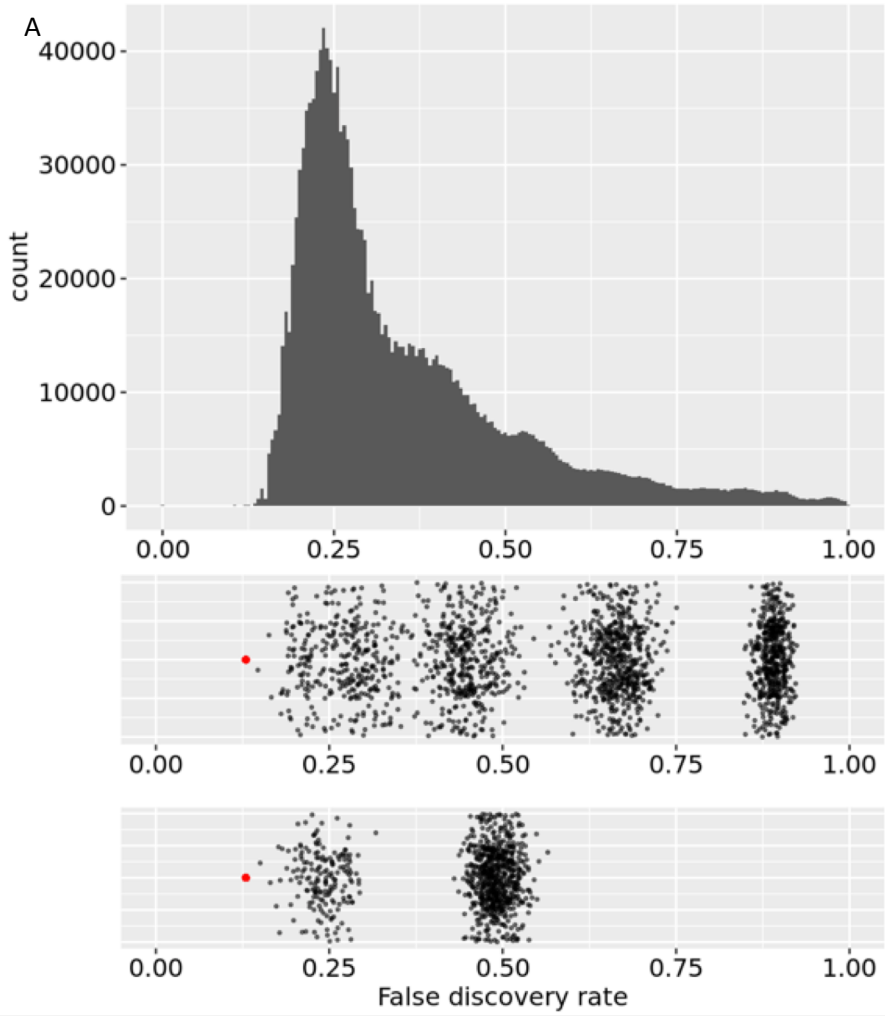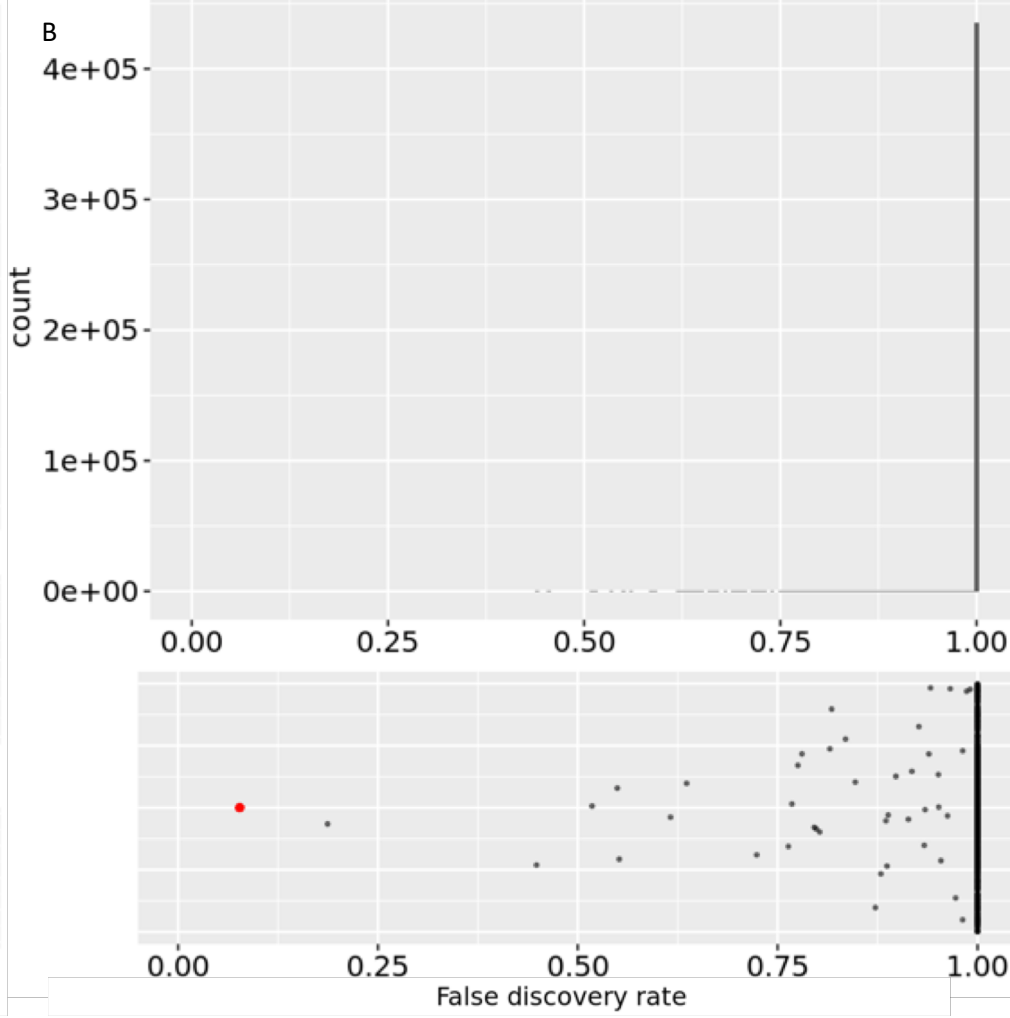

Supplement: S6 Fig — (A) Distribution of false discovery values for all categories of biological process tested. The middle and bottom panels show the distribution of values of false discovery rate for the categories “mRNA processing” (middle) and “chromatin organization” (bottom). (B) Distribution of false discovery values for all KEGG terms tested. The bottom panel show the distribution of values of FDR for the pathway “RNA transport.” Red dot represents the FDR obtained from the real data, and black dots represent FDRs obtained from the genes sampled randomly from the data set. The underlying data can be obtained with the scripts presented in S1 Code. FDR, false discovery rate; GO, gene ontology; KEGG, Kyoto Encyclopedia of Genes and Genomes (PDF) [file pbio.3000046.s006.pdf]

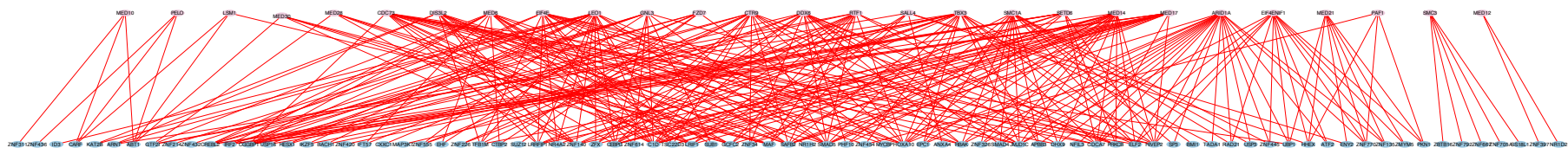

Supplement: S7 Fig — The underlying data can be obtained with the scripts presented in S1 Code. (PDF) [file pbio.3000046.s007.pdf]
