# Supplementary material for: Fine-tuned adaptation of embryo–endometrium pairs at implantation revealed by transcriptome analyses in Bos taurus
Source: PLoS Biol. 2019 Apr 12;17(4):e3000046. doi: 10.1371/journal.pbio.3000046 (PMC6481875; doi:10.1371/journal.pbio.3000046)
Supplement: S3 Fig — The underlying data can be obtained with the scripts presented in S1 Code. CAR, caruncular; EET, extraembryonic tissue (PDF) [file pbio.3000046.s003.pdf]

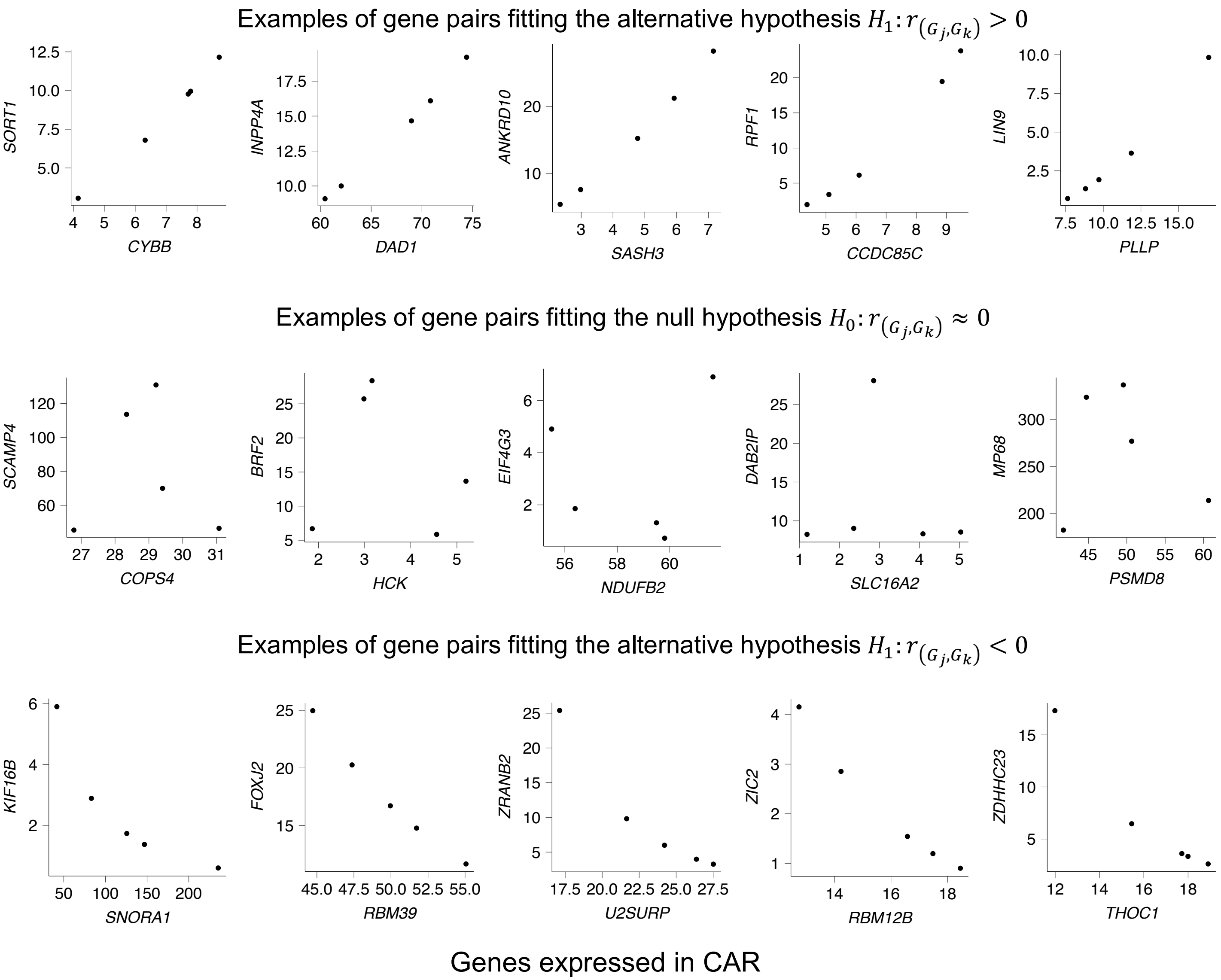
Examples of gene pairs fitting the alternative hypothesis $H_1: r_{(G_j,G_k)} > 0$
SORT1
CYBB
INPP4A
DAD1
ANKRD10
SASH3
RPF1
CCDC85C
LIN9
PLLP
Examples of gene pairs fitting the null hypothesis $H_0: r_{(G_j,G_k)} \approx 0$
SCAMP4
COPS4
BRF2
HCK
EIF4G3
NDUFB2
DAB2IP
SLC16A2
MP68
PSMD8
Examples of gene pairs fitting the alternative hypothesis $H_1: r_{(G_j,G_k)} < 0$
KIF16B
SNORA1
FOXJ2
RBM39
ZRANB2
U2SURP
ZIC2
RBM12B
ZDHHC23
THOC1
Genes expressed in CAR
