# Supplementary material for: Fine-tuned adaptation of embryo–endometrium pairs at implantation revealed by transcriptome analyses in Bos taurus
Source: PLoS Biol. 2019 Apr 12;17(4):e3000046. doi: 10.1371/journal.pbio.3000046 (PMC6481875; doi:10.1371/journal.pbio.3000046)
Supplement: S4 Fig — The underlying data can be obtained with the scripts presented in S1 Code. EET, extraembryonic tissue; ICAR, intercaruncular (PDF) [file pbio.3000046.s004.pdf]

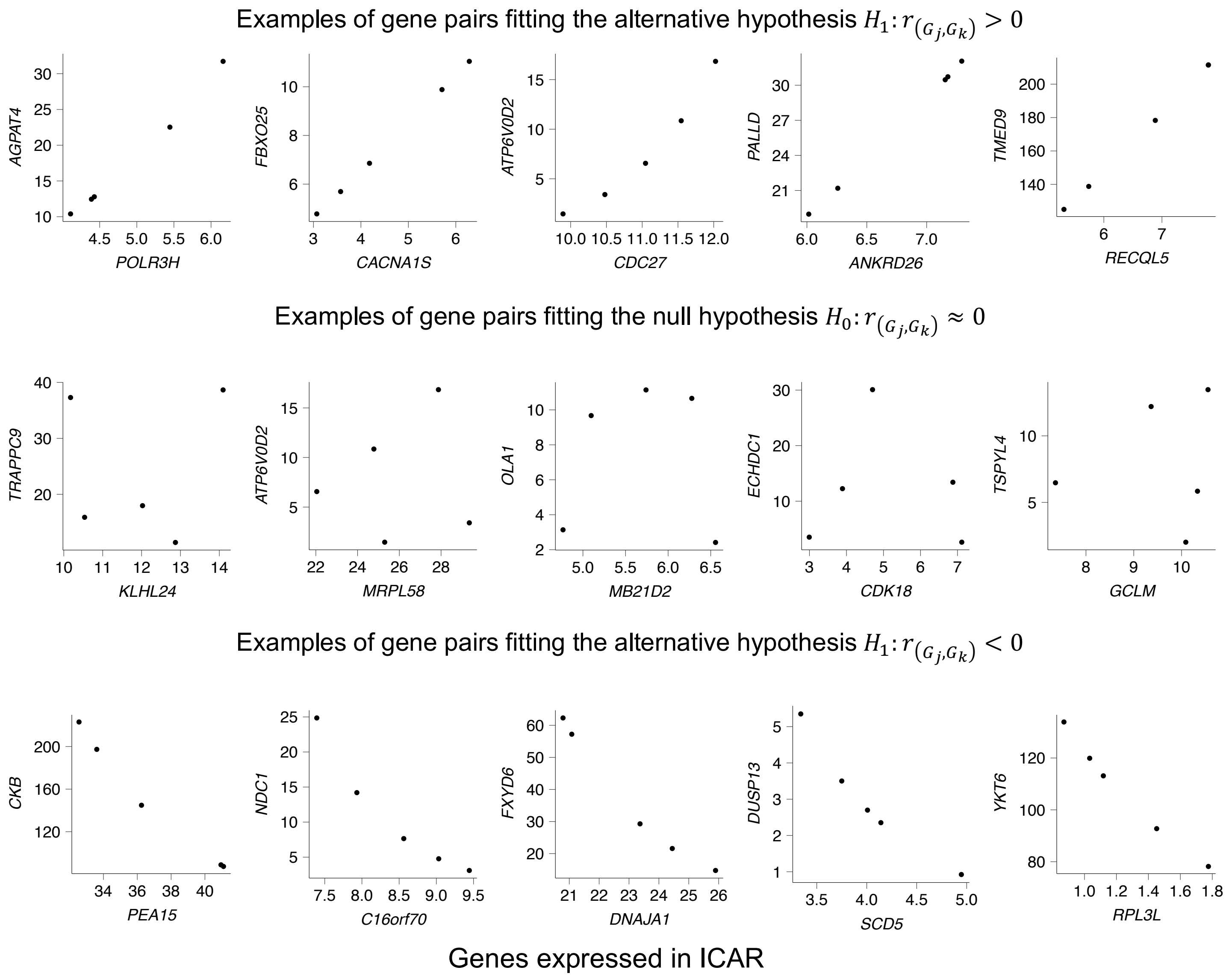

Examples of gene pairs fitting the alternative hypothesis $H_1: r_{(G_j,G_k)} > 0$
AGPAT4
POLR3H
FBXO25
CACNA1S
ATP6V0D2
CDC27
PALLD
ANKRD26
TMED9
RECQL5
Examples of gene pairs fitting the null hypothesis $H_0: r_{(G_j,G_k)} \approx 0$
TRAPPC9
KLHL24
ATP6V0D2
MRPL58
OLA1
MB21D2
ECHDC1
CDK18
TSPYL4
GCLM
Examples of gene pairs fitting the alternative hypothesis $H_1: r_{(G_j,G_k)} < 0$
CKB
PEA15
NDC1
C16orf70
FXYD6
DNAJA1
DUSP13
SCD5
YKT6
RPL3L
Genes expressed in ICAR
