# Supplementary material for: Fine-tuned adaptation of embryo–endometrium pairs at implantation revealed by transcriptome analyses in Bos taurus
Source: PLoS Biol. 2019 Apr 12;17(4):e3000046. doi: 10.1371/journal.pbio.3000046 (PMC6481875; doi:10.1371/journal.pbio.3000046)
Supplement: S1 Table — The underlying data can be obtained with the scripts presented on S1 Code. EET, extraembryonic tissue (PDF) [file pbio.3000046.s008.pdf]

Pearson's r	Genes from paired data EET	CAR	eFDR	Proportion of reoccurrence[a] on scrambled data	Genes from paired data EET	ICAR	eFDR	Proportion of reoccurrence[a] on scrambled data
> 0.9999	180	172	<0.0001	3.6×10^{-10}	106	102	<0.0001	2.1×10^{-10}
> 0.999	3044	2534	<0.0001	8.4×10^{-08}	2714	1589	<0.0001	2.9×10^{-08}
> 0.99	8907	8858	0.0006	7.3×10^{-06}	9526	7081	0.0006	3.9×10^{-06}
> 0.95	9548	12430	0.0070	2.8×10^{-04}	9548	11504	0.0063	1.3×10^{-04}
< -0.95	9548	10411	0.0070	9.4×10^{-05}	9548	10873	0.0063	4.6×10^{-05}
< -0.99	8337	4069	0.0006	1.1×10^{-06}	9499	6535	0.0006	5.3×10^{-07}
< -0.999	1248	685	<0.0001	2.3×10^{-09}	2728	1595	<0.0001	1.2×10^{-09}
< -0.9999	50	46	<0.0001	0	105	102	<0.0001	0

[a] reoccurrence of the same gene pair with correlation in the scrambled data that is greater than positive or lower than negative correlations observed in the conceptus-endometrium paired data.
