# Supplementary material for: Fine-tuned adaptation of embryo–endometrium pairs at implantation revealed by transcriptome analyses in Bos taurus
Source: PLoS Biol. 2019 Apr 12;17(4):e3000046. doi: 10.1371/journal.pbio.3000046 (PMC6481875; doi:10.1371/journal.pbio.3000046)
Supplement: S1 Code — (HTML) [file pbio.3000046.s009.html]

# Supplementary file to Fine-tuned adaptation of embryo-endometrium pairs at implantation revealed by gene regulatory networks in Bos taurus

#### *November/2018*

Please, direct questions to Fernando Biase at *fbiase* at *auburn* dot *edu*. For updated contact, visit www.biaselaboratory.com

# Overview

The originl data used in this study on the NCBI GEO public repository GSE74152

The files utlized as input for our analysis can can be downloaded using the following links:

2017\_06\_17\_fpkm\_d18\_EET\_filtered.txt.bz2   
 2017\_06\_17\_fpkm\_d18\_ENDO\_CAR\_filtered.txt.bz2   
 2017\_06\_17\_fpkm\_d18\_ENDO\_ICAR\_filtered.txt.bz2   
 2017\_12\_20\_annotation.ensembl.symbol.txt.bz2   
 2017\_12\_20\_annotation.genelength.biomart.txt.bz2   
 2017\_12\_20\_annotation.GO.biomart.txt.bz2

Load the libraries needed for the analyses

```
library("WGCNA")
library("ggplot2")
library("reshape")
library("multtest")
library("goseq")
library("flashClust")
library("gplots")
library("foreach")
library("doParallel")
library("bigmemory")
library("gtools")
library("biomaRt")
library("VennDiagram")
library("Rtsne")
library("ComplexHeatmap")
library("circlize")
library("ggrepel")
library("dendextend")
library("edgeR")
library("parallelDist")
library('scales')
library('org.Bt.eg.db')
library('ggpubr')
library('vegan')
library('knitr')
setwd("/data/auburn/eet_endo_interaction/knit/")
```

Load the files contatining expression data

```
fpkm_eet_day_18_AI<-read.table("2017_06_17_fpkm_d18_EET_filtered.txt.bz2",stringsAsFactors=FALSE, header =TRUE)
fpkm_endo_day_18_C_AI<-read.table("2017_06_17_fpkm_d18_ENDO_CAR_filtered.txt.bz2",stringsAsFactors=FALSE, header =TRUE)
fpkm_endo_day_18_IC_AI<-read.table("2017_06_17_fpkm_d18_ENDO_ICAR_filtered.txt.bz2",stringsAsFactors=FALSE, header =TRUE)
```

# Data overview

## Figure 1a

```
ven_diagramm<-venn.diagram(list("EET" = rownames(fpkm_eet_day_18_AI), "CAR" = rownames(fpkm_endo_day_18_C_AI), "ICAR"= rownames(fpkm_endo_day_18_IC_AI)),filename=NULL, fill=c("#CC79A7","#009E73", "#0072B2"), alpha=0.3, euler.d=FALSE,scaled =FALSE,height = 1000, width = 1000,cex=0.9, cat.cex=c(1,1,1),cat.pos=c(0,0,180), lty = rep("blank", 3))
```

```
grid.draw(ven_diagramm)
```

## Figure 1d

```
data.for.tsne<-merge(fpkm_eet_day_18_AI,fpkm_endo_day_18_C_AI,by='row.names', all=TRUE)
data.for.tsne<-merge(data.for.tsne,fpkm_endo_day_18_IC_AI, by.x= "Row.names", by.y="row.names", all=TRUE)
rownames(data.for.tsne)<-data.for.tsne$Row.names
data.for.tsne<-data.for.tsne[,c(2:16)]
data.for.tsne[is.na(data.for.tsne)]<-0
data.for.tsne<-t(log2(data.for.tsne+1))
set.seed(8643)
tsne <- Rtsne(data.for.tsne, dims = 2, theta = 0, perplexity=4, verbose=FALSE, max_iter = 500000)
rm(data.for.tsne)
```

```
group<-factor(c(rep(c("EET", "CAR", "ICAR" ), c(5,5,5))), levels = c("EET", "CAR", "ICAR"))
TSNE<-data.frame("DIM1"=tsne$Y[,1], "DIM2"=tsne$Y[,2], "group"=group )

ggplot(data=TSNE, aes(x=DIM1,y=DIM2) ) + 
  geom_point(aes(colour = factor(group)), size=3 , alpha =0.8 ) +
  scale_color_manual(values= c("#CC79A7", "#009E73","#0072B2") ,name  =NULL) +
  scale_y_continuous(name="t-SNE dim 2")+
  scale_x_continuous(name="t-SNE dim 1")+
  theme_bw(base_size = 20)+
  theme(aspect.ratio =1,
        axis.text=element_blank()
  )
```

# Pair-wise correlation between EET and CAR

```
fpkm_eet_day_18_AI_a<-t(fpkm_eet_day_18_AI)
fpkm_eet_day_18_AI_a<-log2(fpkm_eet_day_18_AI_a+1)

fpkm_endo_day_18_C_AI_a<-t(fpkm_endo_day_18_C_AI)
fpkm_endo_day_18_C_AI_a<-log2(fpkm_endo_day_18_C_AI_a + 1)

pearson_correlation_test<-corAndPvalue(fpkm_eet_day_18_AI_a,fpkm_endo_day_18_C_AI_a,  use = "pairwise.complete.obs", method="pearson",alternative = "two.sided")

pearson_correlation_COR_melt<-melt(pearson_correlation_test$cor)
pearson_correlation_PVALUE_melt<-melt(pearson_correlation_test$p)
pearson_correlation_obs_melt<-melt(pearson_correlation_test$nObs)

correlation_COR_PVALUE_EET_CAR<-pearson_correlation_COR_melt
#correlation_COR_PVALUE_EET_CAR$p_value<-pearson_correlation_PVALUE_melt[,3]
#correlation_COR_PVALUE_EET_CAR$obs<-pearson_correlation_obs_melt[,3]

rm(pearson_correlation_test,pearson_correlation_COR_melt,pearson_correlation_PVALUE_melt,pearson_correlation_obs_melt)

#colnames(correlation_COR_PVALUE_EET_CAR)<-c("gene_id_eet","gene_id_end", "pearson_cor","p_value","obs" )
colnames(correlation_COR_PVALUE_EET_CAR)<-c("gene_id_eet","gene_id_end", "pearson_cor")
```

# Pair-wise correlation between EET and ICAR

```
fpkm_eet_day_18_AI_a<-t(fpkm_eet_day_18_AI)
fpkm_eet_day_18_AI_a<-log2(fpkm_eet_day_18_AI_a+1)

fpkm_endo_day_18_IC_AI_a<-t(fpkm_endo_day_18_IC_AI)
fpkm_endo_day_18_IC_AI_a<-log2(fpkm_endo_day_18_IC_AI_a + 1)

pearson_correlation_test<-corAndPvalue(fpkm_eet_day_18_AI_a,  fpkm_endo_day_18_IC_AI_a,  use = "pairwise.complete.obs", method="pearson",alternative = "two.sided")

pearson_correlation_COR_melt<-melt(pearson_correlation_test$cor)
pearson_correlation_PVALUE_melt<-melt(pearson_correlation_test$p)
pearson_correlation_obs_melt<-melt(pearson_correlation_test$nObs)

correlation_COR_PVALUE_EET_ICAR<-pearson_correlation_COR_melt
#correlation_COR_PVALUE_EET_ICAR$p_value<-pearson_correlation_PVALUE_melt[,3]
#correlation_COR_PVALUE_EET_ICAR$obs<-pearson_correlation_obs_melt[,3]

rm(pearson_correlation_test,pearson_correlation_COR_melt,pearson_correlation_PVALUE_melt,pearson_correlation_obs_melt)

#colnames(correlation_COR_PVALUE_EET_ICAR)<-c("gene_id_eet","gene_id_end", "pearson_cor","p_value","obs" )
colnames(correlation_COR_PVALUE_EET_ICAR)<-c("gene_id_eet","gene_id_end", "pearson_cor" )
```

# eFDR for pair-wise correlation between EET and CAR

```
eet<- as.big.matrix(fpkm_eet_day_18_AI_a , type = "double", 
                    separated = FALSE, 
                    backingfile = "fpkm_eet_day_18_AI_a.bin", 
                    descriptorfile = "fpkm_eet_day_18_AI_a.desc", 
                    share=TRUE)
# get a description of the matrix
mdesc_eet <- describe(eet)

endo <- as.big.matrix(fpkm_endo_day_18_C_AI_a, type = "double", 
                      separated = FALSE, 
                      backingfile = "fpkm_endo_day_18_C_AI_a.bin", 
                      descriptorfile = "fpkm_endo_day_18_C_AI_a.desc",
                      share=TRUE)
# get a description of the matrix
mdesc_end<- describe(endo)

permutation<-permutations(n = 5, r = 5, v = 1:5,repeats.allowed=FALSE)
permutation<-permutation[15:114,]

rand<-dim(permutation)[1]

sequence.correlation<-seq(0.90, 1, 0.01)

results <- filebacked.big.matrix(length(sequence.correlation),rand, type="double", init=0, separated=FALSE, 
                                 backingfile="incidence_matrix.bin",
                                 descriptor="incidence_matrix.desc")
mdesc_result<- describe(results)

cl <- makeCluster(10)
registerDoParallel(cl)

results[,]<-foreach(i = sequence.correlation, .combine='rbind', .inorder=TRUE,  .packages=c("WGCNA","reshape","bigmemory"), .noexport=c("eet", "endo"), .verbose=FALSE) %:%
  
  foreach(j = 1:rand, .combine='cbind', .inorder=FALSE,.packages=c("WGCNA","reshape","bigmemory"), .noexport=c("eet", "endo"), .verbose=FALSE ) %dopar% {
    
    require(bigmemory)
    
    eet<- attach.big.matrix("fpkm_eet_day_18_AI_a.desc")
    endo<- attach.big.matrix("fpkm_endo_day_18_C_AI_a.desc")
    random<-cor(eet[permutation[j,],],  endo[,],  use = "pairwise.complete.obs", method="pearson")
    
    length(which(abs(random) > i))  
    
  }

stopCluster(cl)

#results[1:5,1:5]

system("rm fpkm_eet_day_18_AI_a.bin")
system("rm fpkm_eet_day_18_AI_a.desc")
system("rm fpkm_endo_day_18_C_AI_a.bin")
system("rm fpkm_endo_day_18_C_AI_a.desc")
system("rm incidence_matrix.bin")
system("rm incidence_matrix.desc")

total.rand <- 100 * 124572756

qvalue_CAR<-data.frame(correlation = sequence.correlation, e.pvalue= (rowSums(results[,])+1)/(total.rand+1))
rm(results)
```

# eFDR for pair-wise correlation between EET and ICAR

```
eet<- as.big.matrix(fpkm_eet_day_18_AI_a , type = "double", 
                    separated = FALSE, 
                    backingfile = "fpkm_eet_day_18_AI_a.bin", 
                    descriptorfile = "fpkm_eet_day_18_AI_a.desc", 
                    share=TRUE)
# get a description of the matrix
mdesc_eet <- describe(eet)

endo <- as.big.matrix(fpkm_endo_day_18_IC_AI_a, type = "double", 
                      separated = FALSE, 
                      backingfile = "fpkm_endo_day_18_IC_AI_a.bin", 
                      descriptorfile = "fpkm_endo_day_18_IC_AI_a.desc",
                      share=TRUE)
# get a description of the matrix
mdesc_end<- describe(endo)

permutation<-permutations(n = 5, r = 5, v = 1:5,repeats.allowed=FALSE)
permutation<-permutation[15:114,]

rand<-dim(permutation)[1]

sequence.correlation<-seq(0.90, 1, 0.01)

results <- filebacked.big.matrix(length(sequence.correlation),rand, type="double", init=0, separated=FALSE, 
                                 backingfile="incidence_matrix.bin",
                                 descriptor="incidence_matrix.desc")
mdesc_result<- describe(results)

cl <- makeCluster(10)
registerDoParallel(cl)

results[,]<-foreach(i = sequence.correlation, .combine='rbind', .inorder=TRUE,  .packages=c("WGCNA","reshape","bigmemory"), .noexport=c("eet", "endo"), .verbose=FALSE) %:%
  
  foreach(j = 1:rand, .combine='cbind', .inorder=FALSE,.packages=c("WGCNA","reshape","bigmemory"), .noexport=c("eet", "endo"), .verbose=FALSE ) %dopar% {
    
    require(bigmemory)
    
    eet<- attach.big.matrix("fpkm_eet_day_18_AI_a.desc")
    endo<- attach.big.matrix("fpkm_endo_day_18_IC_AI_a.desc")
    random<-cor(eet[permutation[j,],],  endo[,],  use = "pairwise.complete.obs", method="pearson")
    
    length(which(abs(random) > i))  
    
  }

stopCluster(cl)

system("rm fpkm_eet_day_18_AI_a.bin")
system("rm fpkm_eet_day_18_AI_a.desc")
system("rm fpkm_endo_day_18_IC_AI_a.bin")
system("rm fpkm_endo_day_18_IC_AI_a.desc")
system("rm incidence_matrix.bin")
system("rm incidence_matrix.desc")

total.rand <- 100 * 124610948

qvalue_ICAR<-data.frame(correlation = sequence.correlation, e.pvalue= (rowSums(results[,])+1)/(total.rand+1))
```

## Figure 2a

```
correlation_COR_PVALUE_EET_CAR_cor_above09<-correlation_COR_PVALUE_EET_CAR[correlation_COR_PVALUE_EET_CAR$pearson_cor > 0.95, ]
correlation_COR_PVALUE_EET_CAR_cor_below_neg_09<-correlation_COR_PVALUE_EET_CAR[correlation_COR_PVALUE_EET_CAR$pearson_cor < -0.95, ]

plot1<-ggplot(data=correlation_COR_PVALUE_EET_CAR, aes(y=pearson_cor, x=1)) +
  geom_boxplot()+
  stat_summary(fun.y=mean, geom="point", shape=18, size=4, fill='black')+
  coord_flip()+
  theme(
  panel.background = element_blank(),
  panel.grid.major = element_blank(),
  panel.grid.minor = element_blank(),
  plot.background = element_blank(),
  axis.ticks.y = element_blank(),
  axis.text = element_blank(),
  axis.title = element_blank(),
  plot.margin= unit(c(0,0,0,0), "cm")
  )
  

plot2<- ggplot() + 
  geom_histogram(aes(x=pearson_cor), fill="gray",  data=correlation_COR_PVALUE_EET_CAR, binwidth = 0.01) + 
  geom_histogram(aes(x=pearson_cor), fill="red",  data=correlation_COR_PVALUE_EET_CAR_cor_above09, binwidth = 0.01) +
  geom_histogram(aes(x=pearson_cor), fill="blue",  data=correlation_COR_PVALUE_EET_CAR_cor_below_neg_09, binwidth = 0.01)  + 
  scale_y_continuous("Count",labels = function(x) format(x, scientific = TRUE))+
  scale_x_continuous("Pearson's correlation")+
    theme(
    panel.background = element_blank(),
    panel.grid.major = element_blank(),
    panel.grid.minor = element_blank(),
    plot.background = element_blank(),
    axis.line = element_line(colour = "black"),
    axis.text = element_text(colour="black", size =17),
    axis.title = element_text(colour="black", size =17),
    plot.margin= unit(c(0,0.4,0,0), "cm")
  )
  
ggarrange(plot1, plot2, ncol=1, nrow=2, heights=c(1,4), align="hv")
```

## Figure 2b

```
correlation_COR_PVALUE_EET_ICAR_cor_above09<-correlation_COR_PVALUE_EET_ICAR[correlation_COR_PVALUE_EET_ICAR$pearson_cor > 0.95, ]
correlation_COR_PVALUE_EET_ICAR_cor_below_neg_09<-correlation_COR_PVALUE_EET_ICAR[correlation_COR_PVALUE_EET_ICAR$pearson_cor < -0.95, ]

plot1<-ggplot(data=correlation_COR_PVALUE_EET_ICAR, aes(y=pearson_cor, x=1)) +
  geom_boxplot()+
  stat_summary(fun.y=mean, geom="point", shape=18, size=4, fill='black')+
  coord_flip()+
  theme(
    panel.background = element_blank(),
    panel.grid.major = element_blank(),
    panel.grid.minor = element_blank(),
    plot.background = element_blank(),
    axis.ticks.y = element_blank(),
    axis.text = element_blank(),
    axis.title = element_blank(),
    plot.margin= unit(c(0,0,0,0), "cm")
  )


plot2<- ggplot() + 
  geom_histogram(aes(x=pearson_cor), fill="gray",  data=correlation_COR_PVALUE_EET_ICAR, binwidth = 0.01) + 
  geom_histogram(aes(x=pearson_cor), fill="red",  data=correlation_COR_PVALUE_EET_ICAR_cor_above09, binwidth = 0.01) +
  geom_histogram(aes(x=pearson_cor), fill="blue",  data=correlation_COR_PVALUE_EET_ICAR_cor_below_neg_09, binwidth = 0.01)  + 
  scale_y_continuous("Count",labels = function(x) format(x, scientific = TRUE))+
  scale_x_continuous("Pearson's correlation")+
  theme(
    panel.background = element_blank(),
    panel.grid.major = element_blank(),
    panel.grid.minor = element_blank(),
    plot.background = element_blank(),
    axis.line = element_line(colour = "black"),
    axis.text = element_text(colour="black", size =17),
    axis.title = element_text(colour="black", size =17),
    plot.margin= unit(c(0,0.4,0,0), "cm")
  )

ggarrange(plot1, plot2, ncol=1, nrow=2, heights=c(1,4), align="hv")
```

### Supplemental figure S1

```
permutation<-permutations(n = 5, r = 5, v = 1:5,repeats.allowed=FALSE)
permutation<-permutation[10:110,]
permutation<-permutation[sample(101,10,replace = FALSE),]

#CAR

i=1
suffled_data_EET_CAR_melt_a<-melt(cor(fpkm_eet_day_18_AI_a[permutation[i,],],  fpkm_endo_day_18_C_AI_a,  use = "pairwise.complete.obs"))

for (i in c(2:10)){
  
  suffled_data_EET_CAR<-cor(fpkm_eet_day_18_AI_a[permutation[i,],],  fpkm_endo_day_18_C_AI_a,  use = "pairwise.complete.obs")
  suffled_data_EET_CAR_melt<-melt(suffled_data_EET_CAR)
  suffled_data_EET_CAR_melt_a[,(2+i)]<-round(suffled_data_EET_CAR_melt$value ,2)
  
}

sample_suffled_data_EET_CAR_melt_a<-data.frame("value"=sample(as.matrix(suffled_data_EET_CAR_melt_a[,c(3:12)]), 1000000, replace=TRUE))

plot1<-ggplot(data=sample_suffled_data_EET_CAR_melt_a, aes(y=value, x=1)) +
  geom_boxplot()+
  stat_summary(fun.y=mean, geom="point", shape=18, size=4, fill='black')+
  coord_flip()+
  ggtitle("Distribution of correlations between \n EET and CAR under the null hypothesis")+
  theme(
    panel.background = element_blank(),
    panel.grid.major = element_blank(),
    panel.grid.minor = element_blank(),
    plot.background = element_blank(),
    axis.ticks.y = element_blank(),
    axis.text = element_blank(),
    axis.title = element_blank(),
    plot.margin= unit(c(0,0,0,0), "cm"),
    plot.title = element_text(color="black", size=12,hjust = 0.5)
  )

plot2<- ggplot() + 
  geom_histogram(aes(x=value), fill="gray",  data=sample_suffled_data_EET_CAR_melt_a, binwidth = 0.01) + 
  #geom_histogram(aes(x=value), fill="red",  data=sample_suffled_data_EET_CAR_melt_a_above09, binwidth = 0.01) +
  #geom_histogram(aes(x=value), fill="blue",  data=sample_suffled_data_EET_CAR_melt_a_below_neg_09, binwidth = 0.01)  + 
  scale_y_continuous("Count",labels = function(x) format(x, scientific = TRUE))+
  scale_x_continuous("Pearson's correlation")+
  theme(
    panel.background = element_blank(),
    panel.grid.major = element_blank(),
    panel.grid.minor = element_blank(),
    plot.background = element_blank(),
    axis.line = element_line(colour = "black"),
    axis.text = element_text(colour="black", size =17),
    axis.title = element_text(colour="black", size =17),
    plot.margin= unit(c(0,0.4,0,0), "cm")
  )
  

#ICAR

i=1
suffled_data_EET_ICAR_melt_a<-melt(cor(fpkm_eet_day_18_AI_a[permutation[i,],],  fpkm_endo_day_18_IC_AI_a,  use = "pairwise.complete.obs"))

for (i in c(2:10)){
  
  suffled_data_EET_ICAR<-cor(fpkm_eet_day_18_AI_a[permutation[i,],],  fpkm_endo_day_18_IC_AI_a,  use = "pairwise.complete.obs")
  suffled_data_EET_ICAR_melt<-melt(suffled_data_EET_ICAR)
  suffled_data_EET_ICAR_melt_a[,(2+i)]<-round(suffled_data_EET_ICAR_melt$value ,2)
  
}

sample_suffled_data_EET_ICAR_melt_a<-data.frame("value"=sample(as.matrix(suffled_data_EET_ICAR_melt_a[,c(3:12)]), 1000000, replace=TRUE))


plot3<-ggplot(data=sample_suffled_data_EET_ICAR_melt_a, aes(y=value, x=1)) +
  geom_boxplot()+
  stat_summary(fun.y=mean, geom="point", shape=18, size=4, fill='black')+
  coord_flip()+
  ggtitle("Distribution of correlations between \n EET and ICAR under the null hypothesis")+
  theme(
    panel.background = element_blank(),
    panel.grid.major = element_blank(),
    panel.grid.minor = element_blank(),
    plot.background = element_blank(),
    axis.ticks.y = element_blank(),
    axis.text = element_blank(),
    axis.title = element_blank(),
    plot.margin= unit(c(0,0,0,0), "cm"),
    plot.title = element_text(color="black", size=12,hjust = 0.5)
  )

plot4<- ggplot() + 
  geom_histogram(aes(x=value), fill="gray",  data=sample_suffled_data_EET_ICAR_melt_a, binwidth = 0.01) + 
  #geom_histogram(aes(x=value), fill="red",  data=sample_suffled_data_EET_ICAR_melt_a_above09, binwidth = 0.01) +
  #geom_histogram(aes(x=value), fill="blue",  data=sample_suffled_data_EET_ICAR_melt_a_below_neg_09, binwidth = 0.01)  + 
  scale_y_continuous("Count",labels = function(x) format(x, scientific = TRUE))+
  scale_x_continuous("Pearson's correlation")+
  theme(
    panel.background = element_blank(),
    panel.grid.major = element_blank(),
    panel.grid.minor = element_blank(),
    plot.background = element_blank(),
    axis.line = element_line(colour = "black"),
    axis.text = element_text(colour="black", size =17),
    axis.title = element_text(colour="black", size =17),
    plot.margin= unit(c(0,0.4,0,0), "cm")
  )

ggarrange(plot1, plot3,plot2, plot4, ncol=2, nrow=2, heights=c(1.5,5), align="hv")
```

Testing the deviation of the null versus the the alternative correlations EET - CAR

```
ks.test(sample_suffled_data_EET_CAR_melt_a$value,correlation_COR_PVALUE_EET_CAR$pearson_cor)
```

```
## 
##  Two-sample Kolmogorov-Smirnov test
## 
## data:  sample_suffled_data_EET_CAR_melt_a$value and correlation_COR_PVALUE_EET_CAR$pearson_cor
## D = 0.13942, p-value < 2.2e-16
## alternative hypothesis: two-sided
```

Testing the deviation of the null versus the the alternative correlations EET - ICAR

```
ks.test(sample_suffled_data_EET_ICAR_melt_a$value,correlation_COR_PVALUE_EET_ICAR$pearson_cor)
```

```
## 
##  Two-sample Kolmogorov-Smirnov test
## 
## data:  sample_suffled_data_EET_ICAR_melt_a$value and correlation_COR_PVALUE_EET_ICAR$pearson_cor
## D = 0.059979, p-value < 2.2e-16
## alternative hypothesis: two-sided
```

### Supplemental figure S2

```
#qvalue_ICAR<-read.table("2017-12-19_empirical_p_ICAR_rand.txt",stringsAsFactors=FALSE, header =TRUE, sep="\t",quote = "")
#qvalue_CAR<-read.table("2017-12-18_empirical_p_CAR_rand.txt",stringsAsFactors=FALSE, header =TRUE, sep="\t",quote = "")

plot1<-ggplot()+
  geom_point(data=qvalue_CAR, aes(x=correlation , y=e.pvalue/2), color="black", size=1, shape=16)+
  geom_line(data=qvalue_CAR, aes(x=correlation , y=e.pvalue/2), color="black", size=0.1,linetype=3)+
  scale_x_continuous(name="correlation", limits = c(0.85, 1), breaks=seq(0.85,1, 0.01))+
  scale_y_continuous(name="empirical eFDR", limits = c(0, 0.02), breaks=seq(0,0.2, 0.005))+
  ggtitle("eFDR pair-wise gene correlations EET-CAR")+
  theme_bw()+
  theme(panel.grid= element_blank(),
        panel.background = element_blank(),
        panel.grid.minor = element_blank(),
        panel.grid.major = element_line(color="lightgray"),
        plot.background = element_blank(),
        axis.title=element_text(color="black", size=8),
        axis.text=element_text(color="black", size=8),
        panel.spacing = unit(c(0.4,0.4,0.4,0.4),"cm"),
        plot.margin = unit(c(0.5,0.5,0.5,0.5),"cm"),
        legend.position="none",
        plot.title = element_text(lineheight=.8, hjust=0.5))

plot2<-ggplot()+
  geom_point(data=qvalue_ICAR, aes(x=correlation , y=e.pvalue/2), color="black", size=1, shape=16)+
  geom_line(data=qvalue_ICAR, aes(x=correlation , y=e.pvalue/2), color="black", size=0.1,linetype=3)+
  scale_x_continuous(name="correlation", limits = c(0.85, 1), breaks=seq(0.85,1, 0.01))+
  scale_y_continuous(name="empirical eFDR", limits = c(0, 0.02), breaks=seq(0,0.2, 0.005))+
  ggtitle("eFDR pair-wise gene correlations EET-ICAR")+
  theme_bw()+
  theme(panel.grid= element_blank(),
        panel.background = element_blank(),
        panel.grid.minor = element_blank(),
        panel.grid.major = element_line(color="lightgray"),
        plot.background = element_blank(),
        axis.title=element_text(color="black", size=8),
        axis.text=element_text(color="black", size=8),
        panel.spacing = unit(c(0.4,0.4,0.4,0.4),"cm"),
        plot.margin = unit(c(0.5,0.5,0.5,0.5),"cm"),
        legend.position="none",
        plot.title = element_text(lineheight=.8, hjust=0.5))

ggarrange(plot1, plot2, ncol=2, nrow=1)
```

## Figure 2c

The results presented below were used to produce Figure 2c.

```
merged_correlation<-merge(correlation_COR_PVALUE_EET_CAR[,c(1:3)], correlation_COR_PVALUE_EET_ICAR[,c(1:3)], by=c('gene_id_eet','gene_id_end'),all=TRUE, suffixes=c('.CAR','.ICAR') )
merged_correlation[is.na(merged_correlation)]<-0

nrow(merged_correlation[merged_correlation$pearson_cor.CAR  >  0.99 & merged_correlation$pearson_cor.ICAR > 0.99,])
```

```
## [1] 135
```

```
length(na.omit(unique(merged_correlation[merged_correlation$pearson_cor.CAR  >  0.99 & merged_correlation$pearson_cor.ICAR > 0.99,1])))
```

```
## [1] 132
```

```
length(na.omit(unique(merged_correlation[merged_correlation$pearson_cor.CAR  >  0.99 & merged_correlation$pearson_cor.ICAR > 0.99,2])))
```

```
## [1] 20
```

```
nrow(merged_correlation[merged_correlation$pearson_cor.CAR  <  -0.99 & merged_correlation$pearson_cor.ICAR < -0.99,])
```

```
## [1] 41
```

```
length(na.omit(unique(merged_correlation[merged_correlation$pearson_cor.CAR  <  -0.99 & merged_correlation$pearson_cor.ICAR <  -0.99,1])))
```

```
## [1] 41
```

```
length(na.omit(unique(merged_correlation[merged_correlation$pearson_cor.CAR  <  -0.99 & merged_correlation$pearson_cor.ICAR <  -0.99,2])))
```

```
## [1] 9
```

```
nrow(merged_correlation[merged_correlation$pearson_cor.CAR  >  0.99 & merged_correlation$pearson_cor.ICAR < -0.99,])
```

```
## [1] 94
```

```
length(na.omit(unique(merged_correlation[merged_correlation$pearson_cor.CAR  >  0.99 & merged_correlation$pearson_cor.ICAR < -0.99,1])))
```

```
## [1] 87
```

```
length(na.omit(unique(merged_correlation[merged_correlation$pearson_cor.CAR  >  0.99 & merged_correlation$pearson_cor.ICAR < -0.99,2])))
```

```
## [1] 24
```

```
nrow(merged_correlation[merged_correlation$pearson_cor.CAR  < -0.99  & merged_correlation$pearson_cor.ICAR >  0.99,])
```

```
## [1] 84
```

```
length(na.omit(unique(merged_correlation[merged_correlation$pearson_cor.CAR  < -0.99  & merged_correlation$pearson_cor.ICAR >  0.99,1])))
```

```
## [1] 81
```

```
length(na.omit(unique(merged_correlation[merged_correlation$pearson_cor.CAR  < -0.99  & merged_correlation$pearson_cor.ICAR >  0.99,2])))
```

```
## [1] 14
```

```
nrow(merged_correlation[merged_correlation$pearson_cor.CAR  >  0.99 ,])
```

```
## [1] 207189
```

```
length(na.omit(unique(merged_correlation[merged_correlation$pearson_cor.CAR  >  0.99 ,1])))
```

```
## [1] 8907
```

```
length(na.omit(unique(merged_correlation[merged_correlation$pearson_cor.CAR  >  0.99 ,2])))
```

```
## [1] 8858
```

```
nrow(merged_correlation[merged_correlation$pearson_cor.CAR  <  -0.99 ,])
```

```
## [1] 49967
```

```
length(na.omit(unique(merged_correlation[merged_correlation$pearson_cor.CAR  <  -0.99 ,1])))
```

```
## [1] 8337
```

```
length(na.omit(unique(merged_correlation[merged_correlation$pearson_cor.CAR  <  -0.99 ,2])))
```

```
## [1] 4069
```

```
nrow(merged_correlation[merged_correlation$pearson_cor.ICAR  >  0.99 ,])
```

```
## [1] 107421
```

```
length(na.omit(unique(merged_correlation[merged_correlation$pearson_cor.ICAR  >  0.99 ,1])))
```

```
## [1] 9526
```

```
length(na.omit(unique(merged_correlation[merged_correlation$pearson_cor.ICAR  >  0.99 ,2])))
```

```
## [1] 7081
```

```
nrow(merged_correlation[merged_correlation$pearson_cor.ICAR  <  -0.99 ,])
```

```
## [1] 119549
```

```
length(na.omit(unique(merged_correlation[merged_correlation$pearson_cor.ICAR  <  -0.99 ,1])))
```

```
## [1] 9499
```

```
length(na.omit(unique(merged_correlation[merged_correlation$pearson_cor.ICAR  <  -0.99 ,2])))
```

```
## [1] 6535
```

```
rm(merged_correlation)
```

### Supplemental Table 1

This is the code used to obtain the results used to create S1 Table

Calculation of the number of genes according to each treshold

```
length(unique(correlation_COR_PVALUE_EET_CAR[correlation_COR_PVALUE_EET_CAR$pearson_cor>0.9999,1]))
```

```
## [1] 180
```

```
length(unique(correlation_COR_PVALUE_EET_CAR[correlation_COR_PVALUE_EET_CAR$pearson_cor>0.999,1]))
```

```
## [1] 3044
```

```
length(unique(correlation_COR_PVALUE_EET_CAR[correlation_COR_PVALUE_EET_CAR$pearson_cor>0.99,1]))
```

```
## [1] 8907
```

```
length(unique(correlation_COR_PVALUE_EET_CAR[correlation_COR_PVALUE_EET_CAR$pearson_cor>0.95,1]))
```

```
## [1] 9548
```

```
length(unique(correlation_COR_PVALUE_EET_CAR[correlation_COR_PVALUE_EET_CAR$pearson_cor>0.90,1]))
```

```
## [1] 9548
```

```
length(unique(correlation_COR_PVALUE_EET_CAR[correlation_COR_PVALUE_EET_CAR$pearson_cor< -0.90,1]))
```

```
## [1] 9548
```

```
length(unique(correlation_COR_PVALUE_EET_CAR[correlation_COR_PVALUE_EET_CAR$pearson_cor< -0.95,1]))
```

```
## [1] 9548
```

```
length(unique(correlation_COR_PVALUE_EET_CAR[correlation_COR_PVALUE_EET_CAR$pearson_cor< -0.99,1]))
```

```
## [1] 8337
```

```
length(unique(correlation_COR_PVALUE_EET_CAR[correlation_COR_PVALUE_EET_CAR$pearson_cor< -0.999,1]))
```

```
## [1] 1248
```

```
length(unique(correlation_COR_PVALUE_EET_CAR[correlation_COR_PVALUE_EET_CAR$pearson_cor< -0.9999,1]))
```

```
## [1] 50
```

```
length(unique(correlation_COR_PVALUE_EET_CAR[correlation_COR_PVALUE_EET_CAR$pearson_cor>0.9999,2]))
```

```
## [1] 172
```

```
length(unique(correlation_COR_PVALUE_EET_CAR[correlation_COR_PVALUE_EET_CAR$pearson_cor>0.999,2]))
```

```
## [1] 2534
```

```
length(unique(correlation_COR_PVALUE_EET_CAR[correlation_COR_PVALUE_EET_CAR$pearson_cor>0.99,2]))
```

```
## [1] 8858
```

```
length(unique(correlation_COR_PVALUE_EET_CAR[correlation_COR_PVALUE_EET_CAR$pearson_cor>0.95,2]))
```

```
## [1] 12430
```

```
length(unique(correlation_COR_PVALUE_EET_CAR[correlation_COR_PVALUE_EET_CAR$pearson_cor>0.90,2]))
```

```
## [1] 12959
```

```
length(unique(correlation_COR_PVALUE_EET_CAR[correlation_COR_PVALUE_EET_CAR$pearson_cor< -0.90,2]))
```

```
## [1] 12641
```

```
length(unique(correlation_COR_PVALUE_EET_CAR[correlation_COR_PVALUE_EET_CAR$pearson_cor< -0.95,2]))
```

```
## [1] 10411
```

```
length(unique(correlation_COR_PVALUE_EET_CAR[correlation_COR_PVALUE_EET_CAR$pearson_cor< -0.99,2]))
```

```
## [1] 4069
```

```
length(unique(correlation_COR_PVALUE_EET_CAR[correlation_COR_PVALUE_EET_CAR$pearson_cor< -0.999,2]))
```

```
## [1] 685
```

```
length(unique(correlation_COR_PVALUE_EET_CAR[correlation_COR_PVALUE_EET_CAR$pearson_cor< -0.9999,2]))
```

```
## [1] 46
```

```
length(unique(correlation_COR_PVALUE_EET_ICAR[correlation_COR_PVALUE_EET_ICAR$pearson_cor>0.9999,1]))
```

```
## [1] 106
```

```
length(unique(correlation_COR_PVALUE_EET_ICAR[correlation_COR_PVALUE_EET_ICAR$pearson_cor>0.999,1]))
```

```
## [1] 2714
```

```
length(unique(correlation_COR_PVALUE_EET_ICAR[correlation_COR_PVALUE_EET_ICAR$pearson_cor>0.99,1]))
```

```
## [1] 9526
```

```
length(unique(correlation_COR_PVALUE_EET_ICAR[correlation_COR_PVALUE_EET_ICAR$pearson_cor>0.95,1]))
```

```
## [1] 9548
```

```
length(unique(correlation_COR_PVALUE_EET_ICAR[correlation_COR_PVALUE_EET_ICAR$pearson_cor>0.90,1]))
```

```
## [1] 9548
```

```
length(unique(correlation_COR_PVALUE_EET_ICAR[correlation_COR_PVALUE_EET_ICAR$pearson_cor< -0.90,1]))
```

```
## [1] 9548
```

```
length(unique(correlation_COR_PVALUE_EET_ICAR[correlation_COR_PVALUE_EET_ICAR$pearson_cor< -0.95,1]))
```

```
## [1] 9548
```

```
length(unique(correlation_COR_PVALUE_EET_ICAR[correlation_COR_PVALUE_EET_ICAR$pearson_cor< -0.99,1]))
```

```
## [1] 9499
```

```
length(unique(correlation_COR_PVALUE_EET_ICAR[correlation_COR_PVALUE_EET_ICAR$pearson_cor< -0.999,1]))
```

```
## [1] 2728
```

```
length(unique(correlation_COR_PVALUE_EET_ICAR[correlation_COR_PVALUE_EET_ICAR$pearson_cor< -0.9999,1]))
```

```
## [1] 105
```

```
length(unique(correlation_COR_PVALUE_EET_ICAR[correlation_COR_PVALUE_EET_ICAR$pearson_cor>0.9999,2]))
```

```
## [1] 102
```

```
length(unique(correlation_COR_PVALUE_EET_ICAR[correlation_COR_PVALUE_EET_ICAR$pearson_cor>0.999,2]))
```

```
## [1] 1589
```

```
length(unique(correlation_COR_PVALUE_EET_ICAR[correlation_COR_PVALUE_EET_ICAR$pearson_cor>0.99,2]))
```

```
## [1] 7081
```

```
length(unique(correlation_COR_PVALUE_EET_ICAR[correlation_COR_PVALUE_EET_ICAR$pearson_cor>0.95,2]))
```

```
## [1] 11504
```

```
length(unique(correlation_COR_PVALUE_EET_ICAR[correlation_COR_PVALUE_EET_ICAR$pearson_cor>0.90,2]))
```

```
## [1] 12612
```

```
length(unique(correlation_COR_PVALUE_EET_ICAR[correlation_COR_PVALUE_EET_ICAR$pearson_cor< -0.90,2]))
```

```
## [1] 12410
```

```
length(unique(correlation_COR_PVALUE_EET_ICAR[correlation_COR_PVALUE_EET_ICAR$pearson_cor< -0.95,2]))
```

```
## [1] 10873
```

```
length(unique(correlation_COR_PVALUE_EET_ICAR[correlation_COR_PVALUE_EET_ICAR$pearson_cor< -0.99,2]))
```

```
## [1] 6535
```

```
length(unique(correlation_COR_PVALUE_EET_ICAR[correlation_COR_PVALUE_EET_ICAR$pearson_cor< -0.999,2]))
```

```
## [1] 1595
```

```
length(unique(correlation_COR_PVALUE_EET_ICAR[correlation_COR_PVALUE_EET_ICAR$pearson_cor< -0.9999,2]))
```

```
## [1] 102
```

Obtain the proportion of pairs that showed correlation on the scrambled data greater or lower than the trenshold.

```
rm(plot1, plot2, plot3, plot4,suffled_data_EET_ICAR,suffled_data_EET_CAR,suffled_data_EET_CAR_melt_a, suffled_data_EET_ICAR_melt_a, sample_suffled_data_EET_CAR_melt_a, sample_suffled_data_EET_ICAR_melt_a,correlation_COR_PVALUE_EET_ICAR_cor_above09,correlation_COR_PVALUE_EET_ICAR_cor_below_neg_09,
correlation_COR_PVALUE_EET_CAR_cor_above09,correlation_COR_PVALUE_EET_CAR_cor_below_neg_09,tsne,TSNE,ven_diagramm,eet,endo,suffled_data_EET_CAR_melt,suffled_data_EET_ICAR_melt,mdesc_eet,mdesc_end,results,mdesc_result,qvalue_ICAR,qvalue_CAR,fpkm_eet_day_18_AI,fpkm_endo_day_18_IC_AI,fpkm_endo_day_18_C_AI)

gc()
```

```
##             used   (Mb) gc trigger    (Mb)   max used    (Mb)
## Ncells   7829710  418.2  196694636 10504.7  272015474 14527.2
## Vcells 645954902 4928.3 7799221088 59503.4 9749026361 74379.2
```

```
eet<- as.big.matrix(fpkm_eet_day_18_AI_a , type = "double", 
                    separated = FALSE, 
                    backingfile = "fpkm_eet_day_18_AI_a.bin", 
                    descriptorfile = "fpkm_eet_day_18_AI_a.desc", 
                    share=TRUE)
# get a description of the matrix
mdesc_eet <- describe(eet)

endo <- as.big.matrix(fpkm_endo_day_18_C_AI_a, type = "double", 
                      separated = FALSE, 
                      backingfile = "fpkm_endo_day_18_C_AI_a.bin", 
                      descriptorfile = "fpkm_endo_day_18_C_AI_a.desc",
                      share=TRUE)
# get a description of the matrix
mdesc_end<- describe(endo)

permutation<-permutations(n = 5, r = 5, v = 1:5,repeats.allowed=FALSE)

permutation<-permutation[c(4:24,26:48,50:95,97:114,117:118),]

rand<-dim(permutation)[1]

sequence.correlation<-c(0.95,0.99,0.999,0.9999)

cl <- makeCluster(10)
registerDoParallel(cl)

results_eet_car_scrambled<-data.frame()
results_eet_car_scrambled<- foreach(i = sequence.correlation, .combine='rbind', .inorder=FALSE,  .packages=c("reshape","bigmemory"), .noexport=c("eet", "endo"), .verbose=FALSE) %:%

  foreach(j = 1:rand, .combine='rbind', .inorder=FALSE,.packages=c("reshape","bigmemory"), .noexport=c("eet", "endo"), .verbose=FALSE ) %dopar% {
    
    correlation_COR_PVALUE_EET_CAR_subset<-correlation_COR_PVALUE_EET_CAR[correlation_COR_PVALUE_EET_CAR$pearson_cor > i,]
    
    eet<- attach.big.matrix("fpkm_eet_day_18_AI_a.desc")
    endo<- attach.big.matrix("fpkm_endo_day_18_C_AI_a.desc")
    random<-cor(eet[permutation[j,],],  endo[,],  use = "pairwise.complete.obs", method="pearson")
    random<-melt(random)
    random<-random[random$value > i,]
    random<-merge(random,correlation_COR_PVALUE_EET_CAR_subset, by.x=c("X1","X2"), by.y=c("gene_id_eet", "gene_id_end"), all=FALSE)
    if(is.null(random)) { k=0} else {k=dim(random)[1]}
    data.frame(k,i,j)
  }

stopCluster(cl)

results_eet_car_scrambled_positive<-aggregate(k~i, data=results_eet_car_scrambled,sum)
results_eet_car_scrambled_positive$ratio<-results_eet_car_scrambled_positive$k / (124572756*110)

rm(correlation_COR_PVALUE_EET_CAR_subset,results_eet_car_scrambled,eet,endo,random)

cl <- makeCluster(10)
registerDoParallel(cl)
results_eet_car_scrambled<-data.frame()
results_eet_car_scrambled<- foreach(i = sequence.correlation, .combine='rbind', .inorder=FALSE,  .packages=c("reshape","bigmemory"), .noexport=c("eet", "endo"), .verbose=FALSE) %:%

  foreach(j = 1:rand, .combine='rbind', .inorder=FALSE,.packages=c("reshape","bigmemory"), .noexport=c("eet", "endo"), .verbose=FALSE ) %dopar% {
    
    correlation_COR_PVALUE_EET_CAR_subset<-correlation_COR_PVALUE_EET_CAR[correlation_COR_PVALUE_EET_CAR$pearson_cor > i,]
    
    eet<- attach.big.matrix("fpkm_eet_day_18_AI_a.desc")
    endo<- attach.big.matrix("fpkm_endo_day_18_C_AI_a.desc")
    random<-cor(eet[permutation[j,],],  endo[,],  use = "pairwise.complete.obs", method="pearson")
    random<-melt(random)
    random<-random[random$value < -i,]
    random<-merge(random,correlation_COR_PVALUE_EET_CAR_subset, by.x=c("X1","X2"), by.y=c("gene_id_eet", "gene_id_end"), all=FALSE)
    if(is.null(random)) { k=0} else {k=dim(random)[1]}
    data.frame(k,i,j)
  }

stopCluster(cl)

results_eet_car_scrambled_negative<-aggregate(k~i, data=results_eet_car_scrambled,sum)
results_eet_car_scrambled_negative$ratio<-results_eet_car_scrambled_negative$k / (124572756*110)

rm(correlation_COR_PVALUE_EET_CAR_subset,results_eet_car_scrambled,eet,endo,random)

system("rm fpkm_eet_day_18_AI_a.bin")
system("rm fpkm_eet_day_18_AI_a.desc")
system("rm fpkm_endo_day_18_C_AI_a.bin")
system("rm fpkm_endo_day_18_C_AI_a.desc")


eet<- as.big.matrix(fpkm_eet_day_18_AI_a , type = "double", 
                    separated = FALSE, 
                    backingfile = "fpkm_eet_day_18_AI_a.bin", 
                    descriptorfile = "fpkm_eet_day_18_AI_a.desc", 
                    share=TRUE)
# get a description of the matrix
mdesc_eet <- describe(eet)

endo <- as.big.matrix(fpkm_endo_day_18_IC_AI_a, type = "double", 
                      separated = FALSE, 
                      backingfile = "fpkm_endo_day_18_IC_AI_a.bin", 
                      descriptorfile = "fpkm_endo_day_18_IC_AI_a.desc",
                      share=TRUE)
# get a description of the matrix
mdesc_end<- describe(endo)

permutation<-permutations(n = 5, r = 5, v = 1:5,repeats.allowed=FALSE)
permutation<-permutation[c(4:24,26:48,50:95,97:114,117:118),]

rand<-dim(permutation)[1]

sequence.correlation<-c(0.95,0.99,0.999,0.9999)

cl <- makeCluster(10)
registerDoParallel(cl)

results_eet_icar_scrambled<-data.frame()
results_eet_icar_scrambled<- foreach(i = sequence.correlation, .combine='rbind', .inorder=FALSE,  .packages=c("reshape","bigmemory"), .noexport=c("eet", "endo"), .verbose=FALSE) %:%

  foreach(j = 1:rand, .combine='rbind', .inorder=FALSE,.packages=c("reshape","bigmemory"), .noexport=c("eet", "endo"), .verbose=FALSE ) %dopar% {
    
    correlation_COR_PVALUE_EET_ICAR_subset<-correlation_COR_PVALUE_EET_ICAR[correlation_COR_PVALUE_EET_ICAR$pearson_cor > i,]
    
    eet<- attach.big.matrix("fpkm_eet_day_18_AI_a.desc")
    endo<- attach.big.matrix("fpkm_endo_day_18_IC_AI_a.desc")
    random<-cor(eet[permutation[j,],],  endo[,],  use = "pairwise.complete.obs", method="pearson")
    random<-melt(random)
    random<-random[random$value > i,]
    random<-merge(random,correlation_COR_PVALUE_EET_ICAR_subset, by.x=c("X1","X2"), by.y=c("gene_id_eet", "gene_id_end"), all=FALSE)
    if(is.null(random)) { k=0} else {k=dim(random)[1]}
    data.frame(k,i,j)
  }

stopCluster(cl)

results_eet_icar_scrambled_positive<-aggregate(k~i, data=results_eet_icar_scrambled,sum)
results_eet_icar_scrambled_positive$ratio<-results_eet_icar_scrambled_positive$k / (124610948*110)

rm(correlation_COR_PVALUE_EET_ICAR_subset,results_eet_icar_scrambled,eet,endo,random)

cl <- makeCluster(10)
registerDoParallel(cl)
results_eet_icar_scrambled<-data.frame()
results_eet_icar_scrambled<- foreach(i = sequence.correlation, .combine='rbind', .inorder=FALSE,  .packages=c("reshape","bigmemory"), .noexport=c("eet", "endo"), .verbose=FALSE) %:%

  foreach(j = 1:rand, .combine='rbind', .inorder=FALSE,.packages=c("reshape","bigmemory"), .noexport=c("eet", "endo"), .verbose=FALSE ) %dopar% {
    
    correlation_COR_PVALUE_EET_ICAR_subset<-correlation_COR_PVALUE_EET_ICAR[correlation_COR_PVALUE_EET_ICAR$pearson_cor > i,]
    
    eet<- attach.big.matrix("fpkm_eet_day_18_AI_a.desc")
    endo<- attach.big.matrix("fpkm_endo_day_18_IC_AI_a.desc")
    random<-cor(eet[permutation[j,],],  endo[,],  use = "pairwise.complete.obs", method="pearson")
    random<-melt(random)
    random<-random[random$value < -i,]
    random<-merge(random,correlation_COR_PVALUE_EET_ICAR_subset, by.x=c("X1","X2"), by.y=c("gene_id_eet", "gene_id_end"), all=FALSE)
    if(is.null(random)) { k=0} else {k=dim(random)[1]}
    data.frame(k,i,j)
  }

stopCluster(cl)

results_eet_car_scrambled_negative<-aggregate(k~i, data=results_eet_icar_scrambled,sum)
results_eet_car_scrambled_negative$ratio<-results_eet_car_scrambled_negative$k / (124610948*110)

system("rm fpkm_eet_day_18_AI_a.bin")
system("rm fpkm_eet_day_18_AI_a.desc")
system("rm fpkm_endo_day_18_IC_AI_a.bin")
system("rm fpkm_endo_day_18_IC_AI_a.desc")


rm(correlation_COR_PVALUE_EET_ICAR_subset,results_eet_icar_scrambled,eet,endo,random,mdesc_end,mdesc_eet)
#
results_eet_car_scrambled_positive
```

```
##        i       k        ratio
## 1 0.9500 3866457 2.821613e-04
## 2 0.9900  115923 8.459678e-06
## 3 0.9990    1009 7.363349e-08
## 4 0.9999       5 3.648835e-10
```

```
results_eet_car_scrambled_negative
```

```
##        i      k        ratio
## 1 0.9500 636201 4.641362e-05
## 2 0.9900   7358 5.367980e-07
## 3 0.9990     16 1.167269e-09
## 4 0.9999      0 0.000000e+00
```

```
results_eet_icar_scrambled_positive
```

```
##        i       k        ratio
## 1 0.9500 1891224 1.379730e-04
## 2 0.9900   53592 3.909769e-06
## 3 0.9990     401 2.925469e-08
## 4 0.9999       3 2.188630e-10
```

```
results_eet_car_scrambled_negative
```

```
##        i      k        ratio
## 1 0.9500 636201 4.641362e-05
## 2 0.9900   7358 5.367980e-07
## 3 0.9990     16 1.167269e-09
## 4 0.9999      0 0.000000e+00
```

Load the files contatining expression data

```
fpkm_eet_day_18_AI<-read.table("2017_06_17_fpkm_d18_EET_filtered.txt.bz2",stringsAsFactors=FALSE, header =TRUE)
fpkm_endo_day_18_C_AI<-read.table("2017_06_17_fpkm_d18_ENDO_CAR_filtered.txt.bz2",stringsAsFactors=FALSE, header =TRUE)
fpkm_endo_day_18_IC_AI<-read.table("2017_06_17_fpkm_d18_ENDO_ICAR_filtered.txt.bz2",stringsAsFactors=FALSE, header =TRUE)
annotation.ensembl.symbol<-read.table("2017_12_20_annotation.ensembl.symbol.txt.bz2",stringsAsFactors=FALSE, header =TRUE, sep="\t",quote = "")
```

### Supplemental figure S3

```
correlation_COR_PVALUE_EET_CAR<-correlation_COR_PVALUE_EET_CAR[with(correlation_COR_PVALUE_EET_CAR, order(-pearson_cor)),]

correlation_COR_PVALUE_EET_CAR_subset<-rbind(head(correlation_COR_PVALUE_EET_CAR, n=5), head(correlation_COR_PVALUE_EET_CAR[abs(correlation_COR_PVALUE_EET_CAR$pearson_cor)<0.00001,],n=5), tail(correlation_COR_PVALUE_EET_CAR, n=5))

data_chart_1<-data.frame( stringsAsFactors=FALSE)
data_chart_2<-data.frame( stringsAsFactors=FALSE)
for (i in seq(dim(correlation_COR_PVALUE_EET_CAR_subset)[1])){
  
  gene_eet<-correlation_COR_PVALUE_EET_CAR_subset[i,1]
  gene_endo<-correlation_COR_PVALUE_EET_CAR_subset[i,2]
  
  fpkm_gene_eet<-fpkm_eet_day_18_AI[row.names(fpkm_eet_day_18_AI)==gene_eet,]
  fpkm_gene_endo<-fpkm_endo_day_18_C_AI[row.names(fpkm_endo_day_18_C_AI)==gene_endo,]
  
  data_chart_1<-data.frame(t(fpkm_gene_eet))
  data_chart_1<-cbind(data_chart_1,data.frame(t(fpkm_gene_endo)))
  data_chart_1$chart<-i
  data_chart_1$gene1<-gene_eet
  data_chart_1$gene2<-gene_endo
  
  colnames(data_chart_1)<-c("gene_EET", "geneCAR", "chart", "gene1","gene2")
  
  data_chart_2<-rbind(data_chart_2,data_chart_1)
}


data_chart_2$gene_EET_symbol<-annotation.ensembl.symbol$external_gene_name[match( data_chart_2$gene1, annotation.ensembl.symbol$ensembl_gene_id )]
data_chart_2$gene_END_symbol<-annotation.ensembl.symbol$external_gene_name[match( data_chart_2$gene2, annotation.ensembl.symbol$ensembl_gene_id )]

data_chart_2$gene1<-as.character(data_chart_2$gene1)
data_chart_2$gene2<-as.character(data_chart_2$gene2)

data_chart_2$gene_EET_symbol<-ifelse(is.na(data_chart_2$gene_EET_symbol), data_chart_2$gene1,data_chart_2$gene_EET_symbol)
data_chart_2$gene_END_symbol<-ifelse(is.na(data_chart_2$gene_END_symbol), data_chart_2$gene1,data_chart_2$gene_END_symbol)


data_chart_2$gene_EET_symbol<-ifelse(data_chart_2$gene_EET_symbol=="", data_chart_2$gene1,data_chart_2$gene_EET_symbol)
data_chart_2$gene_END_symbol<-ifelse(data_chart_2$gene_END_symbol=="", data_chart_2$gene1,data_chart_2$gene_END_symbol)


plots <- list()
k<-1
  for (j in c(1:15)){
    data_chart_3<-data_chart_2[data_chart_2$chart %in% j , ]
    plot<-ggplot(data=data_chart_3, aes(x=geneCAR,y=gene_EET ))+
      geom_point(size=0.4)+
      scale_y_continuous(name=data_chart_3$gene_EET_symbol[1])+
      scale_x_continuous(name=data_chart_3$gene_END_symbol[1])+
      theme(aspect.ratio = 1,
            panel.grid.major = element_blank(),
            panel.grid.minor = element_blank(),
            panel.background = element_blank(),
            plot.background  = element_blank(),
            axis.text.x = element_text( colour = 'black' ,size = 7),
            axis.text.y = element_text( colour = 'black',size = 7),
            axis.title= element_text( colour = 'black' ,size = 7, face="italic"),
            axis.ticks = element_line(size=0.1),
            panel.spacing = unit(1, "mm"),
            legend.position="none",
            axis.line=element_line(size = 0.1, colour = "black")
      )
    
    plots[[k]] <- plot
    k<-k+1
  }
 
ggarrange(plotlist =plots,ncol = 5, nrow = 3)
```

### Supplemental figure S4

```
correlation_COR_PVALUE_EET_ICAR<-correlation_COR_PVALUE_EET_ICAR[with(correlation_COR_PVALUE_EET_ICAR, order(-pearson_cor)),]

correlation_COR_PVALUE_EET_ICAR_subset<-rbind(head(correlation_COR_PVALUE_EET_ICAR, n=5), head(correlation_COR_PVALUE_EET_ICAR[abs(correlation_COR_PVALUE_EET_ICAR$pearson_cor)<0.00001,],n=5), tail(correlation_COR_PVALUE_EET_ICAR, n=5))

data_chart_1<-data.frame( stringsAsFactors=FALSE)
data_chart_2<-data.frame( stringsAsFactors=FALSE)
for (i in seq(dim(correlation_COR_PVALUE_EET_ICAR_subset)[1])){
  
  gene_eet<-correlation_COR_PVALUE_EET_ICAR_subset[i,1]
  gene_endo<-correlation_COR_PVALUE_EET_ICAR_subset[i,2]
  
  fpkm_gene_eet<-fpkm_eet_day_18_AI[row.names(fpkm_eet_day_18_AI)==gene_eet,]
  fpkm_gene_endo<-fpkm_endo_day_18_IC_AI[row.names(fpkm_endo_day_18_IC_AI)==gene_endo,]
  
  data_chart_1<-data.frame(t(fpkm_gene_eet))
  data_chart_1<-cbind(data_chart_1,data.frame(t(fpkm_gene_endo)))
  data_chart_1$chart<-i
  data_chart_1$gene1<-gene_eet
  data_chart_1$gene2<-gene_endo
  
  colnames(data_chart_1)<-c("gene_EET", "geneICAR", "chart", "gene1","gene2")
  
  data_chart_2<-rbind(data_chart_2,data_chart_1)
}


data_chart_2$gene_EET_symbol<-annotation.ensembl.symbol$external_gene_name[match( data_chart_2$gene1, annotation.ensembl.symbol$ensembl_gene_id )]
data_chart_2$gene_END_symbol<-annotation.ensembl.symbol$external_gene_name[match( data_chart_2$gene2, annotation.ensembl.symbol$ensembl_gene_id )]

data_chart_2$gene1<-as.character(data_chart_2$gene1)
data_chart_2$gene2<-as.character(data_chart_2$gene2)

data_chart_2$gene_EET_symbol<-ifelse(is.na(data_chart_2$gene_EET_symbol), data_chart_2$gene1,data_chart_2$gene_EET_symbol)
data_chart_2$gene_END_symbol<-ifelse(is.na(data_chart_2$gene_END_symbol), data_chart_2$gene1,data_chart_2$gene_END_symbol)


data_chart_2$gene_EET_symbol<-ifelse(data_chart_2$gene_EET_symbol=="", data_chart_2$gene1,data_chart_2$gene_EET_symbol)
data_chart_2$gene_END_symbol<-ifelse(data_chart_2$gene_END_symbol=="", data_chart_2$gene1,data_chart_2$gene_END_symbol)


plots <- list()
k<-1
for (j in c(1:15)){
  data_chart_3<-data_chart_2[data_chart_2$chart %in% j , ]
  plot<-ggplot(data=data_chart_3, aes(x=geneICAR,y=gene_EET ))+
    geom_point(size=0.4)+
    scale_y_continuous(name=data_chart_3$gene_EET_symbol[1])+
    scale_x_continuous(name=data_chart_3$gene_END_symbol[1])+
    theme(aspect.ratio = 1,
          panel.grid.major = element_blank(),
          panel.grid.minor = element_blank(),
          panel.background = element_blank(),
          plot.background  = element_blank(),
          axis.text.x = element_text( colour = 'black' ,size = 7),
          axis.text.y = element_text( colour = 'black',size = 7),
          axis.title= element_text( colour = 'black' ,size = 7, face="italic"),
          axis.ticks = element_line(size=0.1),
          panel.spacing = unit(1, "mm"),
          legend.position="none",
          axis.line=element_line(size = 0.1, colour = "black")
    )
  
  plots[[k]] <- plot
  k<-k+1
}

ggarrange( plotlist =plots,ncol = 5, nrow = 3)
```

### Supplemental figure S5

```
annotation.ensembl.symbol<-read.table("2017_12_20_annotation.ensembl.symbol.txt.bz2",stringsAsFactors=FALSE, header =TRUE, sep="\t",quote = "")
treshold<-0.95
correlation_COR_PVALUE_EET_CAR_subset<-correlation_COR_PVALUE_EET_CAR[abs(correlation_COR_PVALUE_EET_CAR$pearson_cor) > treshold,]

degree<-data.frame(table(correlation_COR_PVALUE_EET_CAR_subset$gene_id_eet))
ave(degree$Freq)[1]
```

```
## [1] 295.5072
```

```
summary(degree$Freq)[3]
```

```
## Median 
##    101
```

```
degree<-degree[with(degree,order(-Freq)),]
degree_data<-data.frame(table(degree$Freq))
degree_data$Var1<-as.numeric(levels(degree_data$Var1))[degree_data$Var1]
degree<-degree[c(1:10),]
degree_data$ID<-degree$Var1[match(degree_data$Var1,degree$Freq)]
degree_data$symbol<-annotation.ensembl.symbol$external_gene_name[match(degree_data$ID,annotation.ensembl.symbol$ensembl_gene_id)]

plot1<-ggplot(data=degree_data,aes(y=(Freq), x=(Var1)))+
  geom_point( shape=20, size=0.5)+
  geom_text_repel(aes(label=symbol),max.iter = 3e5,fontface = 'italic',angle = 0,nudge_y = 5,
                  na.rm = TRUE, force=TRUE,size = 2, colour = "red",segment.color="gray",segment.size = 0.1,box.padding = unit(0.6, 'lines'))+
  scale_y_continuous(name="Number of genes in EET")+
  scale_x_continuous(name="Number of connections with genes in CAR")+
  #labs(x=expression(paste("Degree of centrality ", (Log[10]), sep=" ")),y=expression(paste("Number of nodes ", (Log[10]), sep=" ")))+
  theme_bw()+
  theme(axis.text = element_text(size = 7, color="black"),
        #axis.title = element_blank(),
        panel.grid.major = element_blank(),
        panel.grid.minor = element_blank()
  )


treshold<-0.95
correlation_COR_PVALUE_EET_ICAR_subset<-correlation_COR_PVALUE_EET_ICAR[abs(correlation_COR_PVALUE_EET_ICAR$pearson_cor) > treshold,]

degree<-data.frame(table(correlation_COR_PVALUE_EET_ICAR_subset$gene_id_eet))
ave(degree$Freq)[1]
```

```
## [1] 266.4669
```

```
summary(degree$Freq)[3]
```

```
## Median 
##    252
```

```
degree<-degree[with(degree,order(-Freq)),]
degree_data<-data.frame(table(degree$Freq))
degree_data$Var1<-as.numeric(levels(degree_data$Var1))[degree_data$Var1]
degree<-degree[c(1:8),]

degree_data$ID<-degree$Var1[match(degree_data$Var1,degree$Freq)]
degree_data$symbol<-annotation.ensembl.symbol$external_gene_name[match(degree_data$ID,annotation.ensembl.symbol$ensembl_gene_id)]

degree_data$symbol[degree_data$symbol =="ROR2"]<-"MYOZ1,ROR2"

plot2<-ggplot(data=degree_data,aes(y=(Freq), x=(Var1)))+
  geom_point( shape=20, size=0.5)+
  geom_text_repel(aes(label=symbol),max.iter = 3e5,fontface = 'italic',angle = 0,nudge_y = 4,nudge_x = -10,
                  na.rm = TRUE, force=TRUE,size = 2, colour = "red",segment.color="gray",segment.size = 0.1,box.padding = unit(0.6, 'lines'))+
  scale_y_continuous(name="Number of genes in EET")+
  scale_x_continuous(name="Number of connections with genes in ICAR")+
  #labs(x=expression(paste("Degree of centrality ", (Log[10]), sep=" ")),y=expression(paste("Number of nodes ", (Log[10]), sep=" ")))+
  theme_bw()+
  theme(axis.text = element_text(size = 7, color="black"),
        #axis.title = element_blank(),
        panel.grid.major = element_blank(),
        panel.grid.minor = element_blank()
  )

correlation_COR_PVALUE_EET_CAR_subset<-correlation_COR_PVALUE_EET_CAR[abs(correlation_COR_PVALUE_EET_CAR$pearson_cor) > treshold,]

degree<-data.frame(table(correlation_COR_PVALUE_EET_CAR_subset$gene_id_end))
ave(degree$Freq)[1]
```

```
## [1] 216.2568
```

```
summary(degree$Freq)[3]
```

```
## Median 
##     63
```

```
degree<-degree[with(degree,order(-Freq)),]
degree_data<-data.frame(table(degree$Freq))
degree_data$Var1<-as.numeric(levels(degree_data$Var1))[degree_data$Var1]
degree<-degree[c(1:10),]
degree_data$ID<-degree$Var1[match(degree_data$Var1,degree$Freq)]
degree_data$symbol<-annotation.ensembl.symbol$external_gene_name[match(degree_data$ID,annotation.ensembl.symbol$ensembl_gene_id)]
degree_data$symbol[degree_data$symbol =="SCO2"]<-"SCO2,TUBGCP4"


plot3<-ggplot(data=degree_data,aes(y=(Freq), x=(Var1)))+
  geom_point( shape=20, size=0.5)+
  geom_text_repel(aes(label=symbol),max.iter = 3e5,fontface = 'italic',angle = 0,nudge_y = 8,nudge_x = 5,
                  na.rm = TRUE, force=TRUE,size = 2, colour = "red",segment.color="gray",segment.size = 0.1,box.padding = unit(0.6, 'lines'))+
  scale_y_continuous(name="Number of genes in CAR")+
  scale_x_continuous(name="Number of connections with genes in EET")+
  #labs(x=expression(paste("Degree of centrality ", (Log[10]), sep=" ")),y=expression(paste("Number of nodes ", (Log[10]), sep=" ")))+
  theme_bw()+
  theme(axis.text = element_text(size = 7, color="black"),
        #axis.title = element_blank(),
        panel.grid.major = element_blank(),
        panel.grid.minor = element_blank(),
        plot.background = element_rect(fill = "transparent",colour = NA),
        panel.background = element_rect(fill = "transparent",colour = NA)
  )

correlation_COR_PVALUE_EET_ICAR_subset<-correlation_COR_PVALUE_EET_ICAR[abs(correlation_COR_PVALUE_EET_ICAR$pearson_cor) > treshold,]
degree<-data.frame(table(correlation_COR_PVALUE_EET_ICAR_subset$gene_id_end))
ave(degree$Freq)[1]
```

```
## [1] 194.9449
```

```
summary(degree$Freq)[3]
```

```
## Median 
##     77
```

```
degree<-degree[with(degree,order(-Freq)),]
degree_data<-data.frame(table(degree$Freq))
degree_data$Var1<-as.numeric(levels(degree_data$Var1))[degree_data$Var1]
degree<-degree[c(1:10),]
degree_data$ID<-degree$Var1[match(degree_data$Var1,degree$Freq)]
degree_data$symbol<-annotation.ensembl.symbol$external_gene_name[match(degree_data$ID,annotation.ensembl.symbol$ensembl_gene_id)]


plot4<-ggplot(data=degree_data,aes(y=(Freq), x=(Var1)))+
  geom_point( shape=20, size=0.5)+
  geom_text_repel(aes(label=symbol),max.iter = 3e5,fontface = 'italic',angle = 0,nudge_y = 5,nudge_x = 2,
                  na.rm = TRUE, force=TRUE,size = 2, colour = "red",segment.color="gray",segment.size = 0.1,box.padding = unit(0.6, 'lines'))+
  scale_y_continuous(name="Number of genes in ICAR")+
  scale_x_continuous(name="Number of connections with genes in EET")+
  #labs(x=expression(paste("Degree of centrality ", (Log[10]), sep=" ")),y=expression(paste("Number of nodes ", (Log[10]), sep=" ")))+
  theme_bw()+
  theme(axis.text = element_text(size = 7, color="black"),
        #axis.title = element_blank(),
        panel.grid.major = element_blank(),
        panel.grid.minor = element_blank(),
        plot.background = element_rect(fill = "transparent",colour = NA),
        panel.background = element_rect(fill = "transparent",colour = NA)
  )

ggarrange( plotlist =list(plot1, plot2, plot3, plot4), nrow=2, ncol = 2)
```

## Figure 2d

```
treshold<-0.9999
correlation_COR_PVALUE_EET_CAR_subset<-correlation_COR_PVALUE_EET_CAR[abs(correlation_COR_PVALUE_EET_CAR$pearson_cor) > treshold,]

#summary(correlation_COR_PVALUE_EET_CAR_subset$pearson_cor)

fpkm_eet_day_18_AI_subset<-fpkm_eet_day_18_AI[rownames(fpkm_eet_day_18_AI) %in% correlation_COR_PVALUE_EET_CAR_subset$gene_id_eet, ]
colnames(fpkm_eet_day_18_AI_subset)<-c("P129","P353","P1091","P1092","P1096")

#number of genes in the EET
dim(fpkm_eet_day_18_AI_subset)[1]
```

```
## [1] 229
```

```
fpkm_eet_day_18_CAR_AI_subset<-fpkm_endo_day_18_C_AI[rownames(fpkm_endo_day_18_C_AI) %in% correlation_COR_PVALUE_EET_CAR_subset$gene_id_end, ]
colnames(fpkm_eet_day_18_CAR_AI_subset)<-c("P129","P353","P1091","P1092","P1096")

#number of genes in CAR
dim(fpkm_eet_day_18_CAR_AI_subset)[1]
```

```
## [1] 218
```

```
#number of unique genes
length(unique(c(rownames(fpkm_eet_day_18_AI_subset), rownames(fpkm_eet_day_18_CAR_AI_subset))))
```

```
## [1] 441
```

```
distance_EET<-as.dist(1-cor(fpkm_eet_day_18_AI_subset, method = "pearson"))
clustering_EET<-flashClust(distance_EET, method="complete")
dendogram_EET<-as.dendrogram(clustering_EET)
distance_CAR<-as.dist(1-cor(fpkm_eet_day_18_CAR_AI_subset, method = "pearson"))
clustering_CAR<-flashClust(distance_CAR, method="complete")
dendogram_CAR<-as.dendrogram(clustering_CAR)
dendrogram_list<-dendlist(rank_branches(dendogram_EET), rank_branches(dendogram_CAR))

correlation_COR_PVALUE_EET_CAR_subset_annotated<-merge(correlation_COR_PVALUE_EET_CAR_subset, annotation.ensembl.symbol, by.x="gene_id_eet", by.y="ensembl_gene_id" , all.x=TRUE, all.y=FALSE)
correlation_COR_PVALUE_EET_CAR_subset_annotated<-merge(correlation_COR_PVALUE_EET_CAR_subset_annotated, annotation.ensembl.symbol, by.x="gene_id_end", by.y="ensembl_gene_id" , all.x=TRUE, all.y=FALSE)
colnames(correlation_COR_PVALUE_EET_CAR_subset_annotated)<-c("gene_id_end","gene_id_eet","pearson_cor", "hgnc_symbol.EET", "description.EET" ,"external_gene_name.EET","hgnc_symbol.CAR","description.CAR", "external_gene_name.CAR"  )
correlation_COR_PVALUE_EET_CAR_subset_annotated<-correlation_COR_PVALUE_EET_CAR_subset_annotated[,c(2,1,3:9)]
```

Testing for the similarity of the dendrograms.

```
set.seed(1236)
mantel(distance_EET, distance_CAR, permutations = 119)
```

```
## 
## Mantel statistic based on Pearson's product-moment correlation 
## 
## Call:
## mantel(xdis = distance_EET, ydis = distance_CAR, permutations = 119) 
## 
## Mantel statistic r: 0.9651 
##       Significance: 0.0083333 
## 
## Upper quantiles of permutations (null model):
##   90%   95% 97.5%   99% 
## 0.510 0.622 0.662 0.891 
## Permutation: free
## Number of permutations: 119
```

```
tanglegram(dendrogram_list, sort = FALSE, common_subtrees_color_lines = TRUE, highlight_distinct_edges  = FALSE, highlight_branches_lwd = FALSE)
```

Testing for gene ontology enrichment for the 441 genes that form the matching dendrograms between conceptus and endometrium

```
annotation.ensembl.symbol<-read.table("2017_12_20_annotation.ensembl.symbol.txt.bz2",stringsAsFactors=FALSE, header =TRUE, sep="\t",quote = "")
annotation.genelength.biomart<-read.table("2017_10_26_annotation.genelength.biomart.txt.bz2",stringsAsFactors=FALSE, header =TRUE, sep="\t")
annotation.GO.biomart<-read.table("2017_10_26_annotation.GO.biomart.txt.bz2",stringsAsFactors=FALSE, header =TRUE, sep="\t")

test.genes<-data.frame(a=unique(c(as.character(correlation_COR_PVALUE_EET_CAR_subset$gene_id_eet),as.character(correlation_COR_PVALUE_EET_CAR_subset$gene_id_end))), stringsAsFactors=FALSE)

all_genes<-data.frame( gene=unique(c(rownames(fpkm_endo_day_18_C_AI),rownames(fpkm_eet_day_18_AI))), stringsAsFactors=FALSE )
rownames(all_genes)<-all_genes$gene

all_genes_numeric<-as.integer(all_genes$gene %in%test.genes$a)
names(all_genes_numeric)<-all_genes$gene

annotation.genelength.biomart <-annotation.genelength.biomart[with(annotation.genelength.biomart, order(ensembl_gene_id, -transcript_length)), ]
annotation.genelength.biomart<-annotation.genelength.biomart[!duplicated(annotation.genelength.biomart$ensembl_gene_id),]
annotation.genelength.biomart <- annotation.genelength.biomart[annotation.genelength.biomart$ensembl_gene_id %in% rownames(all_genes),] 
annotation.genelength.biomart_vector<-annotation.genelength.biomart[,2]
names(annotation.genelength.biomart_vector)<-annotation.genelength.biomart[,1]
annotation.GO.BP.biomart<-annotation.GO.biomart[annotation.GO.biomart$namespace_1003=="biological_process", c(1:2)]

annotation.GO.BP.biomart<-annotation.GO.BP.biomart[annotation.GO.BP.biomart$ensembl_gene_id %in% rownames(all_genes),]

pwf<-nullp(all_genes_numeric, bias.data=annotation.genelength.biomart_vector, plot.fit=FALSE ) 

set.seed(51790)
GO_BP_Cats_EET_CAR_subset<-goseq(pwf,gene2cat=annotation.GO.BP.biomart, method ="Sampling", repcnt = 7000, use_genes_without_cat=FALSE)
GO_BP_Cats_EET_CAR_subset<-GO_BP_Cats_EET_CAR_subset[GO_BP_Cats_EET_CAR_subset$numDEInCat>1,]
GO_BP_Cats_EET_CAR_subset$BY_FDR<-p.adjust(GO_BP_Cats_EET_CAR_subset$over_represented_pvalue, method ="fdr")
GO_BP_Cats_EET_CAR_subset<-GO_BP_Cats_EET_CAR_subset[with(GO_BP_Cats_EET_CAR_subset, order(BY_FDR, -numDEInCat, term)), ]

annotation.GO.BP.biomart_testgenes<-annotation.GO.BP.biomart[annotation.GO.BP.biomart$ensembl_gene_id %in% test.genes$a, ]

GO_BP_Cats_EET_CAR_subset<-merge(GO_BP_Cats_EET_CAR_subset,annotation.GO.BP.biomart_testgenes, by.x="category", by.y="go_id", all.x=TRUE, all.y=FALSE)

GO_BP_Cats_EET_CAR_subset<-merge(GO_BP_Cats_EET_CAR_subset, annotation.ensembl.symbol, by.x="ensembl_gene_id", by.y="ensembl_gene_id", all.x=TRUE, all.y=FALSE)
GO_BP_Cats_EET_CAR_subset<-GO_BP_Cats_EET_CAR_subset[with(GO_BP_Cats_EET_CAR_subset, order(BY_FDR, -numDEInCat)), ]

GO_BP_Cats_EET_CAR_subset<-GO_BP_Cats_EET_CAR_subset[GO_BP_Cats_EET_CAR_subset$numDEInCat>2 & GO_BP_Cats_EET_CAR_subset$BY_FDR<0.13,]
```

Testing for KEGG pathways enrichment for the 441 genes that form the matching dendrograms between conceptus and endometrium

```
test.genes<-data.frame(a=unique(c(as.character(correlation_COR_PVALUE_EET_CAR_subset$gene_id_eet),as.character(correlation_COR_PVALUE_EET_CAR_subset$gene_id_end))), stringsAsFactors=FALSE)

test.genes_entrez<-egIDs <- stack(mget(test.genes$a, org.Bt.egENSEMBL2EG, ifnotfound = NA))

all_genes<-data.frame( gene=unique(c(rownames(fpkm_endo_day_18_C_AI),rownames(fpkm_eet_day_18_AI))), stringsAsFactors=FALSE )
rownames(all_genes)<-all_genes$gene
test.genes_entrez <- stack(mget(test.genes$a, org.Bt.egENSEMBL2EG, ifnotfound = NA))
test.genes_entrez<-test.genes_entrez[complete.cases(test.genes_entrez),]
all_genes_entrez<- stack(mget(all_genes$gene, org.Bt.egENSEMBL2EG, ifnotfound = NA))
all_genes_entrez<-all_genes_entrez[complete.cases(all_genes_entrez),]
all_genes_entrez<-all_genes_entrez[!duplicated(all_genes_entrez$values),]

all_genes_entrez_numeric<-as.integer(all_genes_entrez$values %in% test.genes_entrez$values)
names(all_genes_entrez_numeric)<-all_genes_entrez$values

entrez_kegg<- stack(mget(all_genes_entrez$values, org.Bt.egPATH, ifnotfound = NA))
entrez_kegg<-entrez_kegg[complete.cases(entrez_kegg),]
entrez_kegg<-entrez_kegg[,c(2,1)]
entrez_kegg_test_genes<-entrez_kegg[entrez_kegg$ind %in% test.genes_entrez$values,]

pwf<-nullp(all_genes_entrez_numeric, 'bosTau4','refGene', plot.fit=FALSE ) 

set.seed(8503)
kegg_EET_CAR_subset<-goseq(pwf, gene2cat=entrez_kegg,method ="Sampling", repcnt = 7000, use_genes_without_cat=FALSE)
kegg_EET_CAR_subset<-kegg_EET_CAR_subset[kegg_EET_CAR_subset$numDEInCat>3,]
kegg_EET_CAR_subset$BY_FDR<-p.adjust(kegg_EET_CAR_subset$over_represented_pvalue, method ="fdr")
kegg_EET_CAR_subset<-merge(kegg_EET_CAR_subset,entrez_kegg_test_genes, by.x="category", by.y="values", all.x=TRUE, all.y=FALSE)
kegg_EET_CAR_subset<-merge(kegg_EET_CAR_subset,all_genes_entrez, by.x="ind", by.y="values", all.x=TRUE, all.y=FALSE, suffixes =c('.entrez','.ensembl'))
kegg_EET_CAR_subset<-kegg_EET_CAR_subset[with(kegg_EET_CAR_subset, order(BY_FDR, -numDEInCat)), ]
kegg_EET_CAR_subset<-kegg_EET_CAR_subset[,c(2:8)]
kegg_EET_CAR_subset<-merge(kegg_EET_CAR_subset, annotation.ensembl.symbol, by.x="ind.ensembl", by.y="ensembl_gene_id", all.x=TRUE, all.y=FALSE)
kegg_EET_CAR_subset<-kegg_EET_CAR_subset[with(kegg_EET_CAR_subset, order(BY_FDR, -numDEInCat)), ]

kegg_EET_CAR_subset<-kegg_EET_CAR_subset[kegg_EET_CAR_subset$BY_FDR<0.1,]
```

## Figure 2e

```
genes_interest_1<-sort(GO_BP_Cats_EET_CAR_subset[GO_BP_Cats_EET_CAR_subset$term == 'mRNA processing', 12])
genes_interest_2<-sort(GO_BP_Cats_EET_CAR_subset[GO_BP_Cats_EET_CAR_subset$term == 'chromatin organization', 12])
genes_interest<-unique(c(genes_interest_1,genes_interest_2))
correlation_COR_PVALUE_EET_CAR_subset_annotated<-correlation_COR_PVALUE_EET_CAR_subset_annotated[with(correlation_COR_PVALUE_EET_CAR_subset_annotated, order(-pearson_cor)),]

data_chart_1<-data.frame( stringsAsFactors=FALSE)
data_chart_2<-data.frame( stringsAsFactors=FALSE)
j=1
for (i in genes_interest){
  
correlation_COR_PVALUE_EET_CAR_subset_annotated_a<-correlation_COR_PVALUE_EET_CAR_subset_annotated[correlation_COR_PVALUE_EET_CAR_subset_annotated$external_gene_name.EET == i | correlation_COR_PVALUE_EET_CAR_subset_annotated$external_gene_name.CAR == i ,]
  
gene_eet<-as.character(correlation_COR_PVALUE_EET_CAR_subset_annotated_a[1,1])
gene_endo<-as.character(correlation_COR_PVALUE_EET_CAR_subset_annotated_a[1,2])

fpkm_gene_eet<-fpkm_eet_day_18_AI[row.names(fpkm_eet_day_18_AI)==gene_eet,]
fpkm_gene_endo<-fpkm_endo_day_18_C_AI[row.names(fpkm_endo_day_18_C_AI)==gene_endo,]

data_chart_1<-data.frame(t(fpkm_gene_eet))
data_chart_1<-cbind(data_chart_1,data.frame(t(fpkm_gene_endo)))
data_chart_1$chart<-j
data_chart_1$gene1<-gene_eet
data_chart_1$gene2<-gene_endo

colnames(data_chart_1)<-c("gene_EET", "geneCAR", "chart", "gene1","gene2")

data_chart_2<-rbind(data_chart_2,data_chart_1)

j=j+1
}

data_chart_2$gene_EET_symbol<-annotation.ensembl.symbol$external_gene_name[match( data_chart_2$gene1, annotation.ensembl.symbol$ensembl_gene_id )]
data_chart_2$gene_END_symbol<-annotation.ensembl.symbol$external_gene_name[match( data_chart_2$gene2, annotation.ensembl.symbol$ensembl_gene_id )]
data_chart_2$gene1<-as.character(data_chart_2$gene1)
data_chart_2$gene2<-as.character(data_chart_2$gene2)
data_chart_2$gene_EET_symbol<-ifelse(is.na(data_chart_2$gene_EET_symbol), data_chart_2$gene1,data_chart_2$gene_EET_symbol)
data_chart_2$gene_END_symbol<-ifelse(is.na(data_chart_2$gene_END_symbol), data_chart_2$gene1,data_chart_2$gene_END_symbol)
data_chart_2$gene_EET_symbol<-ifelse(data_chart_2$gene_EET_symbol=="", data_chart_2$gene1,data_chart_2$gene_EET_symbol)
data_chart_2$gene_END_symbol<-ifelse(data_chart_2$gene_END_symbol=="", data_chart_2$gene1,data_chart_2$gene_END_symbol)

plots <- list()
k<-1
for (j in c(1:8)){
data_chart_3<-data_chart_2[data_chart_2$chart %in% j , ]
plot<-ggplot(data=data_chart_3, aes(x=geneCAR,y=gene_EET))+
      geom_point(size=1)+
      scale_y_continuous(name=data_chart_3$gene_EET_symbol[1],breaks= pretty_breaks(3))+
      scale_x_continuous(name=data_chart_3$gene_END_symbol[1],breaks= pretty_breaks(3))+
      theme(aspect.ratio = 1,
            panel.grid.major = element_blank(),
            panel.grid.minor = element_blank(),
            panel.background = element_blank(),
            plot.background  = element_blank(),
            axis.text.x = element_text( colour = 'black' ,size = 13),
            axis.text.y = element_text( colour = 'black',size = 13),
            axis.title= element_text( colour = 'black' ,size = 13, face="italic"),
            axis.ticks = element_line(size=0.1),
            panel.spacing = unit(0, "mm"),
            legend.position="none",
            axis.line=element_line(size = 0.8, colour = "black")
      )
    
    plots[[k]] <- plot
    k<-k+1
  }
ggarrange( plotlist =plots)
```

## Figure 2f

```
genes_interest<-sort(unique(as.character(kegg_EET_CAR_subset[kegg_EET_CAR_subset$category == '03013', 10])))
correlation_COR_PVALUE_EET_CAR_subset_annotated<-correlation_COR_PVALUE_EET_CAR_subset_annotated[with(correlation_COR_PVALUE_EET_CAR_subset_annotated, order(-pearson_cor)),]
data_chart_1<-data.frame( stringsAsFactors=FALSE)
data_chart_2<-data.frame( stringsAsFactors=FALSE)
j=1
for (i in genes_interest){
  
correlation_COR_PVALUE_EET_CAR_subset_annotated_a<-correlation_COR_PVALUE_EET_CAR_subset_annotated[correlation_COR_PVALUE_EET_CAR_subset_annotated$external_gene_name.EET == i | correlation_COR_PVALUE_EET_CAR_subset_annotated$external_gene_name.CAR == i ,]
  
gene_eet<-as.character(correlation_COR_PVALUE_EET_CAR_subset_annotated_a[1,1])
gene_endo<-as.character(correlation_COR_PVALUE_EET_CAR_subset_annotated_a[1,2])
fpkm_gene_eet<-fpkm_eet_day_18_AI[row.names(fpkm_eet_day_18_AI)==gene_eet,]
fpkm_gene_endo<-fpkm_endo_day_18_C_AI[row.names(fpkm_endo_day_18_C_AI)==gene_endo,]
  
data_chart_1<-data.frame(t(fpkm_gene_eet))
data_chart_1<-cbind(data_chart_1,data.frame(t(fpkm_gene_endo)))
data_chart_1$chart<-j
data_chart_1$gene1<-gene_eet
data_chart_1$gene2<-gene_endo
  colnames(data_chart_1)<-c("gene_EET", "geneCAR", "chart", "gene1","gene2")
  
data_chart_2<-rbind(data_chart_2,data_chart_1)
  j=j+1
}

data_chart_2$gene_EET_symbol<-annotation.ensembl.symbol$external_gene_name[match( data_chart_2$gene1, annotation.ensembl.symbol$ensembl_gene_id )]
data_chart_2$gene_END_symbol<-annotation.ensembl.symbol$external_gene_name[match( data_chart_2$gene2, annotation.ensembl.symbol$ensembl_gene_id )]
data_chart_2$gene1<-as.character(data_chart_2$gene1)
data_chart_2$gene2<-as.character(data_chart_2$gene2)
data_chart_2$gene_EET_symbol<-ifelse(is.na(data_chart_2$gene_EET_symbol), data_chart_2$gene1,data_chart_2$gene_EET_symbol)
data_chart_2$gene_END_symbol<-ifelse(is.na(data_chart_2$gene_END_symbol), data_chart_2$gene1,data_chart_2$gene_END_symbol)
data_chart_2$gene_EET_symbol<-ifelse(data_chart_2$gene_EET_symbol=="", data_chart_2$gene1,data_chart_2$gene_EET_symbol)
data_chart_2$gene_END_symbol<-ifelse(data_chart_2$gene_END_symbol=="", data_chart_2$gene1,data_chart_2$gene_END_symbol)


plots <- list()
k<-1
for (j in c(1:10)){
  data_chart_3<-data_chart_2[data_chart_2$chart %in% j , ]
  plot<-ggplot(data=data_chart_3, aes(x=geneCAR,y=gene_EET))+
    geom_point(size=0.5)+
    scale_y_continuous(name=data_chart_3$gene_EET_symbol[1])+
    scale_x_continuous(name=data_chart_3$gene_END_symbol[1])+
    theme(aspect.ratio = 1,
          panel.grid.major = element_blank(),
          panel.grid.minor = element_blank(),
          panel.background = element_blank(),
          plot.background  = element_blank(),
          axis.text.x = element_text( colour = 'black' ,size = 8),
          axis.text.y = element_text( colour = 'black',size = 8),
          axis.title= element_text( colour = 'black' ,size = 8, face="italic"),
          axis.ticks = element_line(size=0.1),
          panel.spacing = unit(0, "mm"),
          legend.position="none",
          axis.line=element_line(size = 0.5, colour = "black")
    )
  
  plots[[k]] <- plot
  k<-k+1
}

ggarrange( plotlist =plots,ncol = 5, nrow = 2)
```

### Supplemental figure S6

Assessing the probability of false positive results from GO results by a bootstrapping procedure.

```
cl <- makeCluster(10)
registerDoParallel(cl)

results<-foreach(j = 1:2000, .combine='rbind', .inorder=FALSE,.packages=c("multtest","goseq"), .verbose=FALSE ) %dopar% {

test.genes<-data.frame(a=sample(unique(c(rownames(fpkm_endo_day_18_C_AI),rownames(fpkm_eet_day_18_AI))),441), stringsAsFactors=FALSE)
all_genes<-data.frame( gene=unique(c(rownames(fpkm_endo_day_18_C_AI),rownames(fpkm_eet_day_18_AI))), stringsAsFactors=FALSE )
rownames(all_genes)<-all_genes$gene

all_genes_numeric<-as.integer(all_genes$gene %in%test.genes$a)
names(all_genes_numeric)<-all_genes$gene

pwf<-nullp(all_genes_numeric, bias.data=annotation.genelength.biomart_vector, plot.fit=FALSE ) 
GO_BP_Cats_EET_CAR_subset<-goseq(pwf,gene2cat=annotation.GO.BP.biomart, method ="Wallenius", use_genes_without_cat=FALSE)
GO_BP_Cats_EET_CAR_subset<-GO_BP_Cats_EET_CAR_subset[GO_BP_Cats_EET_CAR_subset$numDEInCat>=1,]
GO_BP_Cats_EET_CAR_subset$BY_FDR<-p.adjust(GO_BP_Cats_EET_CAR_subset$over_represented_pvalue, method ="fdr")
GO_BP_Cats_EET_CAR_subset[c("term","BY_FDR")]
}
stopCluster(cl)
```

```
plot1<-ggplot(results, aes(BY_FDR))+
geom_histogram(binwidth=0.005)+
scale_x_continuous(breaks=c(0,0.25,0.5,0.75,1))+
theme(
          axis.text.x = element_text( colour = 'black' ,size = 12),
          axis.text.y = element_text( colour = 'black',size = 12),
          axis.title.y= element_text( colour = 'black' ,size = 12),
          axis.title.x= element_blank()
)

plot2<-ggplot(data=na.omit(results[results$term == 'mRNA processing',]), aes(y=BY_FDR,x=0))+
geom_jitter(width=c(0.02,0.01), color='black', size=0.5, alpha=0.5, point=16)+
geom_point(aes(x=0, y=0.1297709),color='red', size=1,  point=16)+
coord_flip()+
scale_y_continuous(limits=c(0,1), breaks=c(0,0.25,0.5,0.75,1))+
theme(
          axis.text.x = element_text( colour = 'black' ,size = 12),
          axis.ticks.y = element_blank(),
          axis.text.y = element_blank(),
          axis.title.x= element_blank(),
          axis.title.y= element_blank(),
          plot.margin=unit(c(0,0.2,0.5,2),"cm")
)


plot3<-ggplot(data=na.omit(results[results$term == 'chromatin organization',]), aes(y=BY_FDR,x=0))+
geom_jitter(width=c(0.02,0.01), color='black', size=0.5, alpha=0.5, point=16)+
geom_point(aes(x=0, y=0.1297709),color='red', size=1,  point=16)+
coord_flip()+
scale_y_continuous(name="False discovery rate",limits=c(0,1), breaks=c(0,0.25,0.5,0.75,1))+
theme(
          axis.text.x = element_text( colour = 'black' ,size = 12),
          axis.ticks.y = element_blank(),
          axis.text.y = element_blank(),
          axis.title.x= element_text( colour = 'black' ,size = 12),
          axis.title.y= element_blank(),
          plot.margin=unit(c(0,0.2,0.5,2),"cm")
)

ggarrange( plotlist =list(plot1, plot2, plot3), nrow=3, ncol = 1, heights=c(2.5,1,1))
```

Probability of occurrence of KEGG categories P values lower than the result obtained for the real data.

```
dim(results[results$BY_FDR<0.1297709,])[1]/dim(results)[1]
```

```
## [1] 0.0005020508
```

Assessing the probability of false positive results from KEGG results by bootstrapping procedure.

```
all_genes<-data.frame( gene=unique(c(rownames(fpkm_endo_day_18_C_AI),rownames(fpkm_eet_day_18_AI))), stringsAsFactors=FALSE )
rownames(all_genes)<-all_genes$gene
all_genes_entrez<- stack(mget(all_genes$gene, org.Bt.egENSEMBL2EG, ifnotfound = NA))
all_genes_entrez<-all_genes_entrez[complete.cases(all_genes_entrez),]
all_genes_entrez<-all_genes_entrez[!duplicated(all_genes_entrez$values),]

cl <- makeCluster(10)
registerDoParallel(cl)

results<-foreach(j = 1:2000, .combine='rbind', .inorder=FALSE,.packages=c("multtest","goseq","org.Bt.eg.db"), .verbose=FALSE ) %dopar% {

test.genes<-data.frame(a=sample(unique(c(rownames(fpkm_endo_day_18_C_AI),rownames(fpkm_eet_day_18_AI))),441), stringsAsFactors=FALSE)
test.genes_entrez<-egIDs <- stack(mget(test.genes$a, org.Bt.egENSEMBL2EG, ifnotfound = NA))
test.genes_entrez <- stack(mget(test.genes$a, org.Bt.egENSEMBL2EG, ifnotfound = NA))
test.genes_entrez<-test.genes_entrez[complete.cases(test.genes_entrez),]
all_genes_entrez_numeric<-as.integer(all_genes_entrez$values %in% test.genes_entrez$values)
names(all_genes_entrez_numeric)<-all_genes_entrez$values

entrez_kegg<- stack(mget(all_genes_entrez$values, org.Bt.egPATH, ifnotfound = NA))
entrez_kegg<-entrez_kegg[complete.cases(entrez_kegg),]
entrez_kegg<-entrez_kegg[,c(2,1)]
entrez_kegg_test_genes<-entrez_kegg[entrez_kegg$ind %in% test.genes_entrez$values,]
pwf<-nullp(all_genes_entrez_numeric, 'bosTau4','refGene', plot.fit=FALSE ) 
kegg_EET_CAR_subset<-goseq(pwf, gene2cat=entrez_kegg,method ="Wallenius", use_genes_without_cat=FALSE)
kegg_EET_CAR_subset$BY_FDR<-p.adjust(kegg_EET_CAR_subset$over_represented_pvalue, method ="fdr")
kegg_EET_CAR_subset<-kegg_EET_CAR_subset[,c("category","BY_FDR")]
}
stopCluster(cl)
```

```
plot1<-ggplot(results, aes(BY_FDR))+
geom_histogram(binwidth=0.005)+
scale_x_continuous(breaks=c(0,0.25,0.5,0.75,1))+
theme(
          axis.text.x = element_text( colour = 'black' ,size = 12),
          axis.text.y = element_text( colour = 'black',size = 12),
          axis.title.y= element_text( colour = 'black' ,size = 12),
          axis.title.x= element_blank()
)

plot2<-ggplot(data=na.omit(results[results$category == '03013',]), aes(y=BY_FDR,x=0))+
geom_jitter(width=c(0.02,0.01), color='black', size=0.5, alpha=0.5, point=16)+
geom_point(aes(x=0, y=0.07713184),color='red', size=1,  point=16)+
coord_flip()+
scale_y_continuous(limits=c(0,1), breaks=c(0,0.25,0.5,0.75,1))+
theme(
          axis.text.x = element_text( colour = 'black' ,size = 12),
          axis.ticks.y = element_blank(),
          axis.text.y = element_blank(),
          axis.title.x= element_blank(),
          axis.title.y= element_blank(),
          plot.margin=unit(c(0,0.2,0.5,2),"cm")
)
ggarrange( plotlist =list(plot1, plot2), nrow=2, ncol = 1, heights=c(2,1))
```

Probability of occurrence of KEGG categories P values lower than the result obtained for the real data.

```
dim(results[results$BY_FDR<0.07713184,])[1]/dim(results)[1]
```

```
## [1] 0.0002388393
```

## Figure 3a

Clustering of EET and CAR genes based on the correlations

```
pearson_correlation_EET<-cor(fpkm_eet_day_18_AI_a,  fpkm_endo_day_18_C_AI_a,  use = "pairwise.complete.obs", method="pearson")
dissimilarity<-as.matrix(parDist(pearson_correlation_EET, method = "euclidean", diag = TRUE, upper = TRUE))
rownames(dissimilarity)<-colnames(fpkm_eet_day_18_AI_a)
colnames(dissimilarity)<-colnames(fpkm_eet_day_18_AI_a)
geneTree_eet <- flashClust(as.dist(dissimilarity), method = "average")
rm(dissimilarity)

pearson_correlation_EDO<-cor(fpkm_endo_day_18_C_AI_a,fpkm_eet_day_18_AI_a,  use = "pairwise.complete.obs", method="pearson")
dissimilarity<- as.matrix(parDist(pearson_correlation_EDO, method = "euclidean",diag = TRUE, upper = TRUE))
rownames(dissimilarity)<-colnames(fpkm_endo_day_18_C_AI_a)
colnames(dissimilarity)<-colnames(fpkm_endo_day_18_C_AI_a)
geneTree_endo <- flashClust(as.dist(dissimilarity), method = "average")
rm(dissimilarity)
pearson_correlation_EET_heatmap<-pearson_correlation_EET[geneTree_eet$order,geneTree_endo$order ]
```

```
Heatmap(pearson_correlation_EET_heatmap,
        name="correlation",
        cluster_rows= FALSE,
        cluster_columns = FALSE,
        show_row_names = FALSE,
        show_column_names = FALSE,
        col = colorRamp2(c(-1, 0, 1), c("blue", "white", "red"))
)
```

The final output of the next section “go.results3\_EET” was used to produce Table S2

```
annotation.ensembl.symbol<-read.table("2017_12_20_annotation.ensembl.symbol.txt.bz2",stringsAsFactors=FALSE, header =TRUE, sep="\t",quote = "")
annotation.genelength.biomart<-read.table("2017_12_20_annotation.genelength.biomart.txt.bz2",stringsAsFactors=FALSE, header =TRUE, sep="\t",quote = "")
annotation.GO.biomart<-read.table("2017_12_20_annotation.GO.biomart.txt.bz2",stringsAsFactors=FALSE, header =TRUE, sep="\t",quote = "")

all_genes<-data.frame( gene=rownames(fpkm_eet_day_18_AI), stringsAsFactors=FALSE )
rownames(all_genes)<-all_genes$gene

annotation.genelength.biomart <-annotation.genelength.biomart[with(annotation.genelength.biomart, order(ensembl_gene_id, -transcript_length)), ]
annotation.genelength.biomart<-annotation.genelength.biomart[!duplicated(annotation.genelength.biomart$ensembl_gene_id),]
annotation.genelength.biomart <- annotation.genelength.biomart[annotation.genelength.biomart$ensembl_gene_id %in% rownames(all_genes),] 
annotation.genelength.biomart_vector<-annotation.genelength.biomart[,2]
names(annotation.genelength.biomart_vector)<-annotation.genelength.biomart[,1]

annotation.GO.BP.biomart<-annotation.GO.biomart[annotation.GO.biomart$namespace_1003=="biological_process", c(1:2)]

annotation.GO.BP.biomart<-annotation.GO.BP.biomart[annotation.GO.BP.biomart$ensembl_gene_id %in% rownames(all_genes),]

summary(geneTree_eet$height)
clusters.cut<-seq(1,50, 1) 

genes_at_heigh<-cutree(geneTree_eet, h=clusters.cut)
genes_at_heigh<-data.frame(genes_at_heigh)
head(genes_at_heigh, n=20)


go.results2<-data.frame()
  
  for (j in seq(1:max(genes_at_heigh$X23))){
    
    genes_at_heigh_a<-genes_at_heigh[genes_at_heigh$X23==j,]
    
    test.genes<-data.frame(a=rownames(genes_at_heigh_a), stringsAsFactors=FALSE)
    
    if (length(test.genes$a) >3){
      
      annotation.GO.BP.biomart_testgenes<-annotation.GO.BP.biomart[annotation.GO.BP.biomart$ensembl_gene_id %in% test.genes$a, ]
      
      if( length(unique(annotation.GO.BP.biomart_testgenes$ensembl_gene_id)) >= 3 )  {
        
        all_genes_numeric<-as.integer(all_genes$gene %in%test.genes$a)
        names(all_genes_numeric)<-all_genes$gene
        
        
        pwf<-try(nullp(all_genes_numeric, bias.data=annotation.genelength.biomart_vector, plot.fit=FALSE ), silent =TRUE)
        if ('try-error' %in% class(pwf)) { pwf<-try(nullp(all_genes_numeric,'bosTau4','ensGene', plot.fit=FALSE ) )}
        
        if(!('try-error' %in% class(pwf))){
          
          GO_BP_Cats<-goseq(pwf,gene2cat=annotation.GO.BP.biomart, method ="Wallenius", use_genes_without_cat=FALSE)
          
          GO_BP_Cats<-GO_BP_Cats[GO_BP_Cats$numDEInCat>2,]
          
          if( dim(GO_BP_Cats)[1] != 0) {
            
            GO_BP_Cats$BY_FDR<-p.adjust(GO_BP_Cats$over_represented_pvalue, method ="BY")
            GO_BP_Cats$height  <- "X23"
            GO_BP_Cats$cluster <- j
            
            GO_BP_Cats<-merge(GO_BP_Cats,annotation.GO.BP.biomart_testgenes, by.x="category", by.y="go_id", all.x=TRUE, all.y=FALSE)
            
            go.results2<-rbind(go.results2,GO_BP_Cats)
          }
        }
        rm(annotation.GO.BP.biomart_testgenes,GO_BP_Cats, test.genes)
      }
    }
  }

go.results3<-go.results2[go.results2$BY_FDR < 0.2,]
go.results3<-go.results3[with(go.results3, order(BY_FDR)), ]
go.results3<-merge(go.results3, annotation.ensembl.symbol, by.x="ensembl_gene_id", by.y="ensembl_gene_id", all.x=TRUE, all.y=FALSE)
go.results3_EET<-go.results3[with(go.results3, order(BY_FDR)), ]
```

The final output of the next section “go.results3\_CAR” was used to produce Table S3

```
all_genes<-data.frame( gene=rownames(fpkm_endo_day_18_C_AI), stringsAsFactors=FALSE )
rownames(all_genes)<-all_genes$gene

annotation.genelength.biomart <-annotation.genelength.biomart[with(annotation.genelength.biomart, order(ensembl_gene_id, -transcript_length)), ]
annotation.genelength.biomart<-annotation.genelength.biomart[!duplicated(annotation.genelength.biomart$ensembl_gene_id),]
annotation.genelength.biomart <- annotation.genelength.biomart[annotation.genelength.biomart$ensembl_gene_id %in% rownames(all_genes),] 
annotation.genelength.biomart_vector<-annotation.genelength.biomart[,2]
names(annotation.genelength.biomart_vector)<-annotation.genelength.biomart[,1]
annotation.GO.BP.biomart<-annotation.GO.biomart[annotation.GO.biomart$namespace_1003=="biological_process", c(1:2)]
annotation.GO.BP.biomart<-annotation.GO.BP.biomart[annotation.GO.BP.biomart$ensembl_gene_id %in% rownames(all_genes),]

genes_at_heigh<-cutree(geneTree_endo, h=clusters.cut)
genes_at_heigh<-data.frame(genes_at_heigh)

go.results2<-data.frame()

  
  for (j in seq(1:max(genes_at_heigh$X27))){
    
    genes_at_heigh_a<-genes_at_heigh[genes_at_heigh$X27==j,]
    
    test.genes<-data.frame(a=rownames(genes_at_heigh_a), stringsAsFactors=FALSE)
    
    if (length(test.genes$a) >3){
      
      annotation.GO.BP.biomart_testgenes<-annotation.GO.BP.biomart[annotation.GO.BP.biomart$ensembl_gene_id %in% test.genes$a, ]
      
      if( length(unique(annotation.GO.BP.biomart_testgenes$ensembl_gene_id)) >= 3 )  {
        
        all_genes_numeric<-as.integer(all_genes$gene %in%test.genes$a)
        names(all_genes_numeric)<-all_genes$gene
        
        
        pwf<-try(nullp(all_genes_numeric, bias.data=annotation.genelength.biomart_vector, plot.fit=FALSE ), silent =TRUE)
        if ('try-error' %in% class(pwf)) { pwf<-try(nullp(all_genes_numeric,'bosTau4','ensGene', plot.fit=FALSE ) )}
        
        if(!('try-error' %in% class(pwf))){
          
          GO_BP_Cats<-goseq(pwf,gene2cat=annotation.GO.BP.biomart, method ="Wallenius", use_genes_without_cat=FALSE)
          
          GO_BP_Cats<-GO_BP_Cats[GO_BP_Cats$numDEInCat>2,]
          
          if( dim(GO_BP_Cats)[1] != 0) {
            
            GO_BP_Cats$BY_FDR<-p.adjust(GO_BP_Cats$over_represented_pvalue, method ="BY")
            GO_BP_Cats$height  <- "X27"
            GO_BP_Cats$cluster <- j
            
            GO_BP_Cats<-merge(GO_BP_Cats,annotation.GO.BP.biomart_testgenes, by.x="category", by.y="go_id", all.x=TRUE, all.y=FALSE)
            
            go.results2<-rbind(go.results2,GO_BP_Cats)
            
          }
        }
        rm(annotation.GO.BP.biomart_testgenes,GO_BP_Cats, test.genes)
      }
    }
  }


go.results3<-go.results2[go.results2$BY_FDR < 0.2,]
go.results3<-go.results3[with(go.results3, order(BY_FDR)), ]
go.results3<-merge(go.results3, annotation.ensembl.symbol, by.x="ensembl_gene_id", by.y="ensembl_gene_id", all.x=TRUE, all.y=FALSE)
go.results3_CAR<-go.results3[with(go.results3, order(BY_FDR)), ]
```

## Figure 3b

```
genes_at_heigh<-cutree(geneTree_eet, h=clusters.cut)
genes_at_heigh<-data.frame(genes_at_heigh)
genes_at_heigh<-data.frame(genes_at_heigh)
genes_at_heigh_color_EET<-data.frame(genes_at_heigh)
genes_at_heigh_color_EET<-genes_at_heigh_color_EET[geneTree_eet$order,] 
genes_at_heigh_color_EET$X23[ !(genes_at_heigh_color_EET$X23 %in% unique(c(go.results3_EET$cluster)))]<- 0
data_frame_annotation_EET<-data.frame(cluster = genes_at_heigh_color_EET$X23)

colors_row_BP<-list(cluster=c(
  "0" = "#FFFFFF",
  "1" = "#00141c",
  "2" = "#0075fd",
  "7" = "#bcffbd",
  "11" =    "#018699",
  "12" =    "#7a3900",
  "13" =    "#00501e",
  "14" =    "#00386d",
  "17" =    "#012600",
  "19" =    "#dbffd3",
  "20" =    "#490023",
  "21" =    "#97bbff",
  "24" =    "#00764a",
  "25" =    "#e8ffa6",
  "26" =    "#430039",
  "27" =    "#00634f",
  "28" =    "#ffc660",
  "29" =    "#99f7ff",
  "30" =    "#9d8800",
  "31" =    "#270700",
  "32" =    "#ffa4b2",
  "33" =    "#2a0019",
  "34" =    "#98ff8e",
  "35" =    "#77a6ff",
  "38" =    "#ffdcca",
  "39" =    "#ee00d2",
  "41" =    "#ff4f6a",
  "46" =    "#000e8e",
  "50" =    "#001957",
  "54" =    "#ddd3ff",
  "59" =    "#009641",
  "67" =    "#cc000e",
  "70" =    "#df7bff",
  "71" =    "#00524f",
  "73" =    "#3a2300",
  "76" =    "#ffa871",
  "101" =   "#661acb" 
))

font_size=6

row_annotation <- HeatmapAnnotation(df = data_frame_annotation_EET, col = colors_row_BP, which="row", width = unit(0.4, "cm"),gap = unit(0, "mm"),
                                    annotation_legend_param=list(title="EET clusters",legend_direction="horizontal",nrow = 2,title_gp = gpar(fontsize = font_size), labels_gp = gpar(fontsize = font_size), grid_height = unit(2, "mm"), grid_width = unit(2, "mm")))


clusters.cut<-seq(1,50, 1) 
genes_at_heigh<-cutree(geneTree_endo, h=clusters.cut)
genes_at_heigh<-data.frame(genes_at_heigh)

genes_at_heigh<-data.frame(genes_at_heigh)
genes_at_heigh_color_CAR<-data.frame(genes_at_heigh)
genes_at_heigh_color_CAR<-genes_at_heigh_color_CAR[geneTree_endo$order,] 
genes_at_heigh_color_CAR$X27[ !(genes_at_heigh_color_CAR$X27 %in% unique(c(go.results3_CAR$cluster)))]<- 0
data_frame_annotation_CAR<-data.frame(cluster = genes_at_heigh_color_CAR$X27)

colors_column_BP<-list(cluster=c(
  "0" = "#FFFFFF",
  "3" = "#4eff79",
  "4" = "#b84800",
  "5" = "#029398",
  "7" = "#8bdcff",
  "8" = "#fff415",
  "9" = "#01a687",
  "10" =    "#fff7cd",
  "11" =    "#02e89b",
  "12" =    "#e5002a",
  "13" =    "#228b00",
  "14" =    "#83d100",
  "15" =    "#fa8bff",
  "16" =    "#763a0b",
  "19" =    "#53474f",
  "20" =    "#673870",
  "23" =    "#b9ffc4",
  "21" =    "#97bbff",
  "24" =    "#8d78ff",
  "25" =    "#4c6100",
  "26" =    "#0142af",
  "27" =    "#326e00",
  "29" =    "#ffa636",
  "32" =    "#896000",
  "33" =    "#ffb87e",
  "34" =    "#4c3a9b",
  "36" =    "#ff7592",
  "37" =    "#f3b9ff",
  "38" =    "#73ae00",
  "39" =    "#930086",
  "40" =    "#9c005d",
  "43" =    "#d5009a",
  "44" =    "#ff3a81",
  "48" =    "#9ebaff"
))

column_annotation <- HeatmapAnnotation(df = data_frame_annotation_CAR, col = colors_column_BP, which="column", height=unit(0.4, "cm"),width = unit(0.1, "cm"),gap = unit(0, "mm"),
                                       annotation_legend_param=list(title="CAR clusters",title_position = "topcenter",legend_position="center",legend_direction="horizontal",nrow = 2,title_gp = gpar(fontsize =font_size),labels_gp = gpar(fontsize = font_size),grid_height = unit(2, "mm"), grid_width = unit(2, "mm")))

pearson_correlation_EET_heatmap<-pearson_correlation_EET[geneTree_eet$order,geneTree_endo$order ]

pearson_correlation_EET_heatmap[(pearson_correlation_EET_heatmap >= -0.95 &  pearson_correlation_EET_heatmap <= 0.95)]<-0

heatmap_pearson_eet_CAR<- Heatmap(pearson_correlation_EET_heatmap,
        name="correlation",
        cluster_rows= FALSE,
        cluster_columns = FALSE,
        show_row_names = FALSE,
        show_column_names = FALSE,
        col = colorRamp2(c(-1, 0, 1), c("blue", "white", "red")),
        bottom_annotation=column_annotation
)

draw(heatmap_pearson_eet_CAR + row_annotation , annotation_legend_side = "bottom",heatmap_legend_side = "bottom")
```

## Figure 4a

Clustering of EET and ICAR genes based on the correlations

```
pearson_correlation_EET<-cor(fpkm_eet_day_18_AI_a,  fpkm_endo_day_18_IC_AI_a,  use = "pairwise.complete.obs", method="pearson")
dissimilarity<-as.matrix(parDist(pearson_correlation_EET, method = "euclidean", diag = TRUE, upper = TRUE))
rownames(dissimilarity)<-colnames(fpkm_eet_day_18_AI_a)
colnames(dissimilarity)<-colnames(fpkm_eet_day_18_AI_a)
geneTree_eet <- flashClust(as.dist(dissimilarity), method = "average")
rm(dissimilarity)

pearson_correlation_EDO<-cor(fpkm_endo_day_18_IC_AI_a,fpkm_eet_day_18_AI_a,  use = "pairwise.complete.obs", method="pearson")
dissimilarity<- as.matrix(parDist(pearson_correlation_EDO, method = "euclidean",diag = TRUE, upper = TRUE))
rownames(dissimilarity)<-colnames(fpkm_endo_day_18_IC_AI_a)
colnames(dissimilarity)<-colnames(fpkm_endo_day_18_IC_AI_a)
geneTree_endo <- flashClust(as.dist(dissimilarity), method = "average")
rm(dissimilarity,pearson_correlation_EDO)

pearson_correlation_EET_heatmap<-pearson_correlation_EET[geneTree_eet$order,geneTree_endo$order ]
```

```
Heatmap(pearson_correlation_EET_heatmap,
        name="correlation",
        cluster_rows= FALSE,
        cluster_columns = FALSE,
        show_row_names = FALSE,
        show_column_names = FALSE,
        col = colorRamp2(c(-1, 0, 1), c("blue", "white", "red"))
)
```

The final output of the next section “go.results3\_EET” was used to produce Table S5

```
annotation.ensembl.symbol<-read.table("2017_12_20_annotation.ensembl.symbol.txt.bz2",stringsAsFactors=FALSE, header =TRUE, sep="\t",quote = "")
annotation.genelength.biomart<-read.table("2017_12_20_annotation.genelength.biomart.txt.bz2",stringsAsFactors=FALSE, header =TRUE, sep="\t",quote = "")
annotation.GO.biomart<-read.table("2017_12_20_annotation.GO.biomart.txt.bz2",stringsAsFactors=FALSE, header =TRUE, sep="\t",quote = "")

all_genes<-data.frame( gene=rownames(fpkm_eet_day_18_AI), stringsAsFactors=FALSE )
rownames(all_genes)<-all_genes$gene

annotation.genelength.biomart <-annotation.genelength.biomart[with(annotation.genelength.biomart, order(ensembl_gene_id, -transcript_length)), ]
annotation.genelength.biomart<-annotation.genelength.biomart[!duplicated(annotation.genelength.biomart$ensembl_gene_id),]
annotation.genelength.biomart <- annotation.genelength.biomart[annotation.genelength.biomart$ensembl_gene_id %in% rownames(all_genes),] 
annotation.genelength.biomart_vector<-annotation.genelength.biomart[,2]
names(annotation.genelength.biomart_vector)<-annotation.genelength.biomart[,1]

annotation.GO.BP.biomart<-annotation.GO.biomart[annotation.GO.biomart$namespace_1003=="biological_process", c(1:2)]

annotation.GO.BP.biomart<-annotation.GO.BP.biomart[annotation.GO.BP.biomart$ensembl_gene_id %in% rownames(all_genes),]

summary(geneTree_eet$height)
clusters.cut<-seq(1,50, 1) 

genes_at_heigh<-cutree(geneTree_eet, h=clusters.cut)
genes_at_heigh<-data.frame(genes_at_heigh)
head(genes_at_heigh, n=20)


go.results2<-data.frame()
  
  for (j in seq(1:max(genes_at_heigh$X39))){
    
    genes_at_heigh_a<-genes_at_heigh[genes_at_heigh$X39==j,]
    
    test.genes<-data.frame(a=rownames(genes_at_heigh_a), stringsAsFactors=FALSE)
    
    if (length(test.genes$a) >3){
      
      annotation.GO.BP.biomart_testgenes<-annotation.GO.BP.biomart[annotation.GO.BP.biomart$ensembl_gene_id %in% test.genes$a, ]
      
      if( length(unique(annotation.GO.BP.biomart_testgenes$ensembl_gene_id)) >= 3 )  {
        
        all_genes_numeric<-as.integer(all_genes$gene %in%test.genes$a)
        names(all_genes_numeric)<-all_genes$gene
        
        
        pwf<-try(nullp(all_genes_numeric, bias.data=annotation.genelength.biomart_vector, plot.fit=FALSE ), silent =TRUE)
        if ('try-error' %in% class(pwf)) { pwf<-try(nullp(all_genes_numeric,'bosTau4','ensGene', plot.fit=FALSE ) )}
        
        if(!('try-error' %in% class(pwf))){
          
          GO_BP_Cats<-goseq(pwf,gene2cat=annotation.GO.BP.biomart, method ="Wallenius", use_genes_without_cat=FALSE)
          
          GO_BP_Cats<-GO_BP_Cats[GO_BP_Cats$numDEInCat>2,]
          
          if( dim(GO_BP_Cats)[1] != 0) {
            
            GO_BP_Cats$BY_FDR<-p.adjust(GO_BP_Cats$over_represented_pvalue, method ="BY")
            GO_BP_Cats$height  <- "X39"
            GO_BP_Cats$cluster <- j
            
            GO_BP_Cats<-merge(GO_BP_Cats,annotation.GO.BP.biomart_testgenes, by.x="category", by.y="go_id", all.x=TRUE, all.y=FALSE)
            
            go.results2<-rbind(go.results2,GO_BP_Cats)
          }
        }
        rm(annotation.GO.BP.biomart_testgenes,GO_BP_Cats, test.genes)
      }
    }
  }

go.results3<-go.results2[go.results2$BY_FDR < 0.2,]
go.results3<-go.results3[with(go.results3, order(BY_FDR)), ]
go.results3<-merge(go.results3, annotation.ensembl.symbol, by.x="ensembl_gene_id", by.y="ensembl_gene_id", all.x=TRUE, all.y=FALSE)
go.results3_EET<-go.results3[with(go.results3, order(BY_FDR)), ]
```

The final output of the next section “go.results3\_ICAR” was used to produce Table S6

```
annotation.ensembl.symbol<-read.table("2017_12_20_annotation.ensembl.symbol.txt.bz2",stringsAsFactors=FALSE, header =TRUE, sep="\t",quote = "")
annotation.genelength.biomart<-read.table("2017_12_20_annotation.genelength.biomart.txt.bz2",stringsAsFactors=FALSE, header =TRUE, sep="\t",quote = "")
annotation.GO.biomart<-read.table("2017_12_20_annotation.GO.biomart.txt.bz2",stringsAsFactors=FALSE, header =TRUE, sep="\t",quote = "")

all_genes<-data.frame( gene=rownames(fpkm_endo_day_18_IC_AI), stringsAsFactors=FALSE )
rownames(all_genes)<-all_genes$gene

annotation.genelength.biomart <-annotation.genelength.biomart[with(annotation.genelength.biomart, order(ensembl_gene_id, -transcript_length)), ]
annotation.genelength.biomart<-annotation.genelength.biomart[!duplicated(annotation.genelength.biomart$ensembl_gene_id),]
annotation.genelength.biomart <- annotation.genelength.biomart[annotation.genelength.biomart$ensembl_gene_id %in% rownames(all_genes),] 
annotation.genelength.biomart_vector<-annotation.genelength.biomart[,2]
names(annotation.genelength.biomart_vector)<-annotation.genelength.biomart[,1]
annotation.GO.BP.biomart<-annotation.GO.biomart[annotation.GO.biomart$namespace_1003=="biological_process", c(1:2)]
annotation.GO.BP.biomart<-annotation.GO.BP.biomart[annotation.GO.BP.biomart$ensembl_gene_id %in% rownames(all_genes),]

genes_at_heigh<-cutree(geneTree_endo, h=clusters.cut)
genes_at_heigh<-data.frame(genes_at_heigh)

go.results2<-data.frame()

  
  for (j in seq(1:max(genes_at_heigh$X44))){
    
    genes_at_heigh_a<-genes_at_heigh[genes_at_heigh$X44==j,]
    
    test.genes<-data.frame(a=rownames(genes_at_heigh_a), stringsAsFactors=FALSE)
    
    if (length(test.genes$a) >3){
      
      annotation.GO.BP.biomart_testgenes<-annotation.GO.BP.biomart[annotation.GO.BP.biomart$ensembl_gene_id %in% test.genes$a, ]
      
      if( length(unique(annotation.GO.BP.biomart_testgenes$ensembl_gene_id)) >= 3 )  {
        
        all_genes_numeric<-as.integer(all_genes$gene %in%test.genes$a)
        names(all_genes_numeric)<-all_genes$gene
        
        
        pwf<-try(nullp(all_genes_numeric, bias.data=annotation.genelength.biomart_vector, plot.fit=FALSE ), silent =TRUE)
        if ('try-error' %in% class(pwf)) { pwf<-try(nullp(all_genes_numeric,'bosTau4','ensGene', plot.fit=FALSE ) )}
        
        if(!('try-error' %in% class(pwf))){
          
          GO_BP_Cats<-goseq(pwf,gene2cat=annotation.GO.BP.biomart, method ="Wallenius", use_genes_without_cat=FALSE)
          
          GO_BP_Cats<-GO_BP_Cats[GO_BP_Cats$numDEInCat>2,]
          
          if( dim(GO_BP_Cats)[1] != 0) {
            
            GO_BP_Cats$BY_FDR<-p.adjust(GO_BP_Cats$over_represented_pvalue, method ="BY")
            GO_BP_Cats$height  <- "X44"
            GO_BP_Cats$cluster <- j
            
            GO_BP_Cats<-merge(GO_BP_Cats,annotation.GO.BP.biomart_testgenes, by.x="category", by.y="go_id", all.x=TRUE, all.y=FALSE)
            
            go.results2<-rbind(go.results2,GO_BP_Cats)
            
          }
        }
        rm(annotation.GO.BP.biomart_testgenes,GO_BP_Cats, test.genes)
      }
    }
  }


go.results3<-go.results2[go.results2$BY_FDR < 0.2,]
go.results3<-go.results3[with(go.results3, order(BY_FDR)), ]
go.results3<-merge(go.results3, annotation.ensembl.symbol, by.x="ensembl_gene_id", by.y="ensembl_gene_id", all.x=TRUE, all.y=FALSE)
go.results3_ICAR<-go.results3[with(go.results3, order(BY_FDR)), ]
```

## Figure 4b

```
genes_at_heigh<-cutree(geneTree_eet, h=clusters.cut)
genes_at_heigh<-data.frame(genes_at_heigh)
genes_at_heigh<-data.frame(genes_at_heigh)
genes_at_heigh_color_EET<-data.frame(genes_at_heigh)
genes_at_heigh_color_EET<-genes_at_heigh_color_EET[geneTree_eet$order,] 
genes_at_heigh_color_EET$X39[ !(genes_at_heigh_color_EET$X39 %in% unique(c(go.results3_EET$cluster)))]<- 0
data_frame_annotation_EET<-data.frame(cluster = genes_at_heigh_color_EET$X39)

colors_row_BP<-list(cluster=c(
  "0" = "#FFFFFF",
  "2" = "#4b3376",
  "3" = "#71d14b",
  "4" = "#924ed0",
  "5" = "#c8c54d",
  "7" = "#cb5592",
  "8" = "#78cb8f",
  "9" = "#ca4e37",
  "13" =    "#93c2c3",
  "17" =    "#593338",
  "20" =    "#c09061",
  "21" =    "#948fc2",
  "26" =    "#4b633c"
  
))


font_size=6

row_annotation <- HeatmapAnnotation(df = data_frame_annotation_EET, col = colors_row_BP, which="row", width = unit(0.4, "cm"),gap = unit(0, "mm"),
                                    annotation_legend_param=list(title="EET clusters",legend_direction="horizontal",nrow = 2,title_gp = gpar(fontsize = font_size), labels_gp = gpar(fontsize = font_size), grid_height = unit(2, "mm"), grid_width = unit(2, "mm")))


clusters.cut<-seq(1,50, 1) 
genes_at_heigh<-cutree(geneTree_endo, h=clusters.cut)
genes_at_heigh<-data.frame(genes_at_heigh)

genes_at_heigh<-data.frame(genes_at_heigh)
genes_at_heigh_color_ICAR<-data.frame(genes_at_heigh)
genes_at_heigh_color_ICAR<-genes_at_heigh_color_ICAR[geneTree_endo$order,] 
genes_at_heigh_color_ICAR$X44[ !(genes_at_heigh_color_ICAR$X44 %in% unique(c(go.results3_ICAR$cluster)))]<- 0
data_frame_annotation_ICAR<-data.frame(cluster = genes_at_heigh_color_ICAR$X44)

colors_column_BP<-list(cluster=c(
  "0" = "#FFFFFF",
  "1" = "#4dad98",
  "2" = "#7aa444",
  "3" = "#71d14b",
  "4" = "#cb547b",
  "6" = "#c5793e",
  "7" = "#cb5592",
  "8" = "#78cb8f"
))

column_annotation <- HeatmapAnnotation(df = data_frame_annotation_ICAR, col = colors_column_BP, which="column", height=unit(0.4, "cm"),width = unit(0.1, "cm"),gap = unit(0, "mm"),
                                       annotation_legend_param=list(title="ICAR clusters",title_position = "topcenter",legend_position="center",legend_direction="horizontal",nrow = 2,title_gp = gpar(fontsize =font_size),labels_gp = gpar(fontsize = font_size),grid_height = unit(2, "mm"), grid_width = unit(2, "mm")))

pearson_correlation_EET_heatmap<-pearson_correlation_EET[geneTree_eet$order,geneTree_endo$order ]

pearson_correlation_EET_heatmap[(pearson_correlation_EET_heatmap >= -0.95 &  pearson_correlation_EET_heatmap <= 0.95)]<-0

heatmap_pearson_eet_ICAR<- Heatmap(pearson_correlation_EET_heatmap,
        name="correlation",
        cluster_rows= FALSE,
        cluster_columns = FALSE,
        show_row_names = FALSE,
        show_column_names = FALSE,
        col = colorRamp2(c(-1, 0, 1), c("blue", "white", "red")),
        bottom_annotation=column_annotation
)

draw(heatmap_pearson_eet_ICAR + row_annotation , annotation_legend_side = "bottom",heatmap_legend_side = "bottom")
```

```
sessionInfo()
```

```
## R version 3.5.1 (2018-07-02)
## Platform: x86_64-pc-linux-gnu (64-bit)
## Running under: Ubuntu 18.04.1 LTS
## 
## Matrix products: default
## BLAS: /usr/lib/x86_64-linux-gnu/blas/libblas.so.3.7.1
## LAPACK: /usr/lib/x86_64-linux-gnu/lapack/liblapack.so.3.7.1
## 
## locale:
##  [1] LC_CTYPE=en_US.UTF-8       LC_NUMERIC=C               LC_TIME=en_US.UTF-8        LC_COLLATE=en_US.UTF-8     LC_MONETARY=en_US.UTF-8    LC_MESSAGES=en_US.UTF-8    LC_PAPER=en_US.UTF-8       LC_NAME=C                  LC_ADDRESS=C               LC_TELEPHONE=C             LC_MEASUREMENT=en_US.UTF-8 LC_IDENTIFICATION=C       
## 
## attached base packages:
##  [1] stats4    grid      parallel  stats     graphics  grDevices utils     datasets  methods   base     
## 
## other attached packages:
##  [1] knitr_1.20             vegan_2.5-3            lattice_0.20-38        permute_0.9-4          ggpubr_0.1.8           magrittr_1.5           org.Bt.eg.db_3.7.0     AnnotationDbi_1.44.0   IRanges_2.16.0         S4Vectors_0.20.0       scales_1.0.0           parallelDist_0.2.2     edgeR_3.24.0           limma_3.38.2           dendextend_1.9.0       ggrepel_0.8.0          circlize_0.4.4         ComplexHeatmap_1.20.0  Rtsne_0.13             VennDiagram_1.6.20     futile.logger_1.4.3    biomaRt_2.38.0         gtools_3.8.1           bigmemory_4.5.33       doParallel_1.0.14      iterators_1.0.10       foreach_1.4.4          gplots_3.0.1           flashClust_1.01-2      goseq_1.34.0           geneLenDataBase_1.18.0 BiasedUrn_1.07         multtest_2.38.0        Biobase_2.42.0         BiocGenerics_0.28.0    reshape_0.8.8          ggplot2_3.1.0          WGCNA_1.66             fastcluster_1.1.25     dynamicTreeCut_1.63-1  rmarkdown_1.10        
## 
## loaded via a namespace (and not attached):
##   [1] backports_1.1.2             Hmisc_4.1-1                 plyr_1.8.4                  lazyeval_0.2.1              splines_3.5.1               BiocParallel_1.16.0         GenomeInfoDb_1.18.0         robust_0.4-18               digest_0.6.18               htmltools_0.3.6             viridis_0.5.1               GO.db_3.7.0                 gdata_2.18.0                checkmate_1.8.5             memoise_1.1.0               fit.models_0.5-14           cluster_2.0.7-1             Biostrings_2.50.1           RcppParallel_4.4.1          matrixStats_0.54.0          prettyunits_1.0.2           colorspace_1.3-2            blob_1.1.1                  rrcov_1.4-4                 dplyr_0.7.7                 crayon_1.3.4                RCurl_1.95-4.11             bigmemory.sri_0.1.3         bindr_0.1.1                 impute_1.56.0               survival_2.43-1             glue_1.3.0                  gtable_0.2.0                zlibbioc_1.28.0             XVector_0.22.0              GetoptLong_0.1.7            DelayedArray_0.8.0          kernlab_0.9-27              shape_1.4.4                 prabclus_2.2-6              DEoptimR_1.0-8              futile.options_1.0.1        mvtnorm_1.0-8               DBI_1.0.0                   Rcpp_1.0.0                  viridisLite_0.3.0           progress_1.2.0              htmlTable_1.12              foreign_0.8-71              bit_1.1-14                  mclust_5.4.1                preprocessCore_1.44.0       Formula_1.2-3               htmlwidgets_1.3             httr_1.3.1                  RColorBrewer_1.1-2          fpc_2.1-11.1                acepack_1.4.1               modeltools_0.2-22           pkgconfig_2.0.2             XML_3.98-1.16               flexmix_2.3-14              nnet_7.3-12                 locfit_1.5-9.1              labeling_0.3                tidyselect_0.2.5            rlang_0.3.0.1               munsell_0.5.0               tools_3.5.1                 RSQLite_2.1.1               evaluate_0.12               stringr_1.3.1               yaml_2.2.0                  bit64_0.9-7                 robustbase_0.93-3           caTools_1.17.1.1            purrr_0.2.5                 bindrcpp_0.2.2              nlme_3.1-137                whisker_0.3-2               formatR_1.5                 compiler_3.5.1              rstudioapi_0.8              tibble_1.4.2                pcaPP_1.9-73                stringi_1.2.4               GenomicFeatures_1.34.1      trimcluster_0.1-2.1         Matrix_1.2-15               pillar_1.3.0                GlobalOptions_0.1.0         cowplot_0.9.3               data.table_1.11.8           bitops_1.0-6                rtracklayer_1.42.0          GenomicRanges_1.34.0        R6_2.3.0                    latticeExtra_0.6-28         KernSmooth_2.23-15          gridExtra_2.3               codetools_0.2-15            lambda.r_1.2.3              MASS_7.3-51.1               assertthat_0.2.0            SummarizedExperiment_1.12.0 rprojroot_1.3-2             rjson_0.2.20                withr_2.1.2                 GenomicAlignments_1.18.0    Rsamtools_1.34.0            GenomeInfoDbData_1.2.0      diptest_0.75-7              mgcv_1.8-25                 hms_0.4.2                   rpart_4.1-13                class_7.3-14                base64enc_0.1-3
```
